# DeSiphering receptor core-induced and ligand-dependent conformational changes in arrestin via genetic encoded trimethylsilyl ^1^H-NMR probe

**DOI:** 10.1038/s41467-020-18433-5

**Published:** 2020-09-25

**Authors:** Qi Liu, Qing-tao He, Xiaoxuan Lyu, Fan Yang, Zhong-liang Zhu, Peng Xiao, Zhao Yang, Feng Zhang, Zhao-ya Yang, Xiao-yan Wang, Peng Sun, Qian-wen Wang, Chang-xiu Qu, Zheng Gong, Jing-yu Lin, Zhen Xu, Shao-le Song, Shen-ming Huang, Sheng-chao Guo, Ming-jie Han, Kong-kai Zhu, Xin Chen, Alem W. Kahsai, Kun-Hong Xiao, Wei Kong, Fa-hui Li, Ke Ruan, Zi-jian Li, Xiao Yu, Xiao-gang Niu, Chang-wen Jin, Jiangyun Wang, Jin-peng Sun

**Affiliations:** 1 Institute of Biophysics, Chinese Academy of Sciences, 15 Datun Road, Chaoyang district, Beijing, 100101 China; 2 Key Laboratory Experimental Teratology of the Ministry of Education and Department of Physiology, School of Basic Medical Sciences, Cheeloo college of Medicine, Shandong University, 44 Wenhua Xi Road, Jinan, Shandong 250012 China; 3 Key Laboratory Experimental Teratology of the Ministry of Education and Department of Biochemistry and Molecular Biology, School of Basic Medical Sciences, Cheeloo college of Medicine, Shandong University, 44 Wenhua Xi Road, Jinan, 250012 Shandong China; 4 Key Laboratory of Molecular Cardiovascular Science, Ministry of Education, Peking University, 15 Xueyuan Road, Haidian District, Beijing, 100191 China; 5 School of Life Sciences, University of Science and Technology of China, 96 Jinzhai Road, Hefei, Anhui 230026 China; 6 Wuhan Institute of Physics and Mathematics, Chinese Academy of Sciences, 30 Xiaohongshan Road, Wuchang District, Wuhan, Hubei 430071 China; 7 Tianjin Institute of Industrial Biotechnology, Chinese Academy of Sciences, 32 Xiqi Road, Airport Economic Zone, Dongli District, Tianjin, 300308 China; 8 School of Biological Science and Technology, University of Jinan, 336 Nanxinzhuangxi Road, Shizhong District, Jinan, 250022 China; 9 Department of Medicinal Chemistry, School of Pharmaceutical Engineering and Life Science, Changzhou University, Changzhou, Jiangsu 213164 China; 10 Duke University, School of Medicine, Durham, NC 27705 USA; 11 Department of Pharmacology and Chemical Biology, School of Medicine, University of Pittsburgh, Pittsburgh, PA 15261 USA; 12 Hefei National Laboratory for Physical Science at the Microscale, University of Science and Technology of China, 443 Huangshan Road, Hefei, Anhui 230027 China; 13 Beijing Nuclear Magnetic Resonance Center, College of Chemistry and Molecular Engineering, School of Life Sciences, Peking University, Beijing, 100084 China; 14 College of Life Sciences and School of Future Technology, University of Chinese Academy of Sciences, Beijing, 100049 China

**Keywords:** Biochemistry, Biophysics, Chemical biology

## Abstract

Characterization of the dynamic conformational changes in membrane protein signaling complexes by nuclear magnetic resonance (NMR) spectroscopy remains challenging. Here we report the site-specific incorporation of 4-trimethylsilyl phenylalanine (TMSiPhe) into proteins, through genetic code expansion. Crystallographic analysis revealed structural changes that reshaped the TMSiPhe-specific amino-acyl tRNA synthetase active site to selectively accommodate the trimethylsilyl (TMSi) group. The unique up-field ^1^H-NMR chemical shift and the highly efficient incorporation of TMSiPhe enabled the characterization of multiple conformational states of a phospho-β2 adrenergic receptor/β-arrestin-1(β-arr1) membrane protein signaling complex, using only 5 μM protein and 20 min of spectrum accumulation time. We further showed that extracellular ligands induced conformational changes located in the polar core or ERK interaction site of β-arr1 via direct receptor transmembrane core interactions. These observations provided direct delineation and key mechanism insights that multiple receptor ligands were able to induce distinct functionally relevant conformational changes of arrestin.

## Abstract (web summary)

Characterization of dynamic conformational changes in membrane protein complexes by NMR spectroscopy remains challenging. Here authors report the site-specific incorporation of 4-trimethylsilyl phenylalanine (TMSiPhe) into proteins, which enabled the characterization of multiple conformational states of a phospho-β2 adrenergic receptor/β-arrestin-1 complex in response to different receptor ligands.

## Introduction

Membrane proteins account for ~30% of all proteins in living cells, and play critical roles such as material transportation and signal transduction. Because of their critical function in physiological processes, membrane proteins have become one of the most attractive research areas^1–3^. However, characterization of multiple functionally important conformation states in large membrane protein complexes has remained to be very challenging^4–6^.

Solution NMR is a powerful tool for studying the dynamics of membrane protein complexes. The developments of isotopic labeling strategies and mutidimensional NMR methods have facilitated the study of protein complexes up to one million Dalton^7^. However, the application of these methods, especially the assignment of complicated multidimensional spectrum is expensive and technically challenging for most biochemistry laboratories. It has recently emerged that one-dimensional ^19^F nuclear magnetic resonance (1D ^19^F-NMR) is a powerful and convenient method for studying dynamic conformation changes and post-translational modification of proteins^8–13^. The advantage of this method is that, through site-specific labeling, typically only one peak is present in the 1D NMR spectra. This allows for the facile characterization of dynamic conformation change with residual precision, without requiring for time-consuming and tedious NMR signal assignment. Despite this significant progress, ^19^F-NMR requires large amount of protein (usually >100 µm), and each measurement generally takes >12 hours. Therefore, the development of a chemical biological approach for examination of the conformational dynamics of transmembrane protein complexes using a low concentration of protein is urgently required. To address these challenges, Otting and colleagues have recently reported one-dimensional ^1^H NMR (1D ^1^H-NMR) tert-butyltyrosine probes. While the nine proton singlet from the tert-butyl group give rise to strong ^1^H-NMR signals, its chemical shift ~1.3 ppm overlaps strongly with the methyl group ^1^H-NMR signals of proteins, and is often difficult to assign. By contrast, the ^1^H-NMR signal from trimethylsilyl (TMS) group has a chemical shift ~0 ppm, which is free of other ^1^H-NMR signal typically present in proteins. Using a cell-free translation system, and a low-efficiency, promiscuous cyanophenylalanine-tRNA synthetase, Otting et al. reported the site-specific labeling of proteins using 4-(trimethylsilyl)phenylalanine (TMSiPhe)^14^. However, it was observed that ^1^H-NMR signal from TMSiPhe in labeled protein was about ten times smaller than expected, which may be attributed to limited compatibility of the cyanophenylalanine-tRNA synthetase with TMSiPhe. Indeed, no mass spectrometry result was shown to delineate the identity of the genetically incorporated amino acid, in response to UAG codon. Moreover, it was stated that cell-free translation system works best for proteins smaller than 50 kDa, and site-specific labeling of TMSiPhe on larger proteins was unsuccessful, presumably owing to the lack of protein chaperons.

To make the 1D ^1^H-NMR method broadly applicable for the investigation of dynamic conformation change in both small proteins and large membrane protein complexes, we report the highly efficient and selective incorporation of TMSiPhe in proteins in *Escherichia coli* cells. The application of the genetic coding of TMSiPhe and its combination with the 1D ^1^H-NMR spectrometry for detection of conformational change in a biological system was named as DeSipher method, and is used for specific incorporation of Si into the desired protein. The Key to the success of this method was the identification of a mutant *Methanococcus jannaschii* tyrosyl-tRNA synthetase (TyrRS) that exhibits high specificity toward 4-trimethylsilyl phenylalanine (TMSiPhe) in *E. coli* cells. Notably, crystallographic analysis revealed structural changes that reshaped the TMSiPhe-specific amino-acyl tRNA synthetase (TMSiPheRS) to accommodate the large TMS group. We show that TMSiPhe is genetically incoporated into the protein selectively by the UAG codon and that the UAG codon only encode TMSiPhe^14^. Owing to the high efficiency and fidelity of TMSiPheRS, we then applied this method to investigate the activation mechanism of arrestin, an important signal transducer downstream of most G-protein-coupled receptors (GPCRs)^12,13,15–23^. In addition to desensitize the receptor, arrestins are active signaling hubs of GPCRs by scaffolding receptor signaling complexes, which recruit multiple downstream effectors, such as kinases, phosphatases, ion channels etc^12,13,15–21,24,25^. Ligands engaged with particular receptor that elicited specific arrestin functions provided a potential alternative avenue for disease treatment. Although recent crystallographic and Cryo-EM studies have provided important knowledge of the interactions of the receptor with arrestin^26–28^, and the structural alterations of arrestins in response to the phospho-C-tail have also been observed by ^19^F-NMR spectroscopy in our previous work^12,13,29^; however, the specific receptor core engaged arrestin conformational changes that occurred at specific arrestin functional sites have not been fully investigated. Here, we demonstrate that DeSipher is useful for characterization of the partner binding affinity and the activation procedure of arrestin. Moreover, application of the DeSipher method to study the β2 adrenergic receptor (β2AR)/arrestin signaling complex enabled us to not only monitor multiple conformational states of specific arrestin regions but also provide direct experimental evidence that the distinct ligands on the same receptor drive functionally relevant conformations via direct receptor core/arrestin interactions.

## Results

### TMSiPhe-specific amino-acyl tRNA synthetase (TMSiPheRS)

**Fig. 1:**
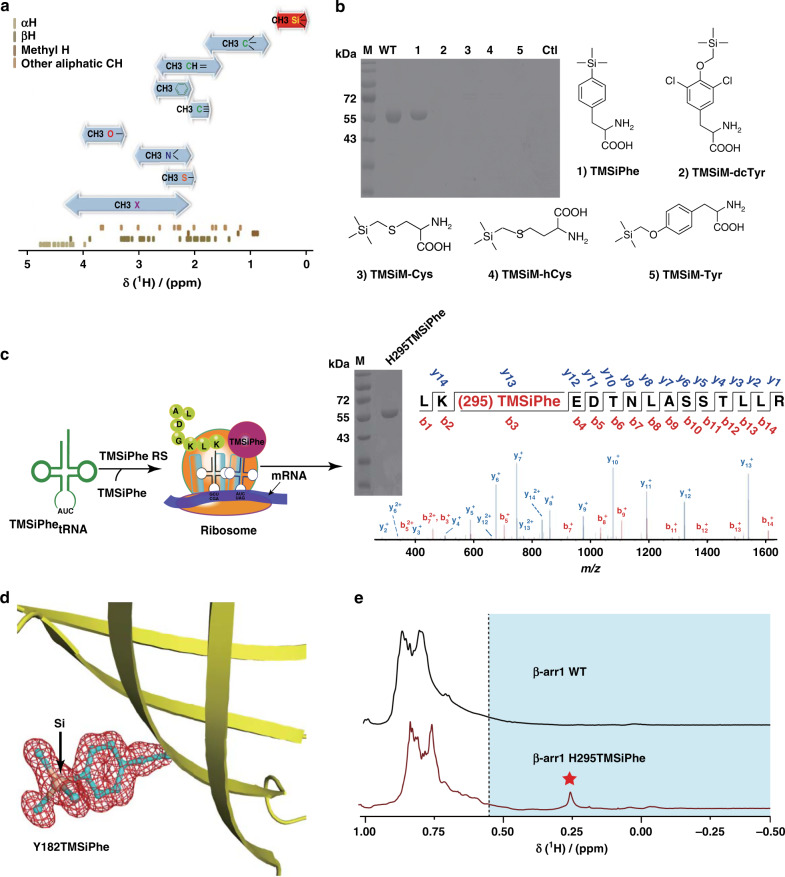
Development of TMSiPheRS by genetic code expansion and the selectivity of TMSiPheRS. **a** The ranges of the methyl ^1^H chemical shifts^64^ (shown by bidirectional arrows) and the distribution of random-coil aliphatic CH ^1^H chemical shifts for the 20 genetically coded amino acids^65^. The ^1^H chemical shifts of methyl silicon group are specified in red. **b** Coomassie-stained gel analysis of full-length β-arr1 expression in *E. coli* cells that were co-transformed with the β-arr1-H295 TAG plasmid and the pEVOL-TMSiPheRS plasmid in the presence or absence of different silicon-containing compounds. WT: β-arr1 wild-type. Full-length β-arr1 protein was obtained only in the presence of TMSiPhe for TAG mutation of β-arr1 or WT. These results suggested that the evolved TMSiPheRS exhibited significant structural selectivity for TMSiPhe over other silicon-containing chemicals. The chemical abbreviations are as follows: (1) 4-(trimethylsilyl) phenylalanine, TMSiPhe; (2) 3,5-dichloro-4-[(trimethylsilyl) methoxy]phenylalanine, TMSiM-dcTy; (3) 2-amino-3-((trimethylsilyl)methylthio)propanoic acid, TMSiM-Cys; (4) 2-amino-4-((trimethylsilyl)methylthio)butanoic acid, TMSiM-hCys; (5) 4-[(trimethylsilyl)ethoxy]phenylalanine, TMSiM-Tyr; (6), Ctl: control, no TMSiPhe added to the culture. **c** Schematic flowchart for the incorporation of TMSiPhe into β-arr1 at the H295 site. Full-length β-arr1 protein was obtained by co-transformation with the β-arr1-H295 TAG mutant plasmid and the pEVOL-TMSiPheRS plasmid, with TMSiPhe supplementation of the culture medium. The purity of the protein was determined by electrophoresis. The protein was subjected to trypsin digestion and analyzed by MS/MS. These results unambiguously confirmed that TMSiPhe was selectively incorporated into β-arr1 at the H295 position. m/z, mass/charge ratio. **d** The 2Fo-Fc annealing omit map of sfGFP Y182TMSiPhe clearly shows the electron density of TMSiPhe. The map was contoured at 1.1 σ. **e** 1D ^1^H NMR spectra for the β-arr1 H295TMSiPhe mutant were compared with β-arr1 WT cultured in the presence of TMSiPhe. The spectra were recorded in a buffer containing 50 mm Tris-HCl (pH = 7.5) and 150 mm NaCl at 25 °C using a Bruker 950 MHz NMR spectrometer. The β-arr1 H295TMSiPhe chemical shift at 0.26 ppm was consistent with the predicted chemical shift of the TMSi group. The ^1^H NMR signals of TMS group substituted amino acids in a protein were generally located in the high-field region (<0.55 ppm, blue area). Red pentagram: the position of NMR signal peak for the β-arr1-H295 TMSiPhe.

All genetically coded natural proteins in living organisms consist of five main elements, namely, carbon (C), nitrogen (N), oxygen (O), sulfur (S) and hydrogen (H), but not silicon (Si), which is one of the most abundant elements on earth. Detection of proton NMR signals for the H atoms attached to amide nitrogen atoms or to carbon atoms in these proteins, as well as multidimensional measurement or suppression of transverse relaxation, such as HSQC and TROSY^30–32^, enables structural and dynamic characterization of protein complexes in important physiological processes. However, information regarding the structural dynamics of membrane proteins remains challenging to obtain using ^1^H NMR spectroscopy owing to the difficulty of spectrum assignment from overlapping proton signals in the large protein complexes, the low signal-to-noise ratio (S/N) and the need for large amounts of membrane proteins. The chemical shifts of the H atoms attached to carbons are determined by the immediate environments. For instance, owing to the high electronegativity of oxygen, nitrogen and unsaturated carbon, the ^1^H NMR spectra of CH_3_-O, CH_3_-N, and CH_3_-C_sp2/sp_ normally exhibit low-field shifts between 1.5 ppm and 4.0 ppm. In contrast, the chemical shift of CH_3_ C_sp3_ is between 0.7 ppm and 1.8 ppm (Fig. 1a and Supplementary Fig. 1a). In particular, the low electronegativity of Si led to a uniquely low ^1^H chemical shift for CH_3_-Si (typically <0.55 ppm), which could be easily identified from the complicated ^1^H spectra of macro-biomolecules (Fig. 1a and Supplementary Fig. 1a, b). We therefore choose unnatural amino acid (UAA) TMSiPhe as a genetic code expansion target to exploit its unique proton NMR property to characterize conformational changes in biological systems (Fig. 1b).

The genetic code expansion technique has been widely used recently to incorporate unnatural amino acids at specific positions in a protein to enable in-depth investigation of many important biological processes^33–35^. Such a system includes a synthetic UAA, an orthogonal aaminoacyl tRNA synthetase (aaRS)-tRNA pair derived from directed evolution, and a host protein production organism^33–35^. We synthesized TMSiPhe to facilitate TMS group incorporation into protein, through an optimized route^36^ (Supplementary Fig. 2). TMSiPhe was then used for selection of a tRNA synthetase which accommodate this UAA, using a mutant library of *M. jannaschii* tyrosyl-tRNA synthetase (*Mj*-TyrRS)^37,38^. The mutant library of the *Mj*-TyrRS was designed by randomizing six active site residues (Y32, L65, F108, Q109, D158, and L162) that were within 6.5 Å of the tyrosine substrate, and by performing mutating one of the six residues I63, A67, H70, Y114, I159, and V164 to G, or keeping these residues unchanged as previously described^39^. In the positive selection, cell survival was dependent on the suppression of an amber mutation in the chloramphenicol acetyltransferase gene in the presence of TMSiPhe. By contrast, cells were eliminated if amber codons in the barnase gene was suppressed by natural amino acids in the negative selection without TMSiPhe. Following three rounds of positive selection and two rounds of negative selection^37^, a mutant *M. jannaschii* tyrosyl-tRNA synthetase (*Mj*-TyrRS) with specific activity toward TMSiPhe, termed TMSiPheRS, was identified. Sequence analysis revealed that the evolved TMSiPheRS harbors the mutations Y32H, I63G, L65V, H70Q, D158G, I159G, and V164G compared with wild-type *Mj*-TyrRS (Supplementary Fig. 3).

We next incorporated TMSiPhe into β-arr1, a signaling protein used here as a model system for evaluation of the TMSiPheRS method. Protein expression was carried out in the presence of β-arr1-H295 TAG plasmid, and the pEVOL-TMSiPheRS plasmid (which encodes both TMSiPheRS and *Mj*tRNA_CUA_^Tyr^) in *E. coli* grown in Luria-Bertani (LB) medium supplemented with 1 mm TMSiPhe. As negative controls, β-arr1 was also expressed in the absence of any UAA or in the presence of 1 mm other TMS group containing UAA (TMSiM-dcTyr, TMSiM-Cys, TMSiM-hCys, TMSiM-Tyr). Only cells transformed with the tRNA:aaRS pair for TMSiPhe and grown in the presence of TMSiPhe, but not other Si-containing amino acids, afforded full-length β-arr1 H295TMSiPhe protein after His tag affinity column purification, suggesting that the selected TMSiPheRS exhibited significant structural selectivity for TMSiPhe over other Si-containing unnatural amino acids (Fig. 1b). Mass spectrometric analysis unambiguously showed the incorporation of TMSiPhe at H295 position in β-arr1 with 100% selectivity (Fig. 1c, Supplementary Fig. 4 and Supplementary Table 1). Moreover, the electron density of TMSiPhe was clearly observed in the crystal structure of a TMSiPhe-containing green fluorescence protein (GFP), an easily crystallized protein (Fig. 1d, Supplementary Fig. 5 and Supplementary Table 2). Crystallization of GFP-Y182TMSiPhe takes ~1 week at 16 °C, thus confirming the stability of TMSiPhe after incorporation into a particular protein.

We then inspected the 1D ^1^H-NMR properties of the TMSiPhe decorated protein. NMR spectroscopy of β-arr1 H295TMSiPhe revealed a unique ^1^H-NMR peak at 0.25 ppm, which is well separated from the other endogenous ^1^H-NMR signals from β-arr1, providing a distinct NMR probe for the examination of the structural dynamics of a specific site (Fig. 1e and Supplementary Fig. 6). Notably, the ^1^H-NMR signal of TMSiPhe-incorporated arrestin can be detected at concentrations as low as 5 μm very rapidly (in <20 min) using a 950 MHz NMR spectrometer (Supplementary Fig. 7). This high sensitivity is mostly owing to the nine equivalent proton present in the TMS group, the non-chemical shift anisotropy (CSA) effect of proton and the usage of a high-field NMR spectrometer (Supplementary Fig. 7). In contrast, it is very hard to incorporate nine equal fluorine atoms in one unnatural amino acid for signal amplification by the ^19^F-NMR approach, and the large CSA of ^19^F in general limits ^19^F-NMR-based studies of proteins to NMR spectrometers with a resonant frequency lower than 600 MHz (Supplementary Fig. 7c). More importantly, 600 MHz and 800 MHz NMR instruments also produced facile assigned NMR signal with desired signal-to-noise ratio for TMSiPhe-incorporated β-arr1, despite the longer incubation times and higher protein concentration (Supplementary Fig. 7a, b). These results indicated that the TMSiPhe probe not only has broad application potential in general but also serves as a useful tool to detect conformations of large membrane proteins complexes utilizing high-field NMR, as such complexes are prone to aggregate at high concentrations.

### Selective recognition of TMSiPhe by TMSiPheRS

**Fig. 2:**
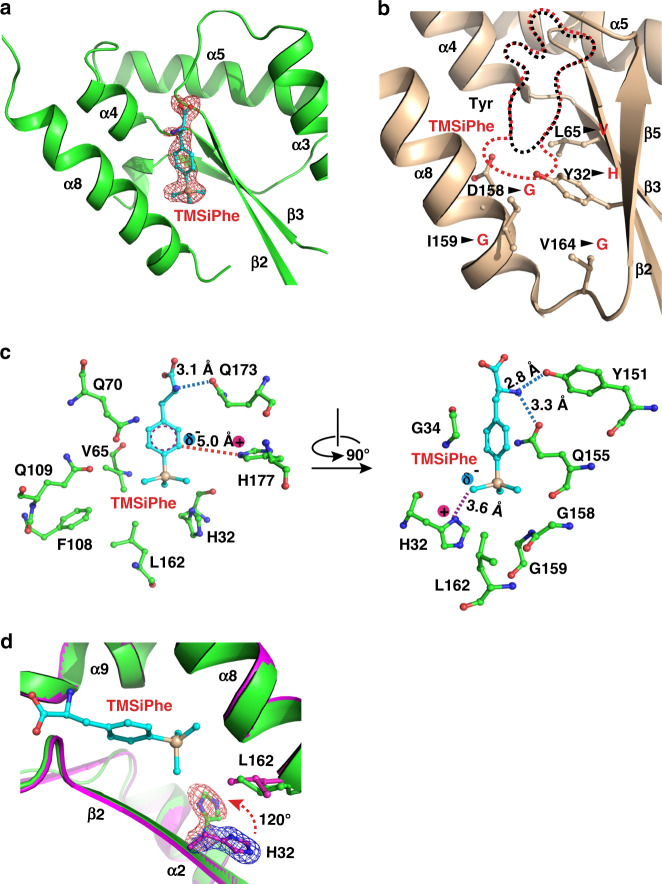
Structural basis for the selective recognition of TMSiPhe by TMSiPheRS. **a** Binding of TMSiPhe at the active site of TMSiPheRS. The 2Fo-Fc annealing omit electron density map of TMSiPhe was contoured at 1.0 σ. **b** Comparison of the unnatural amino acid-binding pockets between TMSiPheRS (red dotted line) and the wild-type *Mj*-TyrRS (black dotted line, PDB:1J1U). Five key mutations, indicated by arrows, increased the size of the TMSiPhe-binding pocket substantially. **c** Interactions between TMSiPhe and TMSiPheRS. The specific interactions include hydrogen bonds (blue dotted line), π-cation interactions (red dotted line), ion-dipole interactions (magenta dotted line) and hydrophobic interactions with surrounding residues (left panel). **d** The H32 residue in the β2 strand was rotated ~120 degrees in the TMSiPhe/TMSiPheRS complex (green) compared with TMSiPheRS alone (magenta), leading to favorable charged interactions with TMSiPhe. The 2Fo-Fc annealing omit map clearly shows the electron density of H32. The map was contoured at 1.0 σ.

To investigate the molecular basis of the selective recognition of TMSiPhe by TMSiPheRS, we crystallized TMSiPheRS and analyzed the structures by X-ray crystallography. The crystal structures of TMSiPheRS alone and the complex of TMSiPheRS with TMSiPhe were determined at 1.8 Å and 2.1 Å, respectively (Supplementary Table 2). The 2Fo-Fc annealing omit map of the TMSiPheRS/TMSiPhe complex unambiguously assigned the electron density for TMSiPhe (Fig. 2a and Supplementary Fig. 8). Introduction of the TMS group significantly increased the volume of the amino-acid substrate by ~60% (Fig. 2b). To compensate for this substantial change in volume, three residues, namely, D158, I159, and V164, were replaced by the smallest amino-acid glycine, and Y32 and L65 were substituted by the relatively small residues H32 and V65, respectively (Fig. 2b, c and Supplementary Fig. 3). We then compared the crystal structure of the TMSiPheRS/TMSiPhe complex with that of apo TMSiPheRS. Compared with the structure of TMSiPheRS alone, we observed a dramatic 120-degree rotation of histidine 32 in response to TMSiPhe binding. Moreover, Leu162 rotated ~42 degrees to form hydrophobic interactions with the methyl groups of TMSiPhe (Fig. 2d). Altogether, G34, V65, Q70, F108, Q109, Y151, Q155, G158, G159, Q173, and H177 defined a hydrophobic pocket for the accommodation of and specific interactions with the phenyl ring and TMS group of TMSiPhe (Fig. 2c and Supplementary Fig. 3). These observations provided a structural basis for specific and efficient incorporation of TMSiPhe using evolved TMSiPheRS.

### DeSipher β-arr1 activation by ^1^H-NMR

**Fig. 3:**
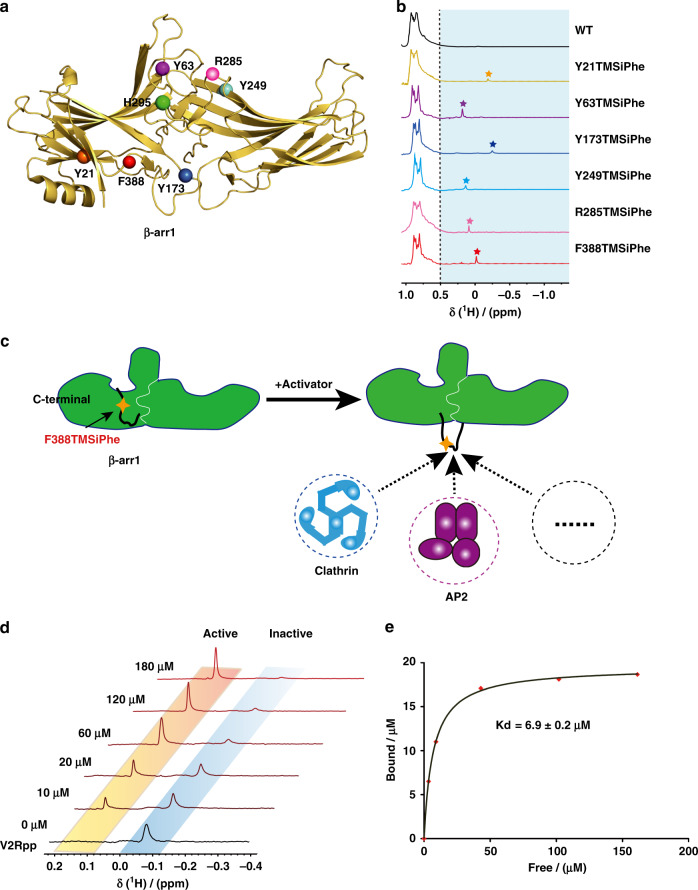
Incorporation of TMSiPhe at functionally relevant motifs of β-arr1. **a** Frontal view of the TMSiPhe incorporation sites depicted by spheres in the active β-arr1 crystal structure (PDB: 4JQI). Orange, Y21 in the three elements; purple, Y63 in the finger loop; blue, Y173 in the hinge region; cyan, Y249 in β-strand XVI; pink, R285, green, H295 in the lariat loop; red, F388 in the C-terminal swapping segment. **b** 1D ^1^H NMR spectra of β-arr1 labeled as described in (3a). The spectra were recorded in a buffer containing 50 mm Tris-HCl (pH = 7.5 and 150 mm NaCl at 25 °C using a Bruker 950 MHz NMR spectrometer. The protein concentrations were 5~15 μm, and the total recording time per spectrum was 6~15 min. The chemical shift for the TMSiPhe protein was less than 0.55 ppm. The pentagrams: the position of NMR signal peak for the β-arr1 inserted TMSiPhe at different sites. Orange: Y21; purple, Y63; blue, Y173; cyan, Y249; pink, R285; red, F388. **c** Cartoon illustration of the activation of β-arr1 and movement of the C-terminal swapping segment of β-arr1. In response to the binding of an activator, such as the phospho-vasopressin receptor C-tail (V2Rpp), the originally embedded C-terminal swapping segment of β-arr1 became highly solvent exposed, thus favoring binding to downstream signaling proteins, for example, clathrin or AP2 (adaptor protein 2). This conformational transition could be monitored by incorporation of TMSiPhe at the F388 position of β-arr1. **d** 1D ^1^H-NMR spectra of β-arr1 F388-TMSiPhe in response to titration with V2Rpp. Two distinct peaks were observed. The peak (−0.05 ppm), representing the inactive state gradually decreased in intensity, while the peak representing the active state (0.15 ppm) steadily increased in intensity. The spectra were recorded in a buffer containing 50 mm Tris-HCl (pH = 7.5) and 150 mm NaCl at 25 °C using a Bruker 950 MHz NMR spectrometer. **e** Analysis of the titration experiments monitored by 1D ^1^H-NMR spectroscopy of β-arr1-F388 TMSiPhe (3d). The curve was fitted to the nonlinear regression equation *y* = B_max_[X]/(Kd + [X]), according to the scatchard plot analysis (Supplementary Fig. 12). The Kd value was calculated at 6.9 ± 0.2 μm (*R*^2^ = 0.99).

We then incorporated TMSiPhe into functionally relevant structural motifs of β-arr1, the key signal transducer downstream of almost all 800 GPCRs encoded in the human genome, which functions not only by desensitizing membrane receptors but also by mediating independent downstream signaling after receptor activation^12,13,15–21,24,25^ (Fig. 3a, b). Although the functions of many arrestin-mediated receptors have been identified and certain motifs of arrestin are suspected to be involved in specific signaling pathways (Supplementary Table 3), the correlation between the conformational states of these arrestin motifs and selective receptor functions remains to be elucidated. Incorporation of TMSiPhe into β-arr1 at specific positions, including the receptor-phosphate-binding site (Y21), the finger loop (Y63), the hinge region (Y173), the β-strand XVI (Y249), the loop between β-strands XVIII and XVIIII (R285), the lariat loop (H295), and the C-terminal swapping region (F388), led to unambiguous assignment of NMR peaks between −0.3 ppm and 0.3 ppm in the ^1^H-NMR spectrum (Fig. 3b, Supplementary Figs. 9, 10 and Supplementary Table 4). These positions were proposed to be associated with specific arrestin functions, including receptor or IP6 interactions, the activation of downstream ERK or AP2, but have never been fully characterized by biophysical methods (Supplementary Table 3). Therefore, TMSiPhe-containing β-arr1 proteins provide a useful tool for monitoring conformational changes in arrestin in response to receptor activation or other stimuli. For example, the ^1^H-NMR spectrum of native β-arr1-F388 TMSiPhe exhibits a peak at −0.05 ppm, which can be easily identified. By contrast, when another UAA, O-tert-butyltyrosine^14,40^, was genetically encoded into the same position, the peaks for which cannot be assigned owing to strong overlap with the methyl signals from the protein (Supplementary Fig. 11). Notably, in response to stimulation with increased concentrations of phospho-vasopressin-2 receptor C-tail peptide (V2Rpp), the peak at −0.05 ppm gradually disappears, whereas a ^1^H-NMR peak at 0.15 ppm appears, reflecting the transition of the inactive arrestin conformation to an active arrestin conformation at the F388 position, through dislodgement of this specific C-terminal swapping segment (Fig. 3c, d). Moreover, the Scatchard plot for the titration experiment performed to examine the binding of V2R-phospho-C-tail to β-arr1 exhibits a straight line with a regression coefficient of 0.99. The calculated Kd value for the interaction of V2Rpp with β-arr1 was 6.9 ± 0.2 μm (Fig. 3e and Supplementary Fig. 12). Here, we demonstrate that while the genetic incorporation of TMSiPhe introduce little perturbation to the target protein, it can be used as a convenient tool for determining protein/peptide binding affinities. As the 1D ^1^H-NMR spectra contain only two peaks, which represent the active and inactive conformation, it takes little effort for NMR spectra assignment. Moreover, as ^1^H-NMR is easily accessible to most universities, our method is broadly applicable to most biochemistry laboratories.

### DeSipher ligands induced polar core conformational change

**Fig. 4:**
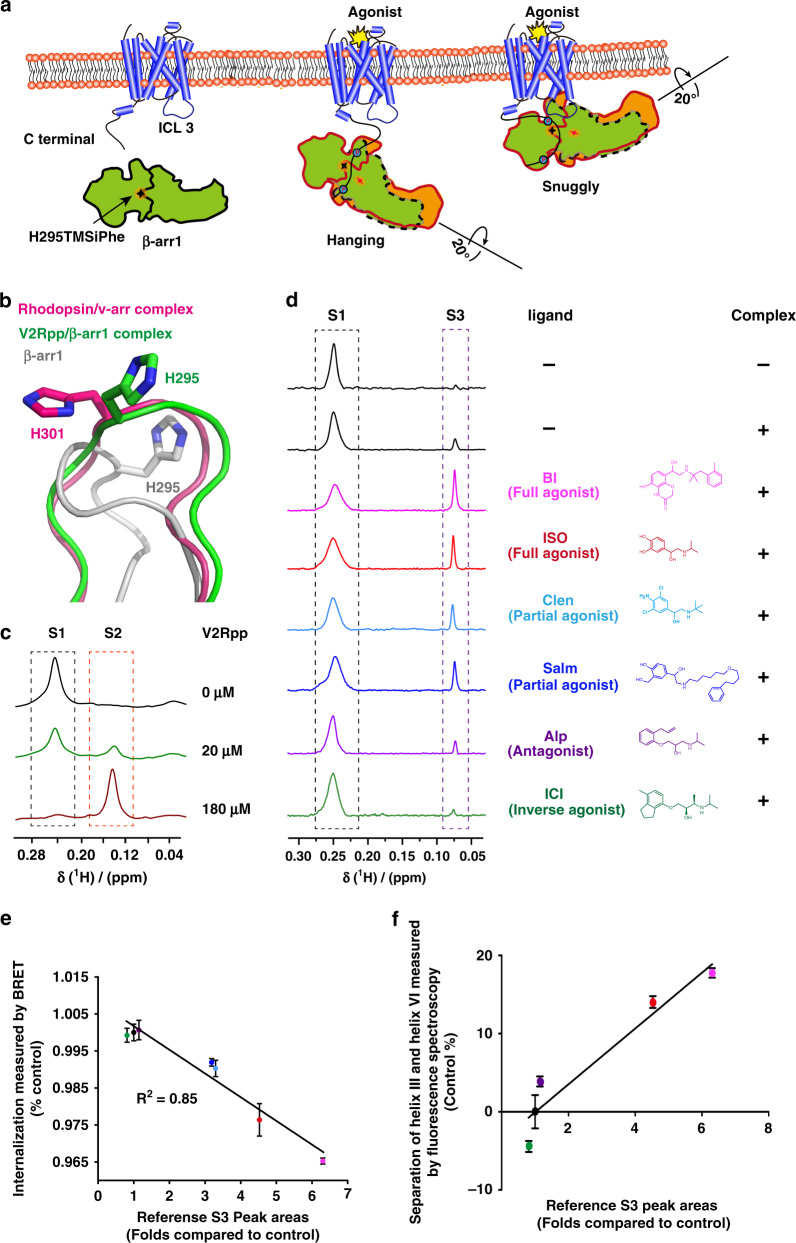
Regulation of the β-arr1 polar core by different β2AR ligands. **a** Cartoon illustration of two distinct interaction modes between GPCRs and β-arr1 (hanging mode and snug mode). The blue circles: phosphorylation. The Shuriken: the position of H295TMSiPhe in inactive (black) and active (red) β-arr1. **b** Structural comparison of the H295 position in inactive β-arr1 (PDB: 1G4M), the V2Rpp/ β-arr1 complex (PDB: 4JQI) and the rhodopsin/arrestin complex (PDB: 5W0P). The inactive β-arr1 structure is depicted in gray; the V2Rpp/β-arr1 complex is in green; and the rhodopsin-arrestin complex is in red. **c** 1D ^1^H NMR spectra of β-arr1-H295TMSiPhe in response to titration with V2Rpp. With increasing concentrations of V2Rpp, the peak at 0.25 ppm decreased (representing the S1 state), whereas a new growing peak was observed at 0.15 ppm (representing the S2 state). **d** 1D ^1^H NMR spectra of β-arr1 H295TMSiPhe alone or the ppβ2V2R/β-arr1 H295TMSiPhe/Fab30 complex with or without different ligands and the chemical structures of the ligands used in the current study. After incubation with the phospho-β2AR-V2-tail (ppβ2V2R) and formation of the receptor-arrestin complex, a new NMR signal appeared at 0.07 ppm (designated S3), and the intensity of the S1 peak decreased. When incubated with different β2AR ligands before formation of the ppβ2V2R-β-arr1/Fab30 complex, the S3 state signal intensity of the complex was positively correlated with effects of the ligands on the activation of downstream effectors, such as arrestin. *BI* BI-167107, *ISO* isoproterenol, *Clen* clenbuterol, salm salmeterol, *Alp* alprenolol; *ICI* ICI-118551. The buffer used for the experiment contained 20 mm HEPES, 150 mm NaCl, 0.01% LMNG, 0.002% CHS, and 10% D_2_O (pH = 7.5 at 25 °C). +: receptor-β-arr1 complex; β-arr1 alone. **e** Best-fit linear correlation of the peak area representing the amount of the S3 state in the presence of different ligands, with the ligand efficacy for receptor internalization from the BRET experiment in vivo. See Supplementary Fig. 22 for details. **f** Best-fit linear correlation of the peak area representing the amount of the S3 state in the presence of different ligands, with the ligand efficacy for separation of the receptor transmembrane III and VI from the TRIQ experiment in vitro^63^. See Supplementary Fig. 23 for details.

Arrestin is known to be activated via both receptor-phosphorylation and active seven transmembrane 7TM core^12,13,16–18,29,41–45^ (Fig. 4a). Although the recent rhodopsin/visual arrestin complex structure has provided a model of the interactions of the receptor core with arrestin at an atomic resolution^45^, there is little structural information regarding receptor core-induced structural rearrangement of arrestin at the residue level owing to technological difficulties in distinguishing the contributions of the receptor core, the receptor-phospho-tail, or the linker and arrestin mutant used in the crystal structures individually, as well as the large amount of the receptor complex required for structural delineation.

Thus, incorporation of TMSiPhe at specific positions in arrestin might facilitate detection of ligand-induced conformational changes in arrestin using 1D ^1^H-NMR DeSipher. One hallmark of arrestin activation is the ~20˚ twist between the N- and C-domains of the protein (Fig. 4a). In the inactive state, the N- and C-domains of β-arr1 are tethered by the polar core, which is composed of the extensive charged interactions of D26, R169, D290, D297, and R393 (Supplementary Fig. 13). Disruption of the salt bridge between D297 and R393 and that between D304 and R382, as well as the equivalent rhodopsin-visual arrestin interactions between D296 and R175 and D303 and R382, are known to activate arrestin^45^ (Supplementary Fig. 13). Notably, the results of recent molecular dynamics studies have indicated that the rotation of D296 of visual arrestin (D290 in β-arr1) is closely associated with interdomain twisting. We therefore incorporated TMSiPhe at the H295 position, which is close to both D290 and D297 of β-arr1, to monitor the receptor-induced conformational changes in the polar core (Fig. 4a, b and Supplementary Fig. 13).

Specific incorporation of TMSiPhe at the H295 position in β-arr1 did not impair the structural integrity of the protein, as β-arr1 H295TMSiPhe exhibited normal activation in response to the V2-receptor-phospho-tail interaction (Supplementary Fig. 14b). For structural validation of the DeSipher study with β-arr1 H295TMSiPhe, we performed ^1^H-NMR measurements using the conditions for the crystal structures of β-arr1 in both the inactive apo-arrestin and in active arrestin stabilized by vasopressin 2 receptor phospho-tail (V2Rpp) and the conformationally selective antibody Fab30^43^. Notably, in the two-step arrestin recruitment model of the receptor, V2Rpp/β-arr1 mostly exhibited the hanging mode, whereas the phospho-receptor/β-arr1 complex encompassing the core interaction represented the snuggly mode^41,43^ (Fig. 4a). Superimposition of the inactive and active arrestin structures revealed that both the hanging and snuggly modes of active arrestin had similar conformations at the H295 position, differing significantly from the modes of inactive arrestin, which featured considerable movement of the lariat loop (Fig. 4b).

In the inactive state, the DeSipher spectrum of β-arr1 H295TMSiPhe contained mainly one peak at 0.25 ppm, which was designated S1 (Fig. 4c). Upon increasing the concentration of V2Rpp, the peak volume of S1 gradually decreased, accompanied by the growth of a new peak at 0.15 ppm, which was designated S2. Addition of the Fab30, a selective antibody that recognizes only the active arrestin conformation, increased S2 but diminished S1 (Supplementary Fig. 15). The DeSipher spectrum obtained with a saturating concentration of V2Rpp or Fab30 mainly exhibited an S2 peak, indicating that S2 represented an active state of H295TMSiPhe, whereas S1 represented the inactive state of β-arr1 H295TMSiPhe (Fig. 4b, c).

We then inspected the conformational change at the H295 site in response to occupation of the receptor by a panel of ligands with the same phospho-receptor-tail by DeSipher using the β2 adrenergic receptor (β2AR) as a prototypic model. As previously described, we obtained the phospho-β2AR-V2-tail chimera (ppβ2V2R) by stimulating Sf9 cells with ISO (Isoproterenol), a low-affinity ligand, before harvesting the cells and washing out the residual ligands by affinity chromatography^46^. The purified ppβ2V2R was then incubated with various ligands and used to form a stable receptor/arrestin complex by further incubation with β-arr1 and the conformationally selective antibody fragment Fab30 (Supplementary Fig. 16). Complex formation was verified by size-exclusion chromatography, and DeSipher was performed to monitor changes in the NMR signal (Fig. 4d and Supplementary Fig. 17). Application of the arrestin active conformation stabilizing Fab30 alone had no significant effect on the NMR spectrum of inactive β-arr1 H295TMSiPhe (Supplementary Fig. 18). Notably, upon incubation with ppβ2V2R and Fab30, a new NMR signal appeared at 0.07 ppm (designated S3), which was associated with the decrease in the S1 peak (Fig. 4d and Supplementary Fig. 19). A titration experiment with increased concentration of ppβ2V2R indicated that the residual S1 resonances decreases in response to the presence of more ppβ2V2R (Supplementary Fig. 20). The weaker Kd value for ppβ2V2R compared with V2Rpp in derived from titration experiment may reflect the different phosphorylation states between the ppβ2V2R prepared by co-expression and the fully phosphorylated synthetic V2R peptide. Therefore, S3 may represent the active arrestin state of H295TMSiPhe in the presence of ppβ2V2R. The sharp S3 peak compared with S1 might indicate a highly solvent-exposed structure of the H295 state in β-arr1 after forming the complex with the ppβ2V2R, as observed in the crystal structure of the rhodopsin/visual arrestin complex. ^1^H-NMR chemical shift is sensitive to the change of hydrogen bonding, local dielectric constant, and nearby aromatic residues. Thus, NMR chemical shifts are sensitive to subtle structural changes in proteins. The S2 and S3 states of β-arr1 H295TMSiPhe have similar loop structures, but subtle differences in sidechain orientation are obvious (Fig. 4b).

We next examined the DeSipher spectrum of the ppβ2V2R/β-arr1 complex in the presence of various β2AR ligands exhibiting different pharmacological activities. Importantly, although the S1 state population of H295TMSiPhe decreased upon addition of various agonists, including the full agonists ISO and BI-167107, or the partial agonists clenbuterol (Clen) and salmaterol (Salm), the S3 state population increased (Fig. 4d and Supplementary Fig. 19, 21). In contrast, Alp and ICI-118551 both have very little effect on the S3 state compared with other partial or full agonists (Fig. 4d and Supplementary Fig. 19). Moreover, the volume of the S3 state corresponds to the potency of the ligand in inducing receptor internalization (Fig. 4e and Supplementary Fig. 22). These trends mirrored the ability of the ligand to promote torsion between helix VI and helix III, a hallmark of the conformational changes in the receptor 7-transmembrane core induced by agonists (Fig. 4f and Supplementary Fig. 23). Overall, the DeSipher spectrum of β-arr1 H295TMSiPhe indicated that the conformational changes in the polar core of the arrestin in response to ligand properties are associated with the abilities of these ligands to promote receptor internalization in the presence of the same receptor-phosphorylation barcode (Fig. 4e, f and Supplementary Fig. 22).

### 7TM core-mediated conformational change in β-arr1

**Fig. 5:**
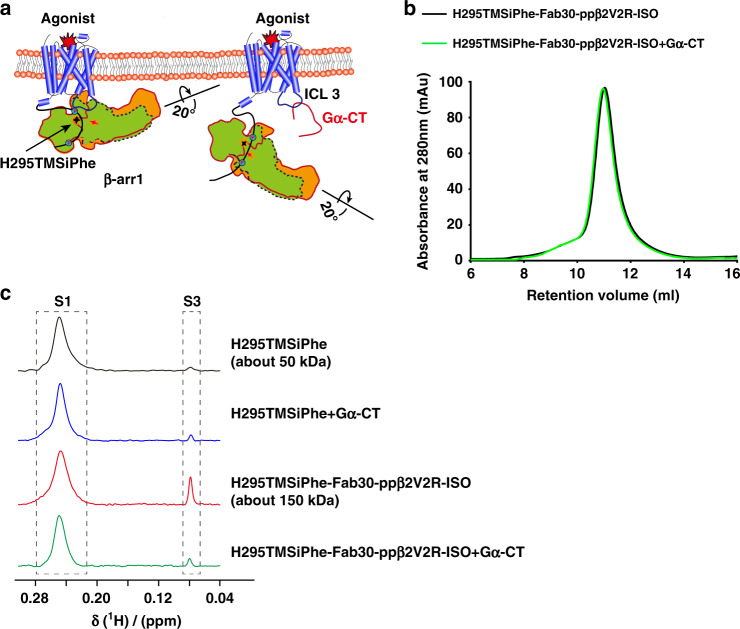
Gα-CT competition experiments. **a** Schematic diagram of the interaction between GPCRs and β-arr1 in the presence of excess Gα C-terminus (Gα-CT), which has been described in previous reports^47^. The interaction between β-arr1 and the GPCR TM core was abolished via steric hindrance by Gα-CT. β-arr1 still interacts with the phosphorylated GPCR C-terminal tail and thus forms a complex with the receptor. The blue circles indicate the phosphorylation. The Shuriken indicate the position of H295TMSiPhe in inactive (black) and active (red) β-arr1. **b** ISO/ppβ2V2R/β-arr1/Fab30 complex formation was not disrupted by Gα-CT in a size-exclusion assay. The similar SEC profile observed with or without Gα-CT suggests that Gα-CT did not disrupt the ISO/ppβ2V2R/β-arr1/Fab30 complex. Size-exclusion chromatography experiments were performed on an AKTA Purifier equipped with a Superdex 200 (10/300 GL) column. Black: ISO/ppβ2V2R/β-arr1-H295TMSiPhe/Fab30 complex, green: ISO/ppβ2V2R/β-arr1-H295TMSiPhe/Fab30 complex mixed with the 200 μm Gα-CT. **c** 1D ^1^H NMR spectra of β-arr1-H295TMSiPhe in the presence of Gα-CT. The transformation from S1 to S3 induced by the ISO/ppβ2V2R/β-arr1/Fab30 complex was significantly weakened by the addition of Gα-CT, suggesting the observed S3 state reduction was mainly caused to the elimination of the receptor core interaction with β-arr1 by the binding of Gα-CT.

To confirm that the observed S3 signal in the DeSipher spectrum was dependent on the interaction of β-arr1 with the receptor core, we performed a competition assay using a well-characterized binding partner of the receptor 7-transmembrane core, namely, the Gα protein C-tail (Gα-CT) (Fig. 5a). Recently, obtained crystal structures of the rhodopsin/arrestin, rhodopsin/Gα-CT, and rhodopsin/ArrFl (arrestin-finger loop peptide) complexes have revealed a significant overlapping interface of the receptor 7-transmembrane core with the finger loop of arrestin and Gα-CT of G-protein^45,47^. Moreover, direct engagement of the β-arr1 finger loop and Gα-CT of G-protein serves as a major interaction interface with the receptor 7-transmembrane core, which was supported by recent cross-linking and electron microscopic studies^43,48–50^. Therefore, we prepared the ISO/ppβ2V2R/β-arr1/Fab30 complex in the presence of Gα-CT. Incubation with Gα-CT did not disrupt the ISO/ppβ2V2R/β-arr1/Fab30 complex, as the presence of Gα-CT did not alter the SEC (size-exclusion chromatography) profile (Fig. 5b). Notably, although incubation of Gα-CT with β-arr1 H295TMSiPhe led to no significant alteration in the DeSipher spectrum, addition of Gα-CT with the ISO/ppβ2V2R/β-arr1/Fab30 complex significantly decreased the S3 state, suggesting that the observed S3 state reduction was mainly owing to elimination of the interaction of the receptor core with β-arr1 via the binding of Gα-CT (Fig. 5c). Although we predicted that the Gα-CT peptide competition would transition arrestin to a hanging conformation, we did not observe the appearance of the S2 state in the NMR spectrum. The loss of the NMR signal of this particular hanging arrestin conformation in our Gα-CT peptide competition experiments may be due to the effects of detergents. Further experiments using nanodiscs as a substitute for the detergent could help elucidate the hanging conformation of the arrestin^51^.

Because H295 is located in the close proximity to the polar core residues Asp290 and Asp297 of β-arr1, the DeSipher spectrum obtained from Gα-CT competition experiments with β-arr1 H295TMSiPhe confirmed that the agonist ISO was able to induce conformational changes in the polar core of β-arr1 via direct transmembrane core interactions.

### Conformational states at ERK interaction site of β-arr1

**Fig. 6:**
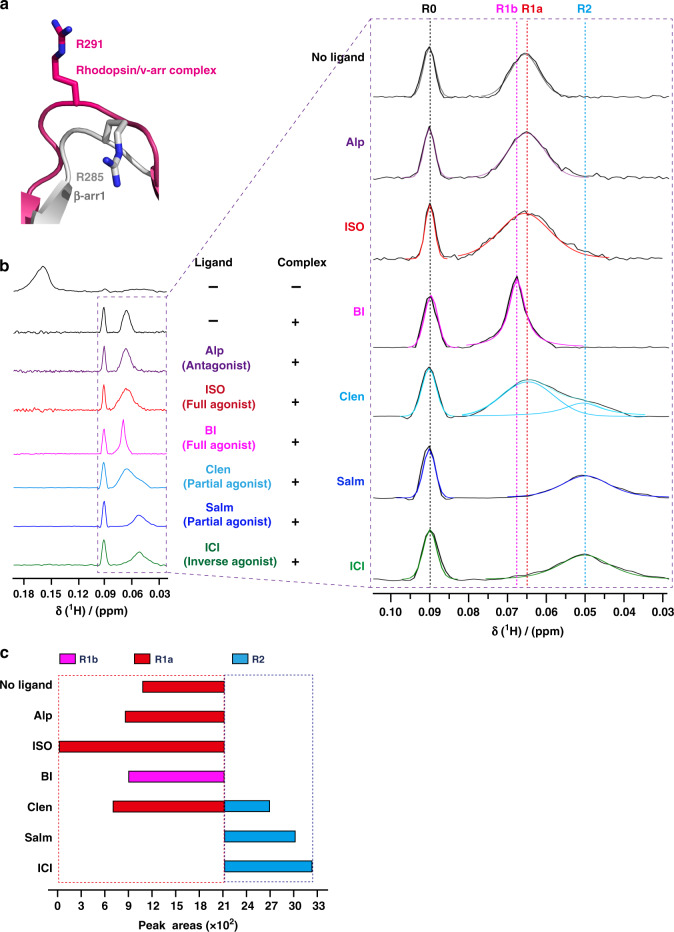
Monitoring the conformational states of site 285 of β-arr1. **a** Structural comparison of the R285 position in inactive β-arr1 (PDB: 1G4M) and the corresponding R291 position in the rhodopsin/arrestin complex (PDB: 5W0P). The active β-arr1 structure is depicted in gray, and the rhodopsin/arrestin complex is in red. The activation of arrestin by a receptor led to a highly solvent-exposed configuration at the R285 position of β-arr1, as suggested by the crystal structures. **b** 1D ^1^H NMR spectra of β-arr1 R285TMSiPhe activated by ppβ2V2R with or without different ligands. After incubation with ppβ2V2R, multiple new NMR signals appeared between 0.04 ppm and 0.10 ppm, which are designated as R0 (0.09 ppm), R1a (0.065 ppm), R1b (0.068 ppm), R2 (0.05 ppm), from low field to high field. The buffer used for the experiment contained 20 mm HEPES, 150 mm NaCl, 0.01% LMNG, 0.002% CHS, and 10% D_2_O (pH = 7.5 at 25 °C). +: receptor-β-arr1 complex; −: β-arr1 alone. **c** Bar graph representing the population (simulated peak area) of each NMR peak for each ligand condition. The values are also tabulated in Supplementary Fig. 26.

We next extended the TMSiPhe technology to study the conformations of other β-arr1 sites associated with specific arrestin functions. We selected the R285 position of β-arr1, which was hypothesized to play important roles in interaction with ERK^52^ (Fig. 3a and Supplementary Table 3). Notably, superimposition of the structures of inactive β-arr1 structure and the rhodopsin-visual arrestin complex indicated that R285 assumed a highly exposed and extended conformation (Fig. 6a), suggesting that receptor interaction may regulate conformational change at this specific site.

We therefore incorporated TMSiPhe at the R285 site of β-arr1 and monitored the change in the ^1^H-NMR spectrum in response to the binding of ppβ2V2R engaged with different ligands (Fig. 6b and Supplementary Figs. 25, 26). The functional integrity of β-arr1 R285TMSiPhe was validated, and Fab30 was used to stabilize the ppβ2V2R/β-arr1 complex without perturbation in the NMR spectrum (Supplementary Figs. 14a and 24). Application of ppβ2V2R without or with different ligands eliminated the original NMR peak at 0.158 ppm but broadened the conformational distributions from 0.03 ppm to 0.10 ppm (Fig. 6b, c and Supplementary Fig. 26). At least four different conformational states of the ppβ2V2R/β-arr1 R285TMSiPhe/Fab30 complex were discerned in the presence of different ligands. Notably, β-arr1 alone also has small but visible peaks in the 0.03 ppm–0.10 ppm region, indicating that a conformational selection model may also be suitable for description of the receptor-induced conformational change at the β-arr1 R285 position. In particular, addition of any receptor complexes without or with different ligands all produced a similar peak at 0.09 ppm (R0 state), indicating that this conformational state may be mainly owing to the binding of the receptor-phospho-tail but is not significantly affected by the receptor core interaction (Fig. 6b and Supplementary Fig. 26).

The ligands mostly changed the distribution of NMR peaks from 0.04 ppm to 0.07 ppm, which included three conformational states derived by simulation, namely, R1a-b (0.065–0.068 ppm) and R2 (0.05 ppm). Although application of the neutral antagonist Alp and the agonist ISO had no significant effect on the NMR peak at R1a (0.065 ppm), application of the long-term covalent agonist BI caused a small but significant low-field shift of R1a to R1b (0.068 ppm) (Fig. 6b, c and Supplementary Fig. 26).

The application of partial and inverse agonists caused complex conformational changes. Whereas Clen significantly diminished the distribution of the R1a state and promoted the appearance of a high-field R2 state, the engagement of the receptor with the G-protein-biased partial agonist Salm and the inverse agonist ICI almost completely eliminated the presence of the R1 states and facilitated the emergence of the R2 states (Fig. 6b, c and Supplementary Fig. 26). As Salm and ICI are not known for arrestin-dependent ERK signaling, the appearance of the R2 conformational states of the β-arr1 R285 position may not contribute to ERK activation in response to receptor/arrestin complex interactions. Taken together, multiple conformational states of the β-arr1 R285 position were detected by DeSipher in response to different β2AR ligands, which was not strictly correlated with the ability of these ligands in either the activation of G-protein (agonists vs. antagonists) or arrestin-mediated receptor internalization, indicating that each specific receptor ligand may lead to a distinct conformational state at a specific arrestin site, which contributes to the selective functions of these ligands.

### Observation of the conformational change of clathrin

**Fig. 7:**
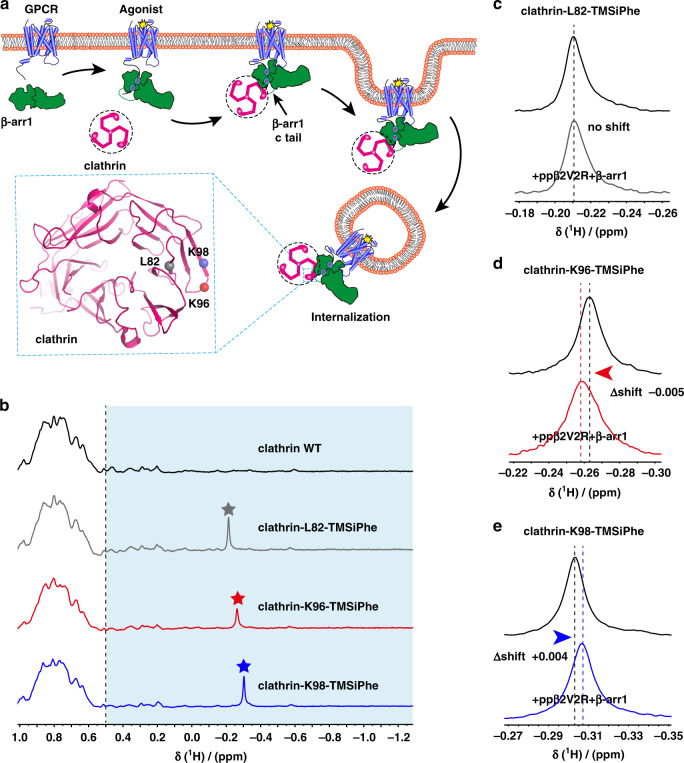
Incorporation of TMSiPhe in clathrin to monitor the conformation changes. **a** Schematic diagram of interaction between the clathrin and arrestin during the receptor endocytosis. β-arr1 is recruited to the receptor when the receptor is activated by specific agonists. The arrestin mediate receptor internalization by interacting with clathrin and AP2. The clathrin terminal domain (TD) (heavy chain) interacted with the c-tail of the β-arr1. Residues of L82, K96, and K98 are close to the arrestin/clathrin interface (clathrin crystal structure, PDB: 1BPO). The TMSiPhe-incorporated clathrin sites are depicted as spheres with different colors (gray: L82, red: K96, blue: K98). The blue circles indicate the phosphorylation. **b** 1D ^1^H NMR spectra of clathrin labeled as described in (7a). The spectra were recorded in a buffer containing 20 mm HEPES (pH = 7.5) 150 mm NaCl and 10% D_2_O at 25 °C using a Bruker 950 MHz NMR spectrometer. The protein concentrations were 10 μm, and the total recording time per spectrum was 18 min. The chemical shift for the TMSiPhe protein was <−0.1 ppm. **c**–**e** 1D ^1^H NMR spectra of clathrin alone and incubation with ISO-ppβ2V2R/β-arr1 complex. Clathrin-L82-TMSiPhe displayed no chemical shift in response to the incubation with the ppβ2V2R/β-arr1 complex. The clathrin-K96-TMSiPhe and clathrin-K98-TMSiPhe showed low-field shift (△shift = −0.005 ppm) and high-field shift (△shift = +0.004 ppm), respectively. The buffer used for the experiment contained 20 mm HEPES, 150 mm NaCl, 0.01% LMNG, 0.002% CHS, and 10% D_2_O (pH = 7.5 at 25 °C). The spectra were recorded using a Bruker 950 MHz NMR spectrometer.

To extend the application of the DeSipher method to another system, we incorporated the TMSiPhe into the clathrin, a critical endocytic protein that mediates the endocytosis of most GPCR endocytosis through engagement with the receptor-arrestin complexes^53–55^ (Fig. 7a). Clathrin interacts with the arrestin through the N-terminal domain of its heavy chain, forming direct contacts with the Lϕxϕ[D/E] motif located at the C-terminal of arrestin^55^. We therefore incorporated the TMSiPhe into positions of the N-terminal domain of the heavy chain of clathrin that are close to the interface with the receptor-arrestin complexes. Three TMSiPhe-containing clathrin proteins, namely, clathrin-L82-TMSiPhe, clathrin-K96-TMSiPhe and clathrin-K98-TMSiPhe, were purified to homogenous (Supplementary Fig. 27). Unambiguous peaks between −0.4 ppm and −0.1 ppm in the ^1^H-NMR spectrum were assigned to the DeSipher signals of each TMSiPhe-containing clathrin protein (Fig. 7b).

We next examined whether TMSiPhe-incorporated clathrins were able to sense the interaction of receptor-arrestin complexes. Although clathrin-L82-TMSiPhe displayed no chemical shift in response to incubation with the ppβ2V2R/β-arr1 complex, clathrin-K96-TMSiPhe, and clathrin-K98-TMSiPhe showed low-field shift or high-field shift, respectively (Fig. 7c). These observations suggested that the TMSiPhe-containing clathrin could serve as a useful tool to detect conformational changes in clathrin downstream of GPCR signaling.

## Discussion

Despite the broad applications of NMR in the characterization of protein structure and dynamics, it has remained very challenging to use NMR to study large transmembrane protein complexes, whose NMR spectra exhibit severe line broadening and overlapping resonance. Although site-specific protein labeling with 1D NMR probe has provided an exciting method for the investigation of membrane protein complex^8,9,31,56,57^, one limitation of cysteine-mediated chemical labeling is that it only allows access to the surface residues of proteins, preventing observation of the important dynamic interactions that occur within protein hydrophobic cores. Moreover, to achieve site-specific labeling, all other surface-exposed cysteine residues must be mutated, which may cause significant perturbation to protein structure and function. By contrast, UAA incorporation through genetic code expansion allows labeling of desired residues at both exposed and internal sites. For example, through genetic code expansion, we have developed a method to efficiently incorporate the UAA difluorotyrosine (F2Y) into proteins of interest, enabling us to study how different receptor phospho-barcodes localized in the receptor C-tail regulate distinct functionally selective arrestin conformations^12,13^. Despite this significant progress, the combination of ^19^F-NMR with the genetic expansion F2Y technology in general requires large amount of protein (usually >100 µm), and each measurement generally takes >12 hours for production of a suitable S/N. Therefore, the development of a chemical biological approach for examination of the conformational dynamics of transmembrane protein complexes using a low concentration of protein is urgently required.

Through genetic code expansion in *E. coli*, we have achieved the highly selective and efficient labeling of the TMS group in proteins and demonstrated its broad applicability in investigating multiple conformation statse of large membrane protein complexes. Efficient and selective incorporation of TMSiPhe was verified by both mass spectrometry and crystallography. Using this method, we were able to detect the dynamic conformational changes in the membrane protein complex (molecular weight ~150 kDa) at the residue level using a low protein concentration of ~5 μm, and a short spectra accumulation time of 20 min with a 950 MHz NMR spectrometer. Even 800 MHz or 600 MHz could provide the desired signal in a shorter time and with lower protein usage compared with the ^19^F-F2Y NMR probe that we developed previously^12,13,24^. The increased signal could be owing to the non-CSA effect of protons and the incorporation of the nine equivalent proton atoms in the TMSiPhe probe. In contrast to TMSiPhe, it is still very hard to integrate nine equal fluorine atoms in one specific UAA. Notably, key to this advance is the evolution of TMSiPheRS, a specific tRNA synthetase that selectively recognizes TMSiPhe to facilitate its genetic incorporation into proteins.

By applying our developed DeSipher method, we were able to observe the conformational changes of the membrane protein complex at residue resolution, for example, the ligand-dependent conformational changes in arrestin via direct receptor core engagement, a process important for GPCR signaling. Notably, biased ligands with the ability to selectively perform specific arrestin or G-protein subtype functions are crucial for the development of the next generation of drugs targeting GPCRs^15^. Previous studies by us and others have provided important mechanistic insights, demonstrating that receptor-phospho-barcodes present in the receptor C-tail play pivotal roles in the determination of selective arrestin functions^12,13,17,18,41–43,58^. An important hypothesis for the development of arrestin-biased GPCR ligands is that ligands for GPCRs can cause conformational changes in arrestin via direct receptor core/arrestin interactions regardless of the C-terminal phosphorylation pattern. Notably, the rhodopsin/visual arrestin complex crystal structure provided knowledge of receptor core/arrestin interactions at the atomic level^3^, and the fluorescent arsenical hairpin-bioluminescence resonance energy transfer (FlAsH-BRET) assays revealed that different receptor activation resulted in diverse arrestin conformations in cells^17^. However, dynamic information and high-resolution data regarding conformational changes in arrestin dictated by different receptor ligands via the receptor core/arrestin interaction remains undetermined, likely owing to the low resolution of cellular methods and the difficulty of the application of biophysical approaches for the study of receptor complex systems. Here, by using the residue-specific conformational detection method DeSipher, as well as fluorescence quenching experiments and cellular internalization assays, our experimental results suggested that ligands directed structural alterations of the seven-helix transmembrane core of the receptor via interactions with arrestin, were able to cause conformational change in the arrestin polar core at the specific residue H295, which is correlated with the internalization ability of the receptor/arrestin complex.

In addition to structural alterations in the polar core, we used DeSipher to examine the conformational changes that occurred at the R285 position of β-arr1,a site associated with ERK activation^52^. Importantly, R285TMSiPhe assumed multiple conformations in response to the engagement of different ligands with β2V2R harboring the same phosphorylated receptor C-tail. Importantly, the conformational states of the R285 site are not directly correlated to the functions of these β2AR ligands in either Gs activation or receptor internalization, indicating that different ligands of the same receptor were able to regulate distinct arrestin conformations at specific arrestin sites, which may be correlated with selective functions. Notably, the arrestin-ERK interaction may involve multiple interfaces. Therefore, the conformational changes in the R285 site observed by DeSipher likely contribute to, but are not the sole determinants of, arrestin-mediated ERK activation.

In summary, we have achieved the efficient and selection incorporation of TMSiPhe into protein in *E. coli*, to facilitate rapid detection of the dynamic conformational changes in 150 kDa membrane protein complexes, using 1D ^1^H-NMR. Owing to the high ^1^H-NMR signal intensity, and unique up-field chemical shift of the TMS group, good 1D ^1^H-NMR spectra can be acquired using only 5 μm of protein, and 20 min accumulation time. Using this handy and powerful approach, we identified the ligand-induced and functionally relevant arrestin conformational states via receptor core engagement. We expect this method will be broadly applicable to biochemistry laboratories to decipher dynamic protein interaction mechanism under physiological conditions.

## Methods

### Reagents

Anti-GST (Cell Signaling Technology, Catalog #2622), anti-His (Cell Signaling Technology, Catalog #2366), anti-Flag M2 (Sigma, Catalog: F3165). Anti-BV envelope gp64 PE antibody (eBioscience, Catalog: 12-6991-80). Secondary anti-rabbit antibody (Sigma Aldrich, Catalog #A6154) and secondary anti-mouse antibody (Sigma Aldrich, Catalog #A4416). The primary antibodies were used in 1:1000 dilution. The secondary antibodies were used in 1:5000 dilution. Glutathione-Sepharose 4B and Ni-NTA Agarose were from Amersham Pharmacia Biotech., isoproterenol, alprenolol, clenbuterol, salmaterol, and ICI-118551 were purchased from MCE. BI-167107 was synthesized by professor Xin Chen at Changzhou University. V2Rpp were synthesized by Tufts University core facility. All of the other reagents were from Sigma.

### Constructs

The full-length wild-type cDNAs of bovine β-arr1 was subcloned into the NdeI/XhoI sites of the pET22b vector with the C-terminal His tag. The β-arr1 mutations Y21TAG, Y63TAG, Y173TAG, Y249TAG, R285TAG, H295TAG, F388TAG, sfGFP Y182TAG were generated using the Quikchange mutagenesis kit (Stratagene). The pFast-β2V2R construct was created by replaced the C-terminal of pFast-β2AR using the last 29 amino acid cDNA of human V2 Vasopressin receptor (V2R). The pcDNA3.1-Flag-β2V2R-Rluc was created by in-fusion of the Rluc plasmid with the pcDNA3.1-Flag-β2V2R construct. All constructs and mutations were verified by DNA sequencing. All primers used in our study were shown in Supplementary Table 5.

### Synthesis of TMSiPhe

The synthesis of TMSiPhe according to the route in Supplementary Fig. 2, with following steps^36^.

Synthesis of trimethyl(4-tolyl) silane (2).

Iodine (catalytic amount) was added to the mixture of Magnesium turning (2.67 g, 110 mmol) and 4-bromotoluene 1 (1.71 g, 10 mmol) in 80 ml of dry tetrahydrofuran (THF (containing 0.002% water). The reaction was started by heating, then 4-bromotoluene (1) (15.4 g, 90 mmol, dissolved in 20 mL of dry THF) was slowly added in a drop wised manner. After refluxing for 4 h, the reactions were kept slight boiling by the drop wised addition of trimethyl chlorosilane (12.7 ml, 110 mmol). The mixture were reflux for another 2 h, followed by stirring at room temperature and quenching with 500 ml ice-cold water. The mixture was extracted with ethyl acetate (EA, 100 mL*3) and the organic layers were combined and subsequently washed with brine (100 mL*3). The organic layer was then dried over Na_2_SO_4_, filtered and evaporated. The residue was chromatographed by silica gel with petroleum ether as an eluent. The colorless liquid (14.3 g) was obtained with 87% yield.^1^H NMR (500 MHz, CDCl_3_) δ 7.45 (d, *J* = 7.6 Hz, 2H), 7.21 (d, *J* = 7.4 Hz, 2H), 2.38 (s, 3H), 0.28 (s, 9H).

Synthesis of (4-(bromomethyl) phenyl) trimethylsilane (3)

Trimethyl(4-tolyl) silane (2) (3.28 g, 20 mmol) was dissolved in tetrachloromethane (CCl_4_, 50 mL, A.R. grade) at room temperature. N-bromosuccinimide (3.56 g, 20 mmol) and azodiisobutyronitrile (0.33 g, 2 mmol) was added. The mixture was stirred with 4 hours refluxing, followed by vacuum condensation. The residue was used for the next step without further purification.

^1^H NMR (500 MHz, CDCl_3_) δ 7.51 (d, *J* = 7.9 Hz, 2H), 7.38 (d, *J* = 7.9 Hz, 2H), 4.51 (s, 2H), 0.28 (s, 9H).

Synthesis of ethyl 2-((diphenylmethylene)amino)-3-(4-(trimethylsilyl) phenyl) propanoate (4)

N-(Diphenylmethylene)glycine ethyl ester (13.37 g, 50 mmol) and potassium hydroxide (8.42 g,150 mmol) was dissolved in 60 ml dimethyl sulfoxide (DMSO) and the mixture was stirred at 10 °C for 20 min. The mixture was added with (4-(bromomethyl) phenyl) trimethylsilane (3) (12.15  g, 50 mmol) and kept stirring for 1 h, following by adding 720 ml of ice-cold water and then extracting with EA (200 ml*3). The organic layers were combined and were subsequently washed with brine (100 mL*3). The organic layer was then dried over Na_2_SO_4_, filtered, and concentrated under reduced pressure.

Synthesis of ethyl 2-amino-3-(4-(trimethylsilyl) phenyl) propanoate (5)

The residue from preceding step was added with THF 60 ml and 1 n HCl aqueous 60 ml. The solution was stirred for 1 h and then was added with 180 ml of PE, washed with PE/diethyl ether (3:1) (200 ml*3). The organic phase was extracted with 0.1 n HCl aq (100 ml*3). Then the aqueous phase was combined and alkalized with Na_2_CO_3_ to pH = 9~10 and extracted with EA (100 mL*3). The final organic layers were combined and subsequently washed with brine (100 mL*3), dried over Na_2_SO_4_ and concentrated. 8.1 g compound 5 was acquired finally. The yield for the product is ~60% over these three steps.

^1^H NMR (500 MHz, CDCl_3_) δ 7.44(d, *J* = 7.7 Hz, 2H), 7.27 (d, *J* = 7.7 Hz, 2H), 4.43 (s, 1H), 4.14 (q, *J* = 6.8 Hz, 2H), 3.49 (m, 1H), 3.38 (m, 1H), 1.15 (t, *J* = 6.9 Hz, 3H), 0.24 (s, 9H).

Synthesis of 2-amino-3-(4-(trimethylsilyl) phenyl) propanoic acid (6)

7.9 g of ethyl 2-amino-3-(4-(trimethylsilyl)phenyl)propanoate (5) (30 mmol) was added with THF 30 ml and 2 n NaOH aqueous 30 ml. The mixture was then stirred for overnight at room temperature, followed by adding 300 ml of PE. Then the aqueous phase was added to 600 ml of 0.1 N HCl aq in a drop-wise manner with stirring. A lot of white solid was precipitated from the solution. The product was filtered and dried under vacuum to afford the 2-amino-3-(4-(trimethylsilyl) phenyl) propanoic acid (5.6 g, 78%).

^1^H NMR (500 MHz, D_2_O) δ 7.48 (d, *J* = 6.6 Hz, 2H), 7.19 (d, *J* = 6.6 Hz, 2H), 3.39 (m, 1H), 2.91 (m,1H), 2.72(m, 1H), 0.14 (s, 9H).^13^C NMR (100 MHz, MeOD-d3) δ172.28, 140.70, 136.82, 135.05, 129.88, 55.99, 37.66, −1.13. HRMS (ESI) calculated for [M + H] + C12H20NO2Si: 238.1258, found 238.1256.

### Genetic selection of the mutant TMSiPheRS

The pBK-lib-jw1 library consisting of 2 × 109 independent TyrRS clones was constructed using standard PCR methods. DH10B *E. coli* cells (Thermo, catalog number: EC0113) harboring the pREP(2)/YC plasmid was used as the host strain for positive selection. Cells were transformed with the pBK-lib-jw1 library, recovered in SOC for 1 h, washed twice with glycerol minimal media with leucine (GMML) before plating on GMML-agar plates supplemented with kanamycin, chloramphenicol, tetracycline, and TMS-Phe at 50 g/ml, 60 g/ml, 15 g/ml and 1 mm respectively. Plates were incubated at 37 °C for 60 hours and surviving cells were harvested. Subsequently, the plasmid DNA was extracted and purified by gel electrophoresis. The pBK-lib-jw1 DNA was then transformed into electro-competent cells harboring the negative selection plasmid pLWJ17B3, recovered for 1 h in SOC and then plated on LB-agar plates containing 0.2% arabinose, 50 g/ml ampicillin, and 50 g/ml kanamycin. The plates were then incubated at 37 °C for 8–12 hours, and pBK-lib-jw1 DNA from the surviving clones was extracted as described above. The library underwent another round of positive selection, followed by a negative selection and a final round of positive selection (with chloramphenicol at 70 g/mL). At this stage, 96 individual clones were selected and suspended in 50 L of GMML in a 96-well plate, and then replica-spotted on two sets of GMML plates. One set of GMML-agar plates was supplemented with tetracycline (15 g/mL), kanamycin (50 g/mL), and chloramphenicol at concentrations of 60, 80, 100, and 120 g/mL with 1 mm TMSiPhe. The other set of plates were identical but did not contain TMSiPhe, and the chloramphenicol concentrations used were 0, 20, 40, and 60 g/mL. After 60 h incubation at 37 °C, one clone was found to survive at 100 g/mL chloramphenicol in the presence of 1 mm TMSiPhe, but only at 20 g/mL chloramphenicol in the absence TMSiPhe.

### Purification of TMSiPheRS

TMSiPheRS was purified from BL21 (DE3) *E. coli* cells (Thermo, catalog number: EC0114). The gene encoding the TMSiPheRS was cloned into the pET22b vector and then transformed into BL21(DE3) *E. coli* cells. The large-scale expression cultures were grown to an OD of 0.8. After induction for 4–6 hours at 37 °C with 1 mm isopropyl β-d-1-thiogalactopyranoside (IPTG), cells were pelleted by centrifugation and re-suspended in lysis buffer (50 mm Tris, pH = 8.5, 500 mm NaCl, 10 mm β-mercaptoethanol, 5 mm imidazole). Cells were sonicated and the cell lysate was pelleted by centrifugation. The supernatant was collected and incubated with Ni-NTA agarose beads for 2 hours at 4 °C, filtered, and washed with wash buffer (50 mm Tris, pH = 8.5, 500 mm NaCl, 10 mm β-mercaptoethanol, 20 mm imidazole). The synthetase was eluted with a wash buffer containing 300 mm imidazole in buffer A (25 mm Tris, pH = 8.5, 25 mm NaCl, 10 mm β-mercaptoethanol, 1 mm ethylenediaminetetraacetic acid (EDTA)), purified by anion exchange chromatography (Hitrap Mono Q; GE Healthcare) using a salt gradient from 25 mm to 0.5 m NaCl. TMSiPheRS was purified by Sephadex gel column chromatography (Superdex 200 10/300 GL; GE Healthcare) in a buffer containing 50 mm Tris, pH = 8.5, 500 mm NaCl, 10 mm β-mercaptoethanol and concentrated to 25 mg/mL.

### Preparation crystals for TMSiPhe-incorporated sfGFP

The plasmids encoding sfGFP Y182TMSiPhe in pET22b vector was co-transformed with pEVOL-TMSiPheRS into BL21(DE3) *E. coli* cells. Cells were amplified in LB media supplemented with ampicillin (50 µg/mL) and chloramphenicol (30 µg/mL). Cells were then grown to an OD600 = 0.8 at 37 °C. After induction14 hours at 30 °C with 0.2% l-arabinose, 0.3 mm IPTG and 0.5 mm TMSiPhe, cells were harvested by centrifugation. The cells were lysed by French pressing in buffer containing 50 mm HEPES, pH = 7.5, 500 mm NaCl. The supernatant was collected and incubated with Ni-NTA column for 2 hours at 4 °C, filtered, and washed with wash buffer containing 50 mm HEPES, pH = 7.5, 500 mm NaCl, 20 mm imidazole. The protein was eluted with a wash buffer containing 50 mm HEPES, pH = 7.5, 500 mm NaCl, 250 mm imidazole. sfGFP Y182TMSiPhe was purified by size-exclusion column (Superdex 200 increase 10/300 GL; GE Healthcare) in a buffer containing 20 mm HEPES-Na, pH = 7.5, and concentrated to 20 mg/mL. The crystal of sfGFP Y182TMSiPhe were obtained at 16 °C by the hanging drop vapor diffusion by mixing 1 µL protein sample with equal volume of mother liquor containing 10% polyethylene glycol (PEG) 6000 and 2.0 m sodium chloride. The crystal appeared within one week. Crystals were then flash frozen in liquid nitrogen in 10% PEG 6000, 2.0 m Sodium chloride and 20% glycerol.

### Structure determination of sfGFP Y182TMSiPhe

Diffraction data for sfGFP Y182TMSiPhe were collected at beamline BL19U1 of Shanghai Synchrotron Radiation Facility (SSRF). All data collected were indexed, integrated, and scaled using software of XDS and Aimless, respectively^59,60^. The structure of sfGFP Y182TMSiPhe was solved by molecular replacement using sfGFP-66-HqAla, (PDB code: 4JFG) as a search model by Phaser within PHENIX package. Structural refinement was carried out by Phenix. In the refinement process, the program Coot in the CCP4 program suite was used for the model adjustment, and water finding, whereas ligand restraints were produced using the eBLOW contained in PHENIX software package^61^. The structure models were checked using the PROCHECK^62^.

### Crystallization

Crystals of TMSiPheRS alone were grown at 16 °C using the hanging drop vapor diffusion technique against a mother liquor composed of 22% PEG 1500, 100 mm HEPES (pH = 7.5) and 200 mm l-Proline and 1:1 mixture of concentrated synthetase (25 mg/mL). For TMSiPheRS complex, TMSiPhe (100 μm) was incubated with TMSiPheRS (10 μm) for 2 hours at 25 °C. The complex was concentrated to 20 mg/ml, Crystals were grown in hanging drops containing 1.5 μl of complex solution and 1.5 μl of a well solution composed of 24% PEG 1500, 100 mm HEPES (pH = 7.5) and 200 mm l-proline. The crystal appeared after about 1 week. Crystals were flash frozen in liquid nitrogen after a 30 s soak in 26% PEG 1500, 100 mm HEPES (pH = 7.5) and 200 mm l-proline and 20% glycerol.

### Structure determination

X-ray diffraction data of TMSiPheRS alone and TMSiPheRS complex were collected at beamline BL19U1 SSRF. All data collected were indexed, integrated and scaled using software of XDS and Aimless, respectively^59,60^. The structure of TMSiPheRS alone and TMSiPheRS complex was solved by molecular replacement using F2Y–F2YRS complex (PDB code: 4HJX) as a search model by Phaser within PHENIX package. Structural refinement was carried out by Phenix. In the refinement process, the program Coot in the CCP4 program suite was used for the model adjustment, and water finding, whereas ligand restraints were produced using the eBLOW contained in PHENIX software package^61^. The structure models were checked using the PROCHECK^62^.

### Peptide synthesis

A fully phosphorylated 29 amino-acid carboxy-terminal peptide derived from the human V2 vasopressin receptor (V2Rpp: ^343^ARGRpTPPpSLGPQDEpSCpTpTApSpSpSLAKDTSS^371^) was synthesized from Tufts University Core Facility. And the high-affinity version of Gtα (^340^ILENLKDCGLF^350^, GtαCT-HA) were purchased from China Peptides Co., Ltd. with >95% purity as verified by analytical high-performance liquid chromatography. In the competition assays, the GtαCT and the V2Rpp were used as 200 µm concentration.

### Expression and purification of β-arr1 TMSiPhe mutants

The pEVOL-TMSiPheRS plasmids encoding specific *M. jannaschii* tyrosyl amber suppressor tRNA/tyrosyl-tRNA synthtase mutants were co-transformed into BL21 (DE3) *E. coli* cells (purchased from TransGen Biotech, catalog number: CD601-02) together with the pET22b vector harboring the target β-arr1 mutant. The *E. coli* cells were cultured in LB medium. After the 1 L cell culture reached OD600 = 0.6–0.8, the cells were induced with 300 μm IPTG and 0.2% l-arabinose for 12 h at 25 °C to allow protein expression in presence of 0.5 mm TMSiPhe in the culture medium. The cells were lysed by French pressing in buffer A (50 mm Tris-HCl, pH = 8.0, 150 mm NaCl) and the lysate was batch binding with 300 μl Ni-NTA column (GE Healthcare, USA). After an extensive washing with buffer A, the target protein was eluted using 300 mm imidazole in buffer A. The arrestin contains 6x his tag in the carboxyl terminus, thus enabled discarding the incomplete protein during the purification of this step. These proteins were subsequently purified by size-exclusion column Superdex 75 and the buffer was exchanged to buffer B (50 mm Tris-HCl, pH = 7.5, 150 mm NaCl).

### Expression and purification of clathrin TMSiPhe mutants

The plasmids of clathrin terminal domain (TD) (heavy chain) mutant with the pET22b vector were co-transformed into BL21 (DE3) *E. coli* cells (purchased from TransGen Biotech, catalog number: CD601-02) together with the pEVOL-TMSiPheRS plasmids encoding specific *M. jannaschii* tyrosyl amber suppressor tRNA/tyrosyl-tRNA synthtase mutants. The *E. coli* cells were cultured in LB medium. After the 1 L cell culture reached OD600 = 0.6–0.8, the cells were induced with 300 μm IPTG and 0.2% l-arabinose for 12 h at 25 °C to allow protein expression in presence of 0.5 mm TMSiPhe in the culture medium. The cells were lysed by French pressing in buffer A (50 mm Tris-HCl, pH = 8.0, 150 mm NaCl) and the lysate was batch binding with 300 μl Ni-NTA column (GE Healthcare, USA). After an extensive washing with buffer A, the target protein was eluted using 300 mm imidazole in buffer A. The clathrin contains 6x his tag in the carboxyl terminus, thus enabled discarding the incomplete protein during the purification of this step. These proteins were subsequently purified by size-exclusion column Superdex 75 and the buffer was exchanged to buffer B (20 mm HEPES, pH = 7.5, 150 mm NaCl).

### Expression and purification of β2V2R

FLAG-β2V2R and GRK2-CAAX were co-expressed in baculovirus-infected insect cells (Sf9 cells were purchased from Expression Systems (Cat 94-001 S)) using the Bac-to-Bac baculovirus Expression System and cultured in ESF921 media. Cells were infected at a density of 3 × 10^6^ cells per milliliter and cells were stimulated with ISO (10 μm) and harvested at 64 or 72 h after infection. The cell pellets were stored at −80 °C. Cell membranes were disrupted by thawing frozen cell pellets in 300 ml of hypotonic buffer C (10 mm HEPES, pH = 7.5, 20 mm KCl, and protease inhibitor cocktail) and homogenized using a Dounce homogenizer repeated plunging. The membrane fraction was separated from the lysate via ultracentrifugation (100,000 × *g* speed for 40 min in Ti45 rotor). The pellet was washed 3–4 times with a high osmotic buffer D containing 1.0 m NaCl in the above buffer C, and centrifuge as above. The pellet was subsequently solubilized with 1% n-decyl-β-d-maltopyranoside and (DDM, Anatrace) 0.2%CHS (sigma) in buffer E (50 mm HEPES, pH = 7.5, 1 m NaCl). The solubilized membrane fraction was then purified by flag-M1 resin (sigma) affinity chromatography in buffer F (20 mm HEPES, pH = 7.5, 150 mm NaCl, 0.1% DDM, 0.02% CHS). Finally, the sample buffer was exchanged to buffer G (20 mm HEPES, pH = 7.5, 150 mm NaCl, 0.01% LMNG, 0.002% CHS) using a PD-10 desalting column. Purified protein samples were used fresh in the experiments.

### Superdex exclusion chromatography

The purified ppβ2V2R (30 μm) were stimulated with different ligands (60 μm) and then incubated with β-arr1 H295TMSiPhe (10 μm) for 30 min at 25 °C. Then Fab30 (20 μm) was then added to the mixture and the complex was allowed to form for 1 h at 25 °C. The ligand/ppβ2V2R/β-arr1 H295TMSiPhe-Fab30 complex were concentrated and then purified by Superdex 200 increase in 20 mm HEPES, pH = 7.5, 150 mm NaCl, 0.01% LMNG, 0.002% CHS and corresponding ligand (60 μm). The yield of the purified complexes were ~50%, and the purities were judged by size-exclusion chromatography and the electrophoresis.

### NMR experiment

β-arr1 TMSiPhe mutants prepared for NMR analysis were quantified with BCA protein assay kit and diluted with buffer B (containing 10% D_2_O) to 5~20 μm. All 1D ^1^H NMR spectra were recorded with typical total experimental times 8~15 min at 25 °C, on an Avance 950 MHz spectrometer with cryoprobe (Bruker, Billerica, MA). The cryoprobe is proton-optimized Triple (TCI) Resonance cryoprobe (1H, 13 C, 15 N). The spectra were processed and analyzed with the program ZGGPW5 (NS = 256; DS = 4; SW = 20 ppm; AQ = 1.93 s; D1 = 1 s. The number of scans was adjusted to the relative protein concentration in each experiment. The chemical shift of the signal peak was determined by reference to D_2_O (4.68 ppm).

Binding of the V2Rpp to the β-arrestin1 was assessed using β-arrestin1 F388 TMSiPhe (20 μm), in the presence of V2Rpp at a gradient increased concentration, in 50 mm Tris-HCl, pH = 7.5, 150 mm NaCl, 10% D_2_O buffer on a Bruker 950 MHz NMR spectrometer. The signal was normalized with Tris and integrated at shift −0.05 ppm after auto baseline correction by MestReNova.9. Through calculating the ratio of the area of remaining Apo NMR peak and the original concentration of each component, the complex state (Bound) concentration and free ligand (V2Rpp) concentration were obtained for Scatchard plotting and one-site-specific curve fitting.

Buffer for complex of ppβ2V2R/β-arrestin1/Fab30 1D ^1^H NMR spectra was 20 mm HEPES, 150 mM NaCl, 0.01% LMNG, 0.002% CHS, 10% D_2_O, pH = 7.5, 60 μm ligand or control vehicle (diluted DMSO). The total recording time for each experiment was 40 min. spectra were recorded using a Bruker 950 MHz NMR spectrometer at 25 °C.

For the Fab30 titration experiment, 1D ^1^H NMR spectra of the 10 μm β-arr1 H295TMSiPhe with or without 20 μm V2Rpp were recorded by using Fab30 titration at 0 μm, 4 μm, 8 μm, 16 μm, 32 μm, respectively, in 50 mm Tris-HCl, pH = 7.5, 150 mm NaCl, 10% D_2_O buffer on a Bruker 950 MHz NMR spectrometer at 25 °C.

For the receptor titration experiment, 1D ^1^H NMR spectra of the 10 μm β-arr1 H295TMSiPhe with 16 μm Fab30 were recorded by using ppβ2V2R stimulated by ISO titration at 0 μm, 2 μm, 4 μm, 8 μm, 16 μm, 32 μm, respectively, in 20 mm HEPES, 150 mm NaCl, 0.01% LMNG, 0.002% CHS, 10% D_2_O, pH = 7.5 buffer on a Bruker 950 MHz NMR spectrometer at 25 °C.

For monitoring the conformation changes of clathrin experiment, 1D ^1^H NMR spectra of clathrin mutations incubation with ISO-ppβ2V2R/β-arr1 WT complex were recorded in 20 mm HEPES, 150 mm NaCl, 0.01% LMNG, 0.002% CHS, 10% D_2_O, pH = 7.5 buffer on a Bruker 950 MHz NMR spectrometer at 25 °C. And the spectra of clathrin mutations alone were recorded in buffer including 20 mm HEPES, pH = 7.5, 150 mM NaCl, 10% D_2_O on a Bruker 950 MHz NMR spectrometer at 25 °C.

### Expression and purification of Fab30

The purification of Fab30 was performed in BL21 (DE3) *E. Coli* cells (Thermo, catalog number: EC0114) and the competent cells was transformed with the plasmid containing the gene for the 6x his tagged heavy and light chains of Fab30 cloned in the pETDuet-1 vector and was cultured in the LB medium cultures in 2.8 L non-baffled flasks^46^. Cells were then grown to an OD600 = 0.8 at 37 °C and induced with 500 μm IPTG at 18 °C for 16 h. These cells were harvested and frozen with liquid nitrogen, then stored at −80 °C. The frozen cell pellets were thawed at room temperature and lysed in buffer A (20 mm Tris-HCl, pH = 8.0, 150 mm NaCl). The solution was poured into 250 mL centrifuge bottles and spin in SLA 1500 rotor for 30 minutes at 20,000 × *g*. All remaining purification steps were carried out in cold room. The supernatant of the cell lysate were incubated with Ni-NTA beads by 2–12 hours with a ratio of 500 μl beads/1 liter culture. The beads were packed in a column and washed with 40 CV of cold buffer B (20 mm Tris-HCl, pH = 7.55, 150 mm NaCl), and then eluted with buffer C (20 mm Tris-HCl, pH = 7.55, 150 mm NaCl, 250 mm imidazole). Dialyzed with 20 mm Tris-HCl, pH = 7.55, 100 mm NaCl (buffer D) overnight and flash frozen with 10% glycerol.

### GST pull down assay

In total, 0.1 μm wild-type or mutant β-arr1 was mixed with 0.5 μm phospho-receptor C-tail fragment (V2Rpp) and incubated in binding buffer (20 mm Tris-HCl, pH = 7.5, 150 mm NaCl, 2 mm EDTA, 1 mm DTT) at 25 °C for 30 min. In all, 1 μm GST-Clathrin was then added and incubated for another hour. Subsequently, 10 μl GST beads were added into the mixture and the mixture was rolled at 4 °C for 2 h. The GST beads were collected by centrifuge and washed with wash buffer (binding buffer with 0.5% Tween20) for four times. After removing the supernatant, the samples were re-suspended in 50 μl 2 × SDS loading buffer and boiled for 10 min before western blot. Uncropped and unprocessed scans of the blots were provided in Supplementary Fig. 28 and Source Data file.

### Bimane labeling of purified receptors and TRIQ experiment

The method was carried out according to a previously published manuscript^63^. Purified receptors (β2AR-Δ5-Cys271 + Trp135) and mBBr (Invitrogen) were mixed at the same molarity in LMNG buffer (not containing CHS) and incubated overnight on ice in the dark. Fluorophore-labeled receptors were obtained by gel filtration on a desalting column equilibrated with LMNG/CHS buffer (20 mm HEPES, 150 mm NaCl, 0.01% LMNG, 0.002% CHS, pH = 7.5).Fluorescence spectroscopy was measured on a Varioskan flash (Thermo Scientific) instrument with full wavelength scanning mode at 25 °C. In all, 100 μl samples containing 0.2 μm bimane labeled β2AR in a MicroFluor 96-well plate were excited at 390 nm, and the emission fluorescence was measured by scanning from 430 to 500 nm using a 2 nm step. Each data point was integrated for 0.2 s. If the ligand was present, concentration of ligand was set for 5 μm and the incubation time was set for 15 min. We corrected fluorescence intensity for background fluorescence from buffer. Spectra were analyzed using the GraphPad Prism 5.

### BRET assay

HEK293 cells (obtained from Cell Resource Center of Shanghai Institute for Biological Sciences (Chinese Academy of Sciences, Shanghai, China)) seeded in six-well plates were transfected with 0.5 μg BRET donor Flag-β2V2R-Rluc and 1 μg BRET acceptor Lyn-YFP using polyethylenimine. After 24 h of transfection, the cells were detached and distributed into 96-well plates at a density of ~25,000 cells per well. After another 24 h incubation at 37 °C, the cells were washed twice with Tyrode’s buffer (140 mm NaCl, 2.7 mm KCl, 1 mm CaCl_2_, 12 mm NaHCO_3_, 5.6 mm d-glucose, 0.5 mm MgCl_2_, 0.37 mm NaH_2_PO_4_, and 25 mm HEPES, pH = 7.4) and stimulated with vehicle or different ligands (final concentration of 10 μm) at 37 °C for 20 min. Luciferase substrate coelenterazine-h was added at a final concentration of 5 μm before light emissions were recorded using a Mithras LB940 microplate reader (Berthold Technologies) equipped with BRET filter sets. The BRET signal was determined by calculating the ratio of the light intensity emitted by YFP (530/20 nm) over the light intensity emitted by Rluc (485/20 nm).

### Intra-gel digestion and LC-MS/MS analysis and database search

The TMSiPhe-incorporated β-arrestin1 was purified and subjected to the electrophoresis. After decolorized, DTT reduction and alkylated by iodoacetamide, the dyeing strip was digested by trypsin overnight. The peptides were extracted with 60% acetonitrile. The peptide mixture obtained after enzymatic hydrolysis was analyzed by a liquid chromatography-linear ion trap-orbitrap (nanoLC-LTQ-Orbitrap XL, Thermo, San Jose, CA) mass spectrometer. The chromatographic column was a C18 reverse phase column. Mobile phase A: 0.1% FA/H_2_O, B: 0.1% FA/80%CAN/20% H_2_O, flow rate 300 nL/min. A gradient of 90 min was used. Data analysis was performed using Proteome Discoverer (version 1.4.0.288, Thermo Fischer Scientific) software. The MS2 spectrum uses the SEQUEST search engine to search for arrestin H295TMSiPhe containing fasta. The search parameters are: trypsin enzymatic hydrolysis, half cut, two missed cut sites, precursor ion mass error <20 ppm, and fragment ion mass error less than 0.6 Da. The alkylation of cysteine was set as a fixed modification, and the oxidation of methionine and the specific modification of histidine (H + 82.049 Da) were variable modifications. The retrieved peptides and spectral matches (PSM) were filtered using the Percolator algorithm with a *q* value of <1% (1% false discovery rate). The retrieved peptides are combined into a protein under strict maximum parsimony principles.

### Q-TOF mass spectrometry spectrum analysis and database search

LC-MS analysis was performed using a Agilent Q-TOF mass spectrometer in line with a Agilent 1290 HPLC system. The 5 μl purified TMSiPhe-incorporated β-arrestin1 protein was loaded onto a reverse phase column (30 0SB-C8, 2.1 × 50 mm, 3.5 μm particle) (Agilent Technologies, SantaClara, CA). The proteins were then eluted over a gradient: 2% B for 2 min to waste, then turned LC to MS, 2–50% B in 6 min, 50–90% B in 4 min, 90% B sustained for 4 min, then decreased to 2% in 1.1 min, (where B is 100% Acetonitrile, 0.1% formic acid, A is water with 0.1% formic acid) at a flow rate of 0.2 mL/min.and the elution was introduced online into the Q-TOF mass spectrometer (Agilent Technologies, SantaClara, CA) using electrospray ionization. MS data were analyzed by MassHunter biocomfirm software.

### Statistical analysis

For all experiment, the number of replicates and *P* value cutoff are described in the respective figure legends. Error bars are shown for all data points with replicates as a measure of variation with the group. Statistical differences were determined by one-way analysis of variance using the analysis software GraphPad Prism (*P < 0.05; **P < 0.01; ***P < 0.001)

### Reporting summary

Further information on research design is available in the Nature Research Reporting Summary linked to this article.

## Supplementary information

Supplementary Information

Peer Review file

cdx for Figure 1b-TMSiPhe

cdx for Figure 1b-TMSiM-Tyr

cdx for Figure 1b-TMSiM-hCys

cdx for Figure 1b-TMSiM-dcTyr

cdx for Figure 1b-TMSiM-Cys

cdx for Figure 4d-Salmeterol

cdx for Figure 4d-Isoproterenol

cdx for Figure 4d-ICI-118551

cdx for Figure 4d-Clenbuterol

cdx for Figure 4d-Alprenolol

cdx for Figure 4d-BI-167107

Reporting Summary

## Data Availability

Data supporting the findings of this manuscript are available from the corresponding authors upon reasonable request. A reporting summary for this Article is available as a Supplementary Information file. The sfGFP Y182TMSiPhe, TMSiPheRS alone, and TMSiPheRS-TMSiPhe complex crystal structures and associated diffraction data have been deposited in the Protein Data Bank with the accession codes PDB 6KRG, PDB 7CKH, PDB 7CKG. Source data are provided with this paper.

## Source data

Source Data
