# Supplementary material for: DeSiphering receptor core-induced and ligand-dependent conformational changes in arrestin via genetic encoded trimethylsilyl 1H-NMR probe
Source: Nat Commun. 2020 Sep 25;11:4857. doi: 10.1038/s41467-020-18433-5 (PMC7519161; doi:10.1038/s41467-020-18433-5)
Supplement: Supplementary file 1 — Supplementary Information [file 41467_2020_18433_MOESM1_ESM.pdf]

Supplementary Information

DeSiphering receptor core-induced and ligand-dependent conformational changes in arrestin via genetic encoded Trimethylsilyl ^{1}H-NMR Probe

Qi Liu[1, 2, 15], Qing-tao He[1, 3, 4, 15], Xiaoxuan Lyu[1, 15], Fan Yang[2, 4, 15], Zhong-liang Zhu[5, 15], Peng Xiao[3, 4], Zhao Yang[2, 4], Feng Zhang[1], Zhao-ya Yang[1, 3], Xiao-yan Wang[1], Peng Sun[6], Qian-wen Wang[6], Chang-xiu Qu[3, 4], Zheng Gong[3], Jing-yu Lin[2], Zhen Xu[1], Shao-le Song[1], Shen-ming Huang[4], Sheng-chao Guo[3, 4], Ming-jie Han[1, 7], Kong-kai Zhu[8], Xin Chen[9], Alem W. Kahsai[10], Kun-Hong Xiao[11], Wei Kong[4], Fa-hui Li[1], Ke Ruan[12], Zi-jian Li[4], Xiao Yu[2], Xiao-gang Niu[13], Chang-wen Jin[13], Jiangyun Wang[1, 14, 16], Jin-peng Sun[3, 4, 16]

[1]Institute of Biophysics, Chinese Academy of Sciences, 15 Datun Road, Chaoyang district, Beijing, 100101, China.

[2]Key Laboratory Experimental Teratology of the Ministry of Education and Department of Physiology, School of Basic Medical Sciences, Cheeloo college of Medicine, Shandong University, 44 Wenhua Xi Road, Jinan, Shandong, 250012, China.

[3]Key Laboratory Experimental Teratology of the Ministry of Education and Department of Biochemistry and Molecular Biology, School of Basic Medical Sciences, Cheeloo college of Medicine, Shandong University, 44 Wenhua Xi Road, Jinan, Shandong, 250012, China.

[4]Key Laboratory of Molecular Cardiovascular Science, Ministry of Education, Peking University, 15 Xueyuan Road, Haidian District, Beijing, 100191, China.

[5]School of Life Sciences, University of Science and Technology of China, 96 Jinzhai Road, Hefei, Anhui 230026, China.

[6]Wuhan Institute of Physics and Mathematics, Chinese Academy of Sciences, 30 Xiaohongshan Road, Wuchang District, Wuhan, Hubei, 430071, China.

[7]Tianjin Institute of Industrial Biotechnology, Chinese Academy of Sciences, 32 Xiqi Road, Airport Economic Zone, Dongli District, Tianjin, 300308, China.

[8]School of Biological Science and Technology, University of Jinan, 336 Nanxinzhuangxi Road, Shizhong District, Jinan 250022, China.

[9]Department of Medicinal Chemistry, School of Pharmaceutical Engineering and Life Science, Changzhou University, Changzhou, Jiangsu, 213164, China.

[10]Duke University, School of Medicine, Durham, North Carolina 27705, USA.

[11]Department of Pharmacology and Chemical Biology, School of Medicine, University of Pittsburgh, Pittsburgh, Pennsylvania 15261, USA.

[12]Hefei National Laboratory for Physical Science at the Microscale, University of Science and Technology of

China, 443 Huangshan Road, Hefei, Anhui 230027, China.

[13]Beijing Nuclear Magnetic Resonance Center, College of Chemistry and Molecular Engineering, School of Life Sciences, Peking University, Beijing 100084, China.

[14]College of Life Sciences and School of Future Technology, University of Chinese Academy of Sciences, Beijing 100049, China.

[15]These authors contributed equally: Qi Liu, Qing-tao He, Xiaoxuan Lyu, Fan Yang, Zhong-liang Zhu.

[16]Correspondence and requests for materials should be addressed to J.-P.S. (email: sunjinpeng@sdu.edu.cn) or to J.Y.W. (email: jwang@ibp.ac.cn).

Supplementary Fig. 1

a

Substituent group name	Substituent group structure	Methyl hydrogen chemical shift (ppm)
silyl	CH_3Si	0.0 ~ 0.55
alkyl	CH_3C	0.7 ~ 1.82
alkenyl	$CH_3C=$	1.55 ~ 2.68
Phenyl	CH_3	2.12 ~ 2.72
alkyne	$CH_3C\equiv$	1.8 ~ 2.08
ether	CH_3O-	3.25 ~ 4.0
amine	CH_3N	2.10 ~ 3.05
thioether	CH_3S-	2.02 ~ 2.55
halogen	CH_3X	2.15 ~ 4.3

b

residue	αH	βH	CH_3	Other aliphatic *CH*
Gly	3.97			
Ala	4.35	1.39		
Val	4.18	2.13	0.97, 0.94	
Ile	4.23	1.90	0.95, 0.89	γCH_2 1.48,1.19
Leu	4.38	1.65, 1.65	0.94,0.90	γH 1.64
Pro (trans)	4.44	2.28, 2.02		γ CH_2 2.03 ,2.03<br>δ CH_2 3.68 ,3.65
Ser	4.50	3.88, 3.88		
Thr	4.35	4.22	1.23	
Asp	4.76	2.84, 2.75		
Glu	4.29	2.09, 1.97		γ CH2 2.31, 2.28
Lys	4.36	1.85, 1.76		γ CH_2 1.45,1.45<br>δ CH_2 1.70, 1.70<br>ε CH_2 3.02, 3.02
Arg	4.38	1.89, 1.79		γ CH_2 1.70,1.70<br>δ CH_2 3.32,3.32
Asn	4.75	2.83, 2.75		
Gln	4.37	2.13, 2.01		γ CH_2 2.38, 2.38
Met	4.52	2.15, 2.01	2.13	γ CH_2 2.64, 2.64
Cys	4.69	3.28, 2.96		
Trp	4.70	3.32, 3.19		
Phe	4.66	3.22, 2.99		
Tyr	4.60	3.13, 2.92		
His	4.63	3.26, 3.20		

Supplementary Fig. 1. Chemical shift charts of proton NMR.

a. Nukada Kenkichi Methyl chemical shift chart[1]. According to the difference of adjacent groups, the approximate chemical shift ranges of methyl groups were different. In the original data, the graphs were made in the condition that set the chemical shift of hydrogen atoms in water to zero. It had been amended according to the current practice with tetramethylsilane as reference.

b. Random coil ^{1}H chemical shifts for the 20 common amino acid residues[6]. Bundi and Wuthrich obtained the data from NMR measurements in aqueous solution of 20 tetrapeptides H-Gly-Gly-Xxx-Ala-OH, in which Xxx representative a different common amino acid.

Supplementary Fig. 2

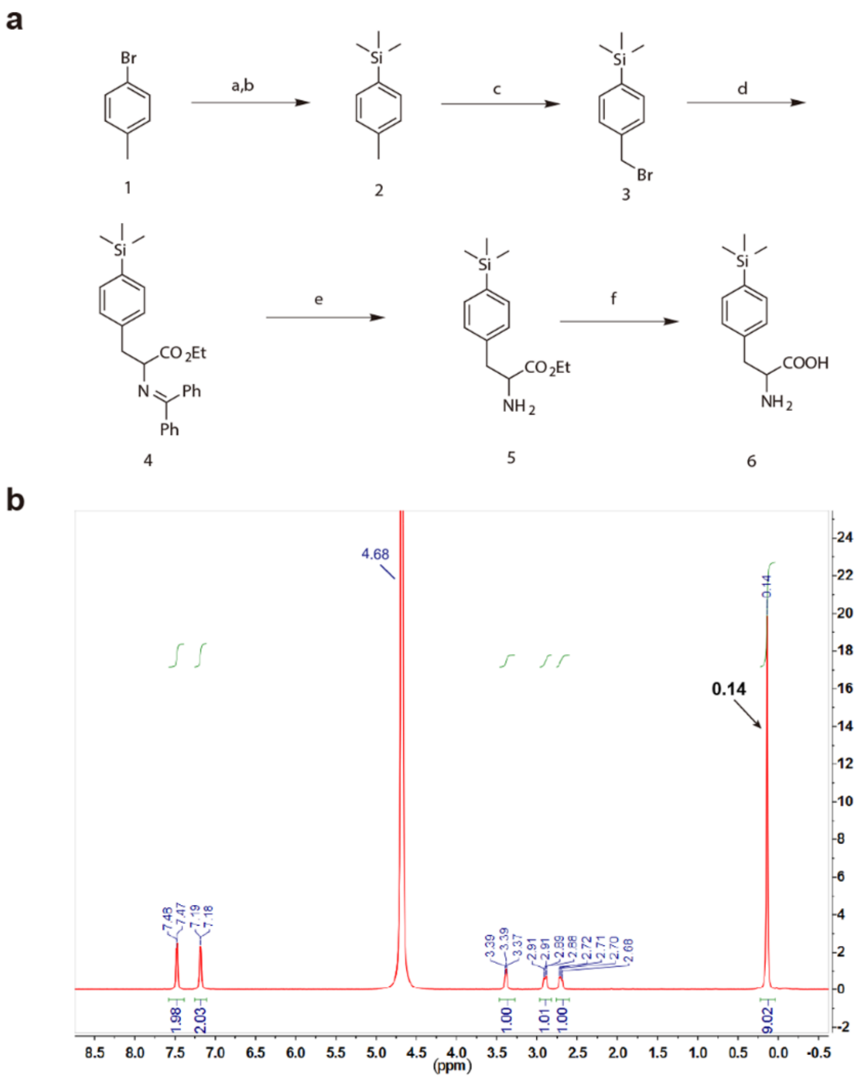

Supplementary Fig. 2. Synthesis of TMSiPhe.

a. The synthetic route of TMSiPhe. Reagents and conditions: a) Mg, THF, I_2, reflux, 4h; b) chlorotrimethylsilane, reflux, 2h; then rt, overnight; yield 87% c) NBS, CCl_4, AIBN, reflux, 4h; d) ethyl N-(diphenylmethylene)glycinate, NaOH, DMSO, 10 °C, 1 h; e) 1N HCl aq, THF, 1h; yield 60%, over 3 steps; f) 2N NaOH aq, THF, rt, overnight; then neutralize with 100 mM HCl aq, yield

78%.

b. 1D ^{1}H NMR of TMSiPhe, (500 MHz, D_2O). The characteristic chemical shift of trimethylsilyl group appeared at 0.14 ppm.

Supplementary Fig. 3

a

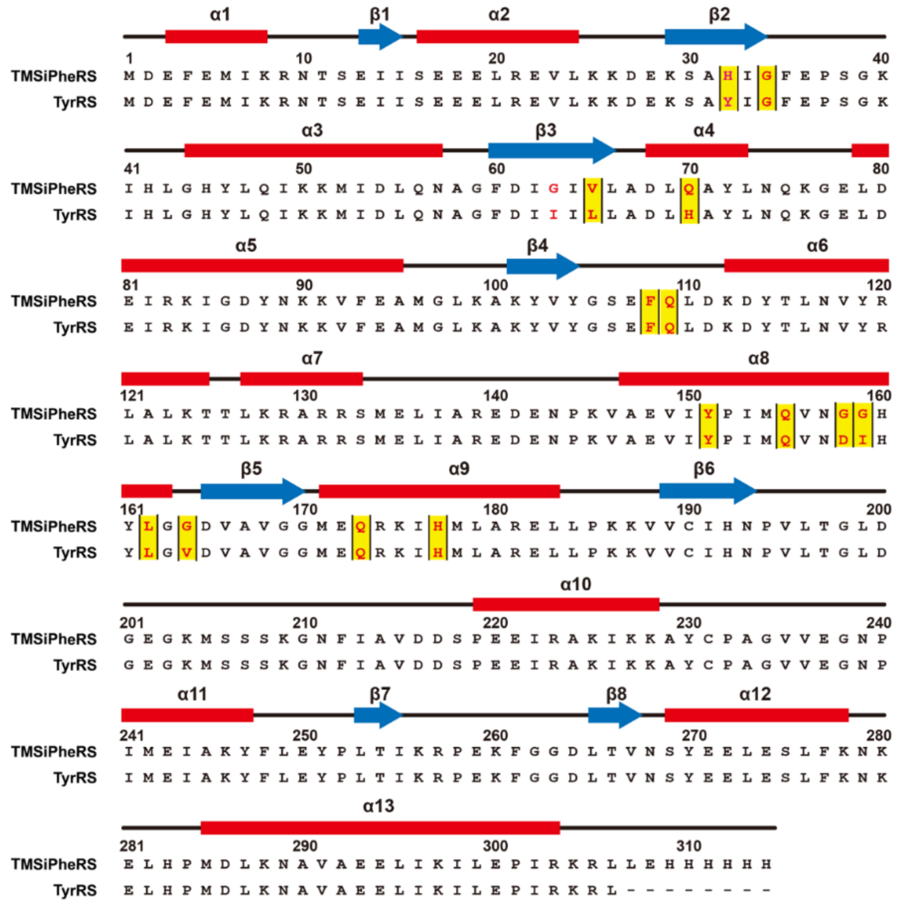

b

	Amino acid (RS)	Distance(Å)	Interaction
TMSiPhe	H32	3.6	Ion dipole interaction
	G34	3.3	hydrophobic
	V65	3.6	hydrophobic
	Q70	4.8	hydrophobic
	F108	5.3	hydrophobic
	Y151	2.8	hydrogen bonds
	Q155	3.3	hydrogen bonds
	G158	3.7	hydrophobic
	G159	4.1	hydrophobic
	L162	4.0	hydrophobic
	Q173	3.1	hydrogen bonds
	H177	5.0	Ion dipole interaction

Supplementary Fig. 3. Sequence alignment of TMSiPheRS and its interaction with TMSiPhe

a. Sequence alignment of TMSiPheRS and wildtype TyrRS. The secondary structure β-strands were marked in blue arrows and α-helices were indicated in red boxes. The residues that interact with TMSiPhe were colored in red and highlighted in yellow.

b. Interactions between the TMSiPheRS and the TMSiPhe.

Supplementary Fig. 4

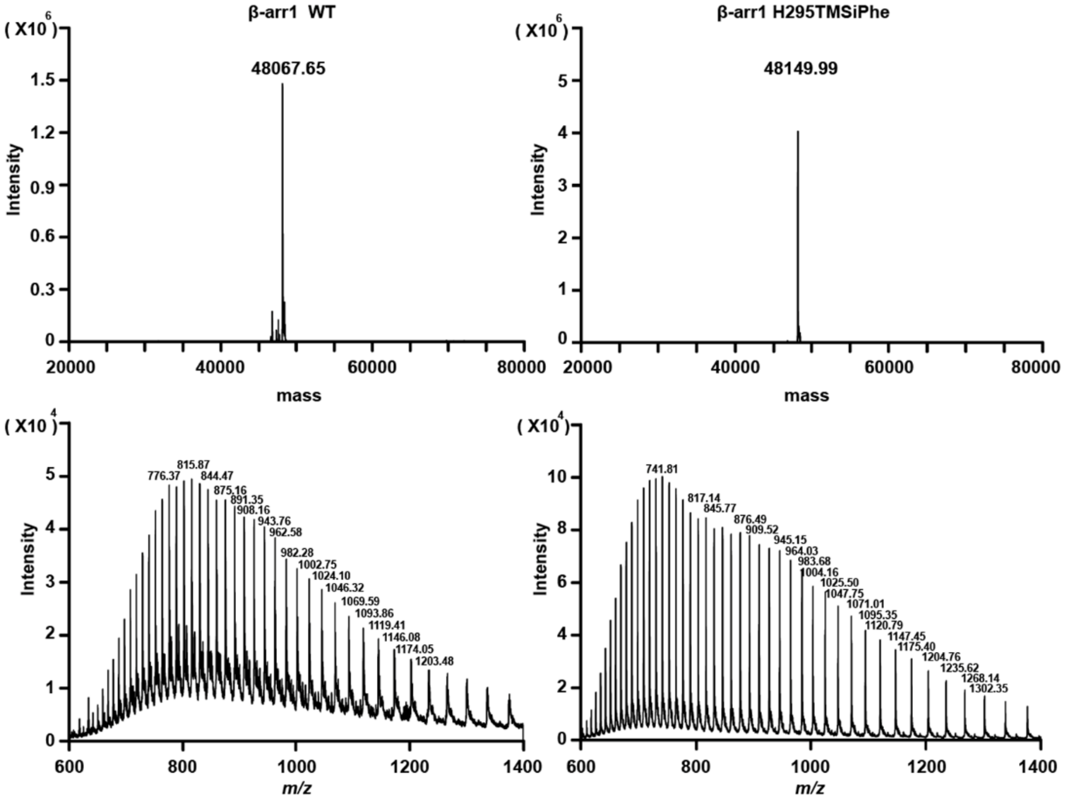

Supplementary Fig. 4. Q-TOF mass spectrometry spectrum of the β-arr1 wild type (left panel) and the β-arr1 H295TMSiPhe (right panel).

Whereas purified β-arr1 wild type shows a main peak representing the molecular weight of 48067, the β-arr1 H295TMSiPhe had a unique peak representing molecular weight of 48149, confirming its effective incorporation of TMSiPhe.

Supplementary Fig. 5

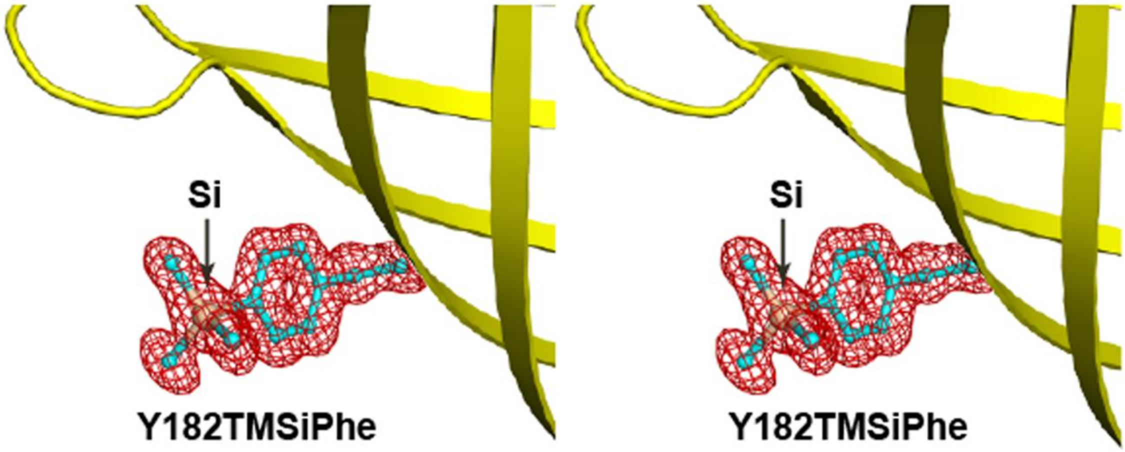

Supplementary Fig. 5. A stereo image and the electron density map of sfGFP Y182TMSiPhe. The 2Fo-Fc annealing omit map of sfGFP-Y182-TMSiPhe clearly shows the electron density of TMSiPhe. The map was contoured at 1.1 σ.

Supplementary Fig. 6

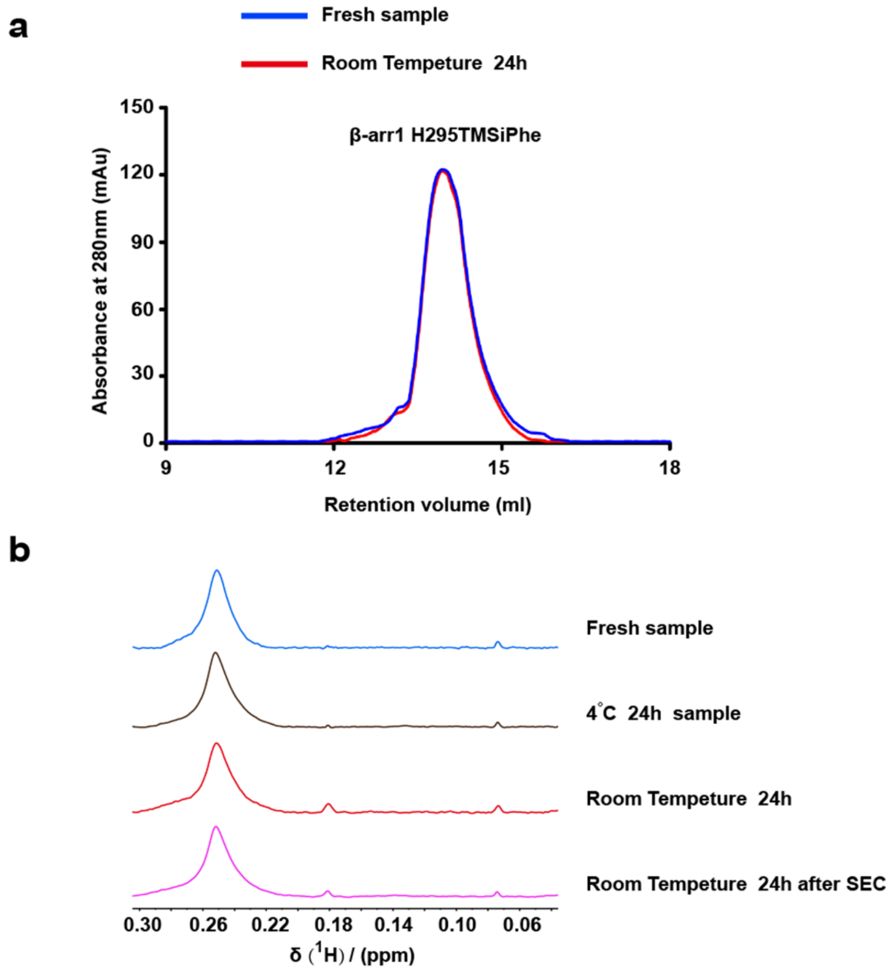

Supplementary Fig. 6. Size exclusion column and corresponding **^{1}H-NMR spectrum of the β-arr1 H295TMSiPhe.**

a. After His-tag affinity purification, the β-arr1 H295TMSiPhe were purified immediately by Superdex 200 increase or had been stored for 24 hours at room temperature (25 °C) before running through Superdex 200 increase.

b. The purified protein was then subject to ^{1}H-NMR spectrum. There was no significant change of the signal at the peak of the 0.26 ppm and 0.07 ppm. The signal of 0.18 ppm may be attributed to conformational change caused by thermodynamic instability.

Supplementary Fig. 7

a

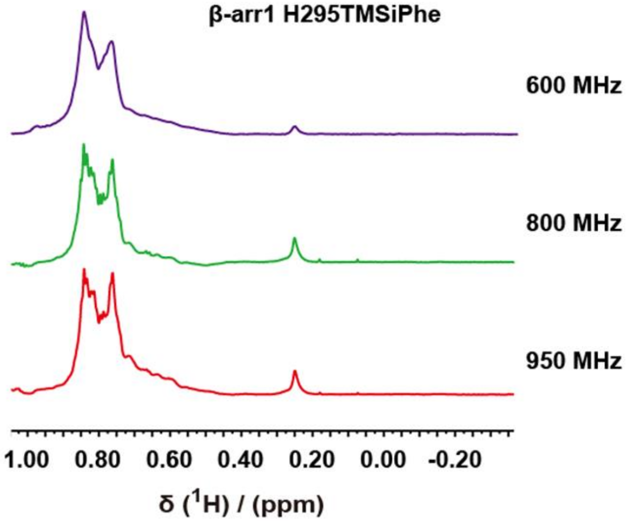

b

	Time (min)	Sample concentration	S/N	Half peak width (ppm)
600 MHz	40	50 μM	74.9	0.0229
800 MHz	40	20 μM	145.9	0.0166
950 MHz	20	5 μM	160.1	0.0165

c

$$R_{2(CSA)} = \frac{1}{T_{2(CSA)}} = \frac{1}{40} \gamma^2 H_0^2 \delta_Z^2 \sum_{i=0}^{2} C_i [3J_i(\omega_0) + 4J_i(0)]$$

$$J_i(\omega) = 2\tau_i / (1 + \omega^2 \tau_i^2)$$

$$\Delta\sigma = \sigma_{\parallel} - \sigma_{\perp} = (3/2)\,\delta_Z$$

Supplementary Fig. 7. NMR measurements of the β-arr1 H295TMSiPhe using different NMR spectrometer.

a. ^{1}H-NMR spectrum of the β-arr1 H295TMSiPhe measured using the 600 MHz, 800 MHz, 950 MHz spectrometer respectively.

b. A summary table of the data collection time, S/N, half peak width and protein concentration used in the supplementary figure 6a.

c. The non- chemical shift anisotropy (CSA) effect: γ: Magnetogyric ratio; H_0: Magnetic field intensity; $\sigma_{\parallel} - \sigma_{\perp}$: Chemical shift anisotropy; J_i: Spectral densities; ω_0: Spectrometer frequency; C_i: Coefficients related to Euler angles and asymmetry; τ_i: Internal rotation correlation time, if it was fixed, an increase in correlation time for isotropic tumbling (τ_c) leads to an increase in relaxation. Since hydrogen atoms only have S orbital electrons, they have the same $\sigma_{\parallel}$ and $\sigma_{\perp}$, so the sensitivity will not be weakened by the CSA effect; however, F atoms have asymmetric P orbital electrons, their $\sigma_{\parallel}$ and $\sigma_{\perp}$are different. Therefore, the CSA effect for F atom widens the signal peak and weakens the signal. Especially when the magnetic field strength or protein molecular weight is increased that corresponding to a larger H_0 or τ_C. Collectively, for a Protein with large molecular weight, the hydrogen atom will be better qualified[2].

Supplementary Fig. 8

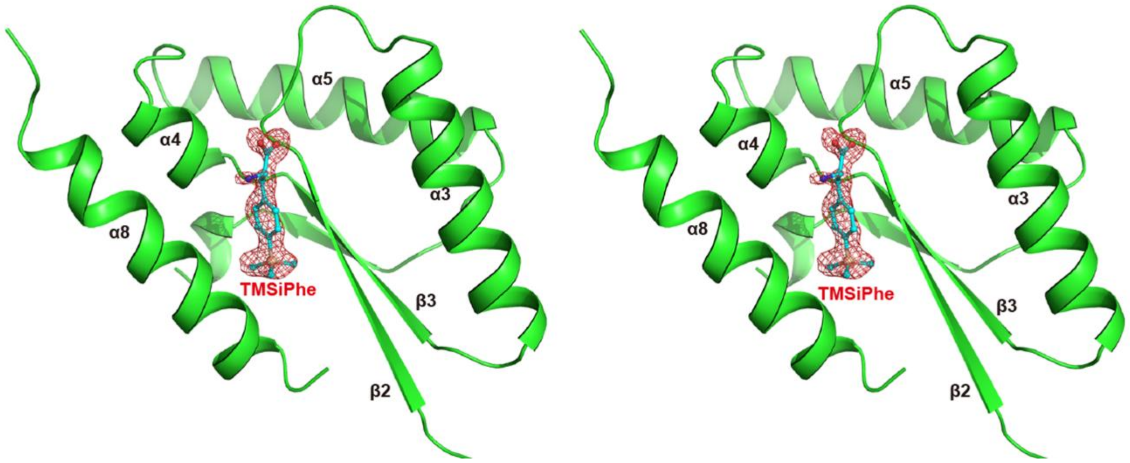

Supplementary Fig. 8. A stereo image and the electron density map of TMSiPhe in TMSiPheRS-TMSiPhe complex.

Binding of TMSiPhe at the active site of TMSiPheRS. The 2Fo-Fc annealing omit electron density map of TMSiPhe was contoured at 1.0 σ.

Supplementary Fig. 9

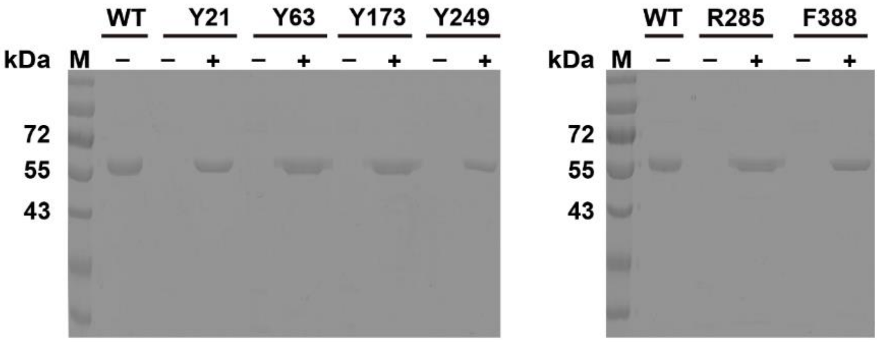

Supplementary Fig. 9. Expression and purification β-arr1 with TMSiPhe incorporated at different positions

The plasmids of β-arr1 mutants and pEVOL-TMSiPheRS plasmids were co-transformed into BL21(DE3) *E. coli* cell. The *E. coli* cells were cultured in 1 L LB medium. After the cell culture reached OD_{600} 0.6-0.8 at 37 ℃, the cells were induced with 300 μM isopropyl-*β*-D-thiogalactopyranoside (IPTG) and 0.2% L-arabinose for 12 h (25 ℃) to allow protein expression in presence or absence of 1 mM TMSiPhe in the culture medium. The cells were lysed by French press in buffer A (50 mM Tris-HCl, pH = 8.0, 150 mM NaCl) and the lysate was batch binding with 300 μL Ni-NTA column (GE Healthcare, USA). After extensive washing with buffer A, the β-arr1 TMSiphe incorporated protein was eluted using 300 mM imidazole in buffer A. These proteins were subsequently purified by size exclusion column Superdex 75 and the buffer was exchanged to buffer B (50 mM Tris-HCl, pH = 7.5, 150 mM NaCl). Coomassie-stained gel was used to analysis the purified β-arr1 mutants with TMSiPhe incorporation.

+: Presence of 1 mM TMSiPhe in the culture medium; -: Absence of 1 mM TMSiPhe in the culture medium.

Supplementary Fig. 10

	Y21 TMSiPhe	Y63 TMSiPhe	Y173 TMSiPhe	Y249 TMSiPhe	R285 TMSiPhe	H295 TMSiPhe	F388 TMSiPhe
Chemical shift (ppm)	-0.245	0.185	-0.247	0.145	0.158	0.251	-0.050

Supplementary Fig. 10. Chemical shifts in the 1D ^1H-NMR spectra assigned to different TMSiPhe incorporation positions in β-arr1.

The NMR spectra of TMSiPhe incorporated β-arr1 at different positions were recorded in a buffer of 50 mM Tris-HCl, pH = 7.5, 150 mM NaCl,10% D_2O at 25 °C, using a Bruker 950 MHz NMR spectrometer.

Supplementary Fig. 11

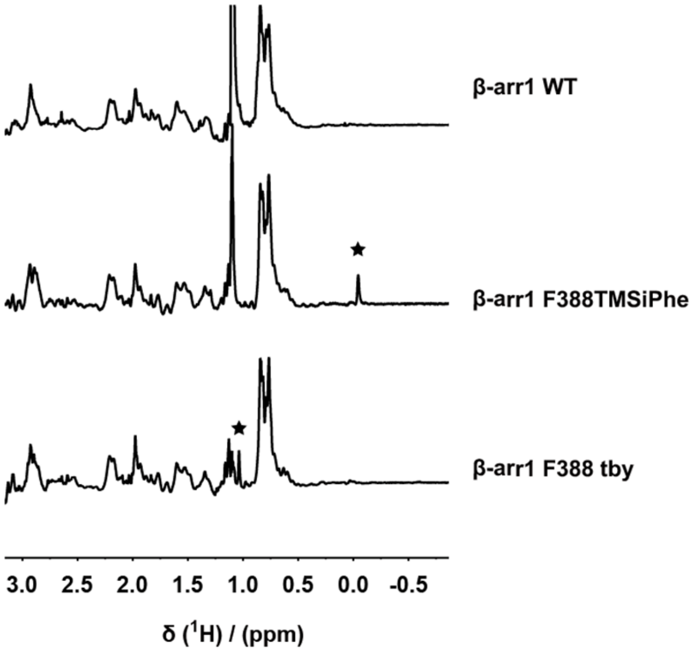

Supplementary Fig. 11. 1D ^1H NMR spectrum for the β-arr1 F388 TMSiPhe mutant was compared with the corresponding O-tert-Butyltyrosine (tby) mutant[3].

The ^1H NMR signal from trimethylsilyl incorporated in β-arr1 was easier to be identified than that from tert-butoxy group. The spectra were recorded in a buffer of 50 mM Tris-HCl, pH = 7.5, 150 mM NaCl, 10% D_2O at 25 °C, using a Bruker 950 MHz NMR spectrometer.

Supplementary Fig. 12：

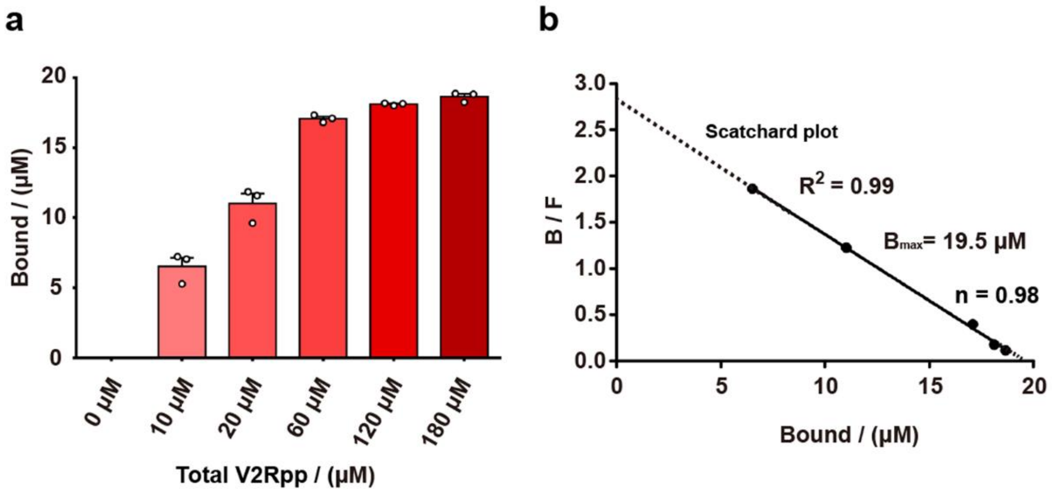

Supplementary Fig. 12. Active state of β-arr1–F388-TMSiPhe in response to titration with V2Rpp.

a. By calculating the ratio between the ^{1}H-NMR signal peaks assigned to the remaining inactive β-arr1 and the original concentration of each component, the concentrations of the β-arr1/V2Rpp complex state (Bound) and free ligand (V2Rpp) were obtained. Bar graph representing the concentration of the complex states at gradient total concentration of V2Rpp. Values are the mean ± SEM of three independent experiments (n=3).

b. The n value (binding stoichiometry, 0.98) was estimated by Scatchard plot analysis.

Supplementary Fig. 13

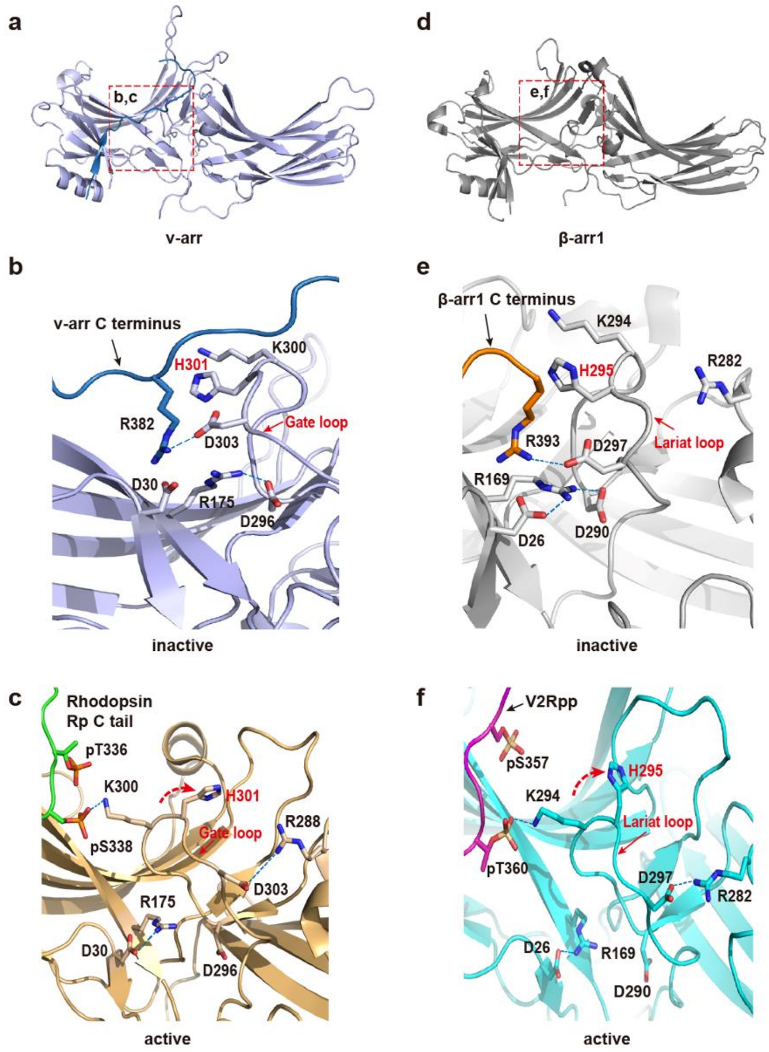

Supplementary Fig. 13. Conformational change of the polar core after arrestin activation

a, d. Overall view of inactive visual-arrestin (v-arr) (PDB code:1CF1) **(a)** and inactive β-arr1 (1G4M) **(d)**, with region of polar core in red box.

b, e. The inactive state of arrestin is stabilized by polar core, which was composed by extensive charge interactions of Asp 296, Asp 303, Arg 175, Arg 382 in visual-arrestin and Asp26, Arg169, Asp290, Asp297, Arg393 in β-arrestin 1, respectively.

c, f. After activation by phospho-rhodopsin **(c)** or V2Rpp **(f)**, the salt bridge Asp297.vs.Arg393 and Asp304.vs.Arg382 within β-arr1 (equivalent of Asp296.vs.Arg175 and the Asp303.vs.Arg382 within visual arrestin respectively) were broken. The TMSiPhe at the H295 position, which is close to both D290 and D297 of the β-arr1, is a suitable probe for monitoring the polar core conformation change without functionally affect arrestin activation.

Supplementary Fig. 14

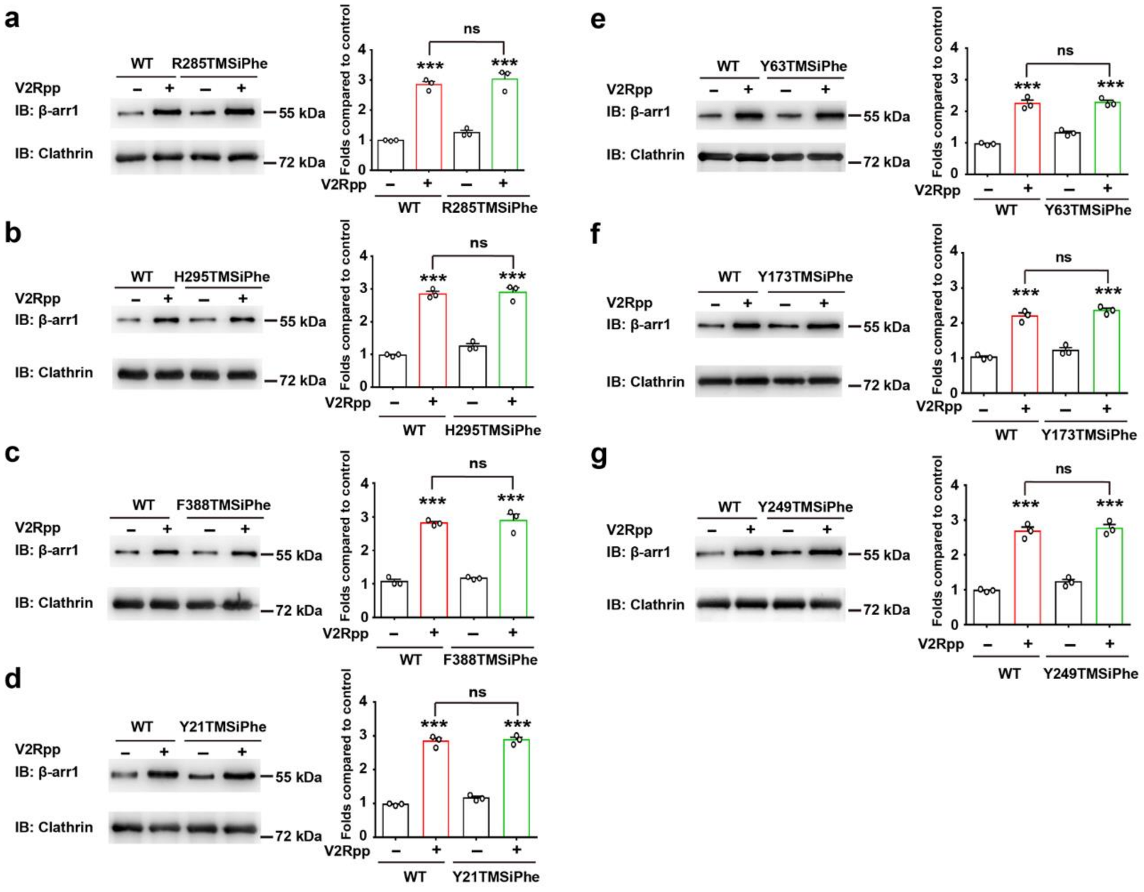

Supplementary Fig. 14. Effects of TMSiPhe mutation of β-arrestin1 on V2Rpp promoted β-arr1/clathrin complex formation.

0.1 μM wild-type, (a) R285TMSiPhe, (b) H295TMSiPhe, (c) F388TMSiPhe, (d) Y21TMSiPhe, (e)Y63TMSiPhe, (f) Y173TMSiPhe, (g) Y249TMSiPhe mutants of β-arr1 was mixed with 0.5 μM V2Rpp in binding buffer (20 mM Tris-HCl, pH = 7.5, 150 mM NaCl) at 25 °C for 30 min respectively. After incubation, 1 μM GST-clathrin was added and then incubated for another 1 h. The complexes were pulled down using GST beads, and the amount of β-arr1 bound to clathrin was determined by using a specific β-arr1 antibody. The results suggest that R285TMSiPhe, H295TMSiPhe, F388TMSiPhe, Y21TMSiPhe, Y63TMSiPhe, Y173TMSiPhe and Y249TMSiPhe exhibited functional integrity in vitro. The western blot signals of β-arr1 bound to clathrin were quantified and shown as columns. Values are the mean ± SEM of three independent experiments (n=3) for the wild type (WT) and mutants. Statistical differences between WT and mutations were determined by one-way ANOVA with Tukey test. ***, $p<0.001$ (V2Rpp stimulation were compared with control vehicles); ns, One-way ANOVA (R285TMSiPhe, H295TMSiPhe, F388TMSiPhe, Y21TMSiPhe, Y63TMSiPhe, Y173TMSiPhe,

Y249TMSiPhe mutations were compared with the wild type). ns, no significant difference. +: with V2Rpp; -: without V2Rpp. Full blots are shown in Supplementary figure 28.

Supplementary Fig. 15

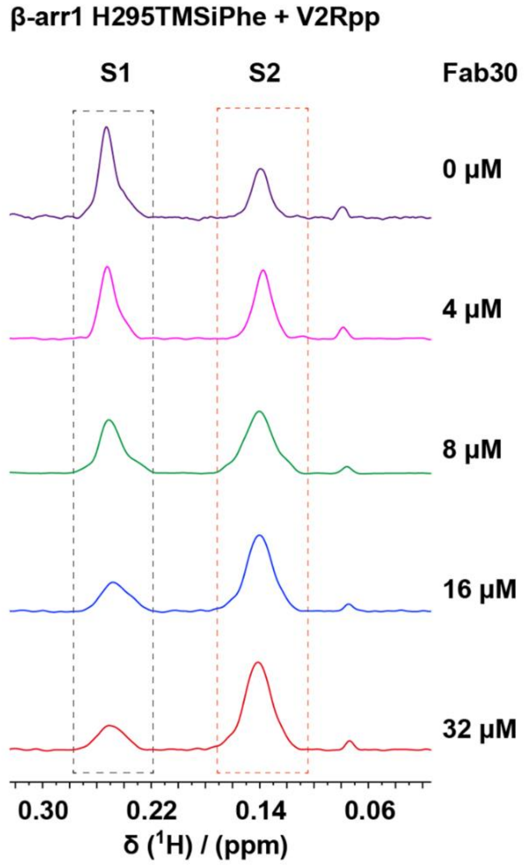

Supplementary Fig. 15. Effects of Fab30 on β-arrestin1 conformational change at H295TMSiPhe site.

1D ^{1}H NMR spectra of β-arr1 H295-TMSiPhe in response to titration with Fab30. With increasing concentrations of Fab30, the peak at 0.25 ppm decreased (representing the S1 state), whereas the peak at 0.15 ppm increased (representing the S2 state). The results indicated that the Fab30 stabilized the active arrestin conformation in a concentration dependent manner.

Supplementary Fig. 16

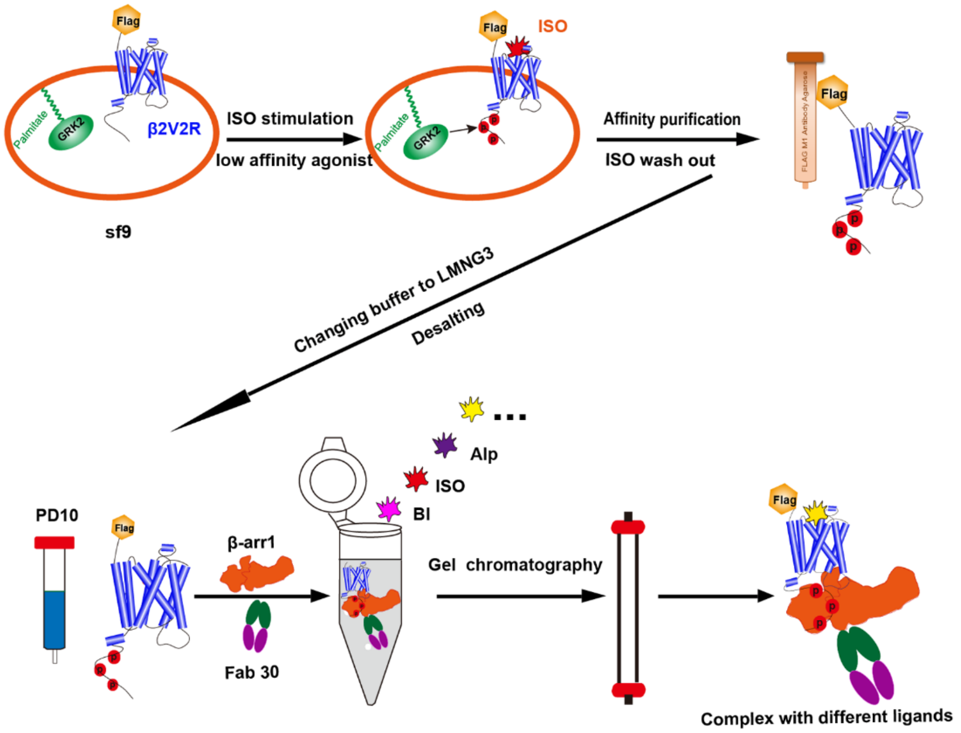

Supplementary Fig. 16. Schematic flowchart for ppβ2V2R/β-arr1 complex formation.

The baculovirus vector harboring β2V2R and GRK2 genes were co-infected in Sf9 cells. After 64~72 hours culture, the cells were stimulated with ISO (low-affinity agonist) for 15 min. The cells were lysed, and cell membrane components were dissolved in buffer containing 1% DDM and 0.2% CHS, purified by M1-Flag beads and desalting column (PD 10) subsequently. ISO was washed off during purification and ligand free phosphorylated β2V2R (ppβ2V2R) was obtain. The ppβ2V2R were then incubated with saturated concentration of various ligands individually, and subsequently combined with β-arr1 H295TMSiPhe and Fab30 to acquire stable complexes by Gel Chromatography. The red circles indicate the phosphorylation.

Supplementary Fig. 17

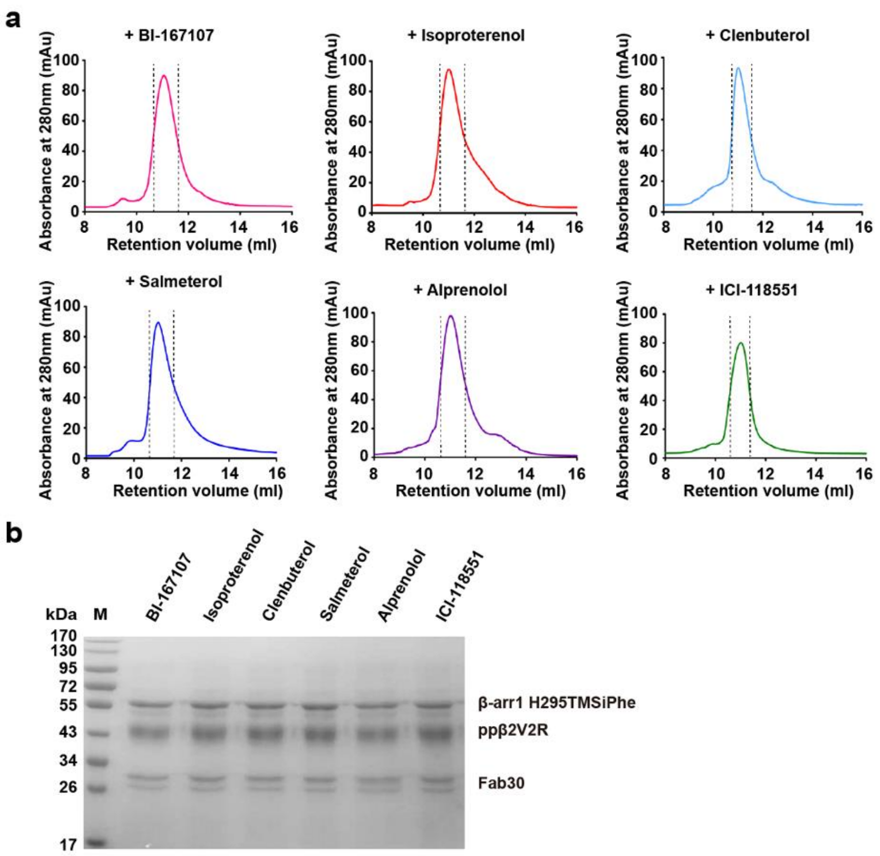

Supplementary Fig. 17. Purity analysis of the complex of ppβ2V2R/β-arr1 H295TMSiPhe/Fab30 with different ligands.

a. ppβ2V2R (30 μM) stimulated with different ligands (60 μM) were incubated with β-arr1 H295TMSiPhe (10 μM) for 30 min at 25 °C. Then Fab30 (20 μM) was added to the mixture and the complex was incubated for 1h at 25 °C. The ligand/ppβ2V2R/β-arr1 H295TMSiPhe-Fab30 complex was purified by Superdex 200 increase in 20 mM HEPES pH = 7.5, 150 mM NaCl, 0.01% LMNG, 0.002% CHS and corresponding ligand (60 μM). Flow rate was 0.5 ml per minute. The yield of the purified complexes were about 50%, and the purities were verified by size exclusion chromatography. **b.** Coomassie-stained gel showed the purified receptor/arrestin complex, samples were collected for the fraction of the half-height width.

Supplementary Fig. 18

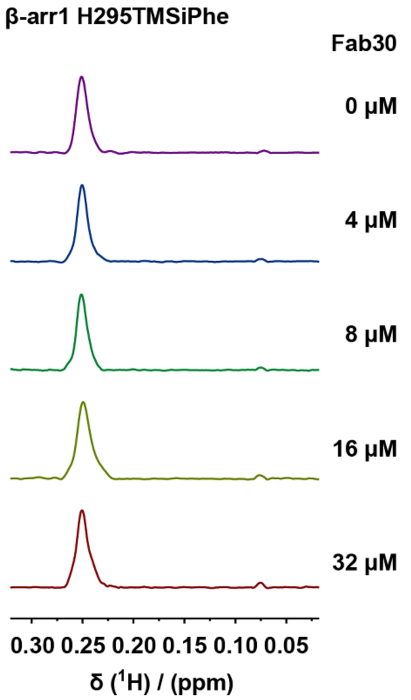

Supplementary Fig. 18. Effect of Fab30 on the NMR spectrum of the β-arr1 H295TMSiPhe. Incubation with an increasing amount of Fab30 has no significant effect on the ^{1}H NMR signal from trimethylsilyl incorporated at the β-arr1 H295TMSiPhe ranging from 0.05 to 0.30 ppm. The spectra were recorded in a buffer of 50 mM Tris-HCl, pH = 7.5, 150 mM NaCl, 10% D_2O at 25 °C, using a Bruker 950 MHz NMR spectrometer.

Supplementary Fig. 19

Sample		S1 (0.26 ppm)			S3 (0.07 ppm)			S3/S1	Δ S3/S1
		Peak areas (x10^3)	Resdiual error	S/N	Peak areas (x10^3)	Resdiual error	S/N		
H295TMSiPhe -Fab30 -ppβ2V2R	---	34.8	37.2	109.8	1.8	2.7	30.4	0.05	0.00
	BI	24.8	29.0	102.5	8.2	55.6	90.8	0.33	0.28
	ISO	23.0	65.5	93.9	5.3	31.6	51.4	0.24	0.19
	Clen	23.2	313.6	33.0	4.0	79.6	33.6	0.17	0.12
	Salm	26.1	398.3	19.9	4.3	96.4	12.8	0.17	0.12
	Alp	33.6	147.0	80.5	2.0	7.7	51.4	0.06	0.01
	ICI	32.7	143.6	42.6	1.4	10.8	14.0	0.04	-0.01

Supplementary Fig. 19. ^{1}H-NMR characteristic signal peak position and area analysis of the complex of ppβ2V2R/β-arr1 H295TMSiPhe/Fab30 in the presence of different ligands.

The peak area for each spectrum was calculated by using the automatic baseline correction and fitting region function of the software MestReNova 9.0.1. The fitting region of the S1 and S3 were 0.2~0.3 ppm and 0.05~0.1 ppm respectively, and then the area of the main peaks and corresponding residual error were obtained. Signal-to-noise ratio was measured using 'sinocal' routine within Topspin 4.0 (Bruker Biospin, Billerica MA), on the TMSiPhe signal of β-arr1-H295TMSiPhe at 0.25 or 0.07 ppm, using a 2 ppm noise regions (centered around 11 ppm) for SNR calculations.

Supplementary Fig. 20

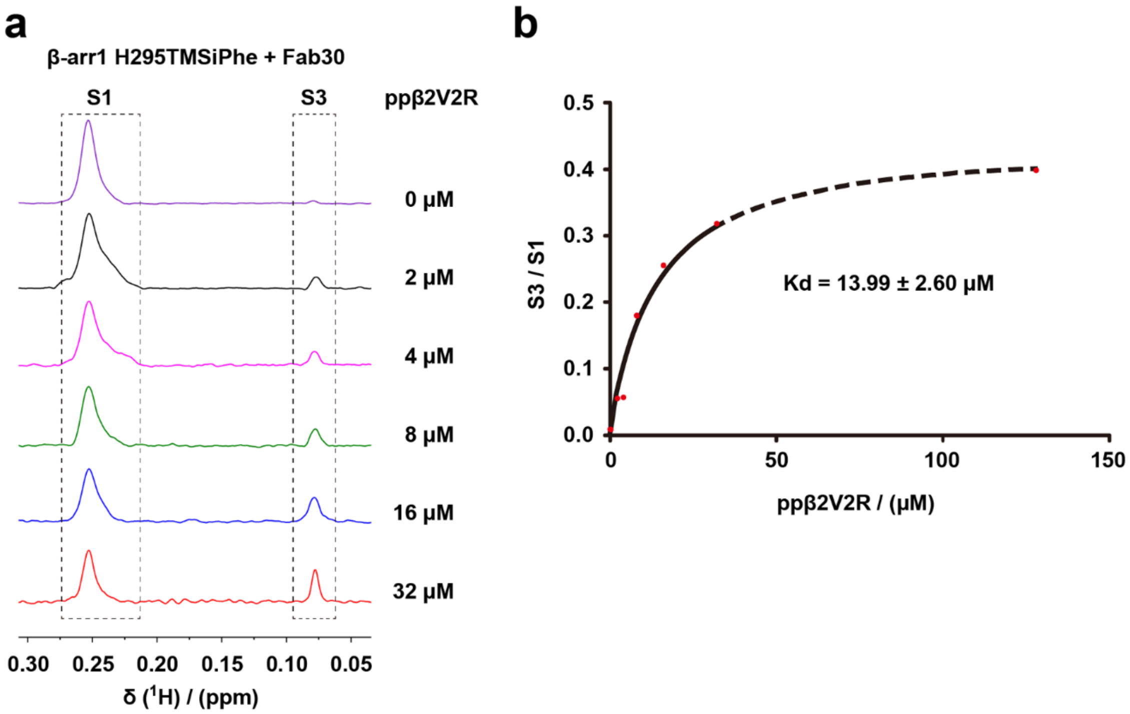

Supplementary Fig. 20. Effects of increasing concentration of ppβ2V2R on β-arrestin1 conformational change at H295TMSiPhe site.

1D ^{1}H NMR spectra of β-arr1 H295-TMSiPhe in response to titration with ppβ2V2R. **a.** With increasing concentrations of ppβ2V2R, the peak at 0.25 ppm decreased (representing the S1 state), whereas the peak at 0.07 ppm increased (representing the S3 state). **b.** Fitting the S3/S1 ratio to the one site binding equation. The Kd is approximately 13.99 μM (13.99±2.60 μM) and the R^2 = 0.981. Notably, the S3:S1 will reach to 2:3 even at the saturated concentration of ppβ2V2R, a concentration probably with most

arrestin bound to the receptor. Therefore, it is very likely that the conformational state of the β-arr1 H295TMSiPhe site was in exchange between inactive and active conformation because of its own flexibility, even with the saturated receptor condition.

Supplementary Fig. 21

a

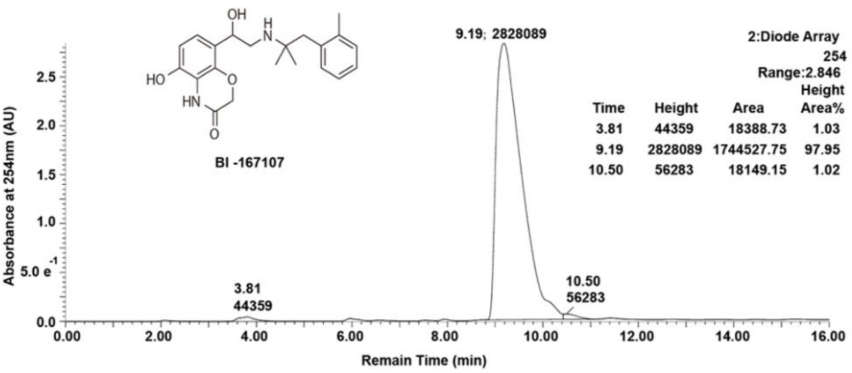
OH
H
N
HO
HN
O
O
BI -167107
9.19; 2828089
2:Diode Array
254
Range:2.846
Time
Height
Area
Height
Area%
3.81
44359
18388.73
1.03
9.19
2828089
1744527.75
97.95
10.50
56283
18149.15
1.02
3.81
44359
10.50
56283
Absorbance at 254nm (AU)
2.5
2.0
1.5
1.0
5.0 e-1
0.0
0.00
2.00
4.00
6.00
8.00
10.00
12.00
14.00
16.00
Remain Time (min)

b

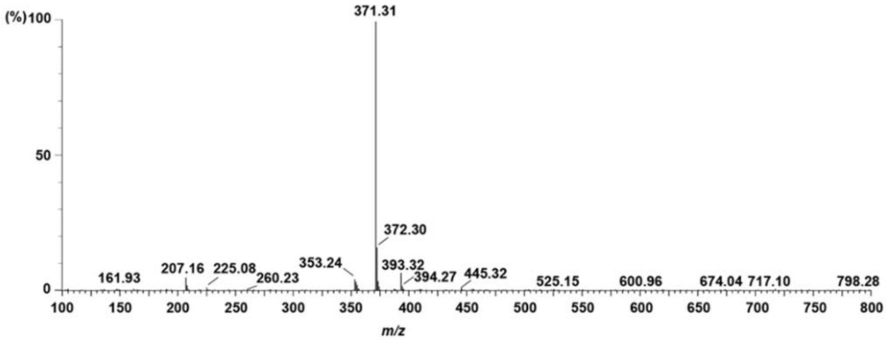
(%)100
50
0
371.31
372.30
161.93
207.16
225.08
260.23
353.24
393.32
394.27
445.32
525.15
600.96
674.04
717.10
798.28
100
150
200
250
300
350
400
450
500
550
600
650
700
750
800
m/z

c

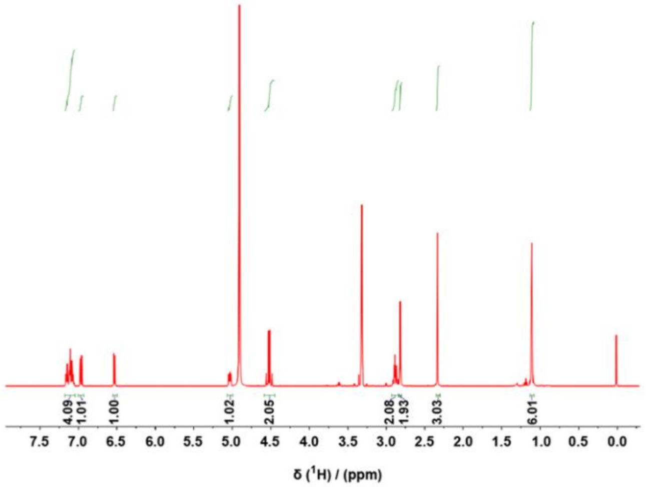
4.09
1.01
1.00
1.02
2.05
2.08
1.93
3.03
6.01
7.5
7.0
6.5
6.0
5.5
5.0
4.5
4.0
3.5
3.0
2.5
2.0
1.5
1.0
0.5
0.0
δ (1H) / (ppm)

d

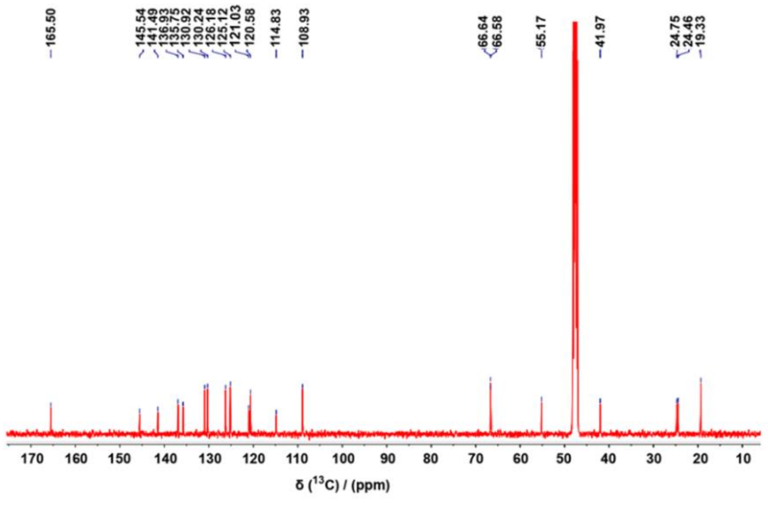
165.50
145.54
141.49
136.93
135.75
130.92
130.24
126.18
125.12
121.03
120.58
114.83
108.93
66.64
66.58
55.17
41.97
24.75
24.46
19.33
170
160
150
140
130
120
110
100
90
80
70
60
50
40
30
20
10
δ (13C) / (ppm)

Supplementary Fig. 21. Quality control of the synthesized BI-167107[7].

a, b. Purity analysis of the compound BI-167107 by HPLC-MS. The content of main products in liquid chromatography was 97.95%, The corresponding ion mass was 371.31, which was consistent with $[M+H]^+$ of BI-167107.

c, d. Identification of the compound BI-167107 by Hydrogen NMR and Carbon NMR .

^{1}H NMR (500 MHz, CD_3OD) δ 7.17 – 7.04 (m, 4H), 6.97 (d, J = 8.5 Hz, 1H), 6.53 (d, J = 8.5 Hz, 1H), 5.03 (dd, J = 8.2, 4.5 Hz, 1H), 4.53 (dt, J = 22.4, 10.1 Hz, 2H), 2.93 – 2.84 (m, 2H), 2.82 (s, 2H), 2.34 (s, 3H), 1.13 – 1.08 (m, 6H).

^{13}C NMR (125 MHz, CD_3OD) δ 165.50, 145.54, 141.49, 136.93, 135.75, 130.92, 130.24, 126.18, 125.12, 121.03, 120.58, 114.83, 108.93, 66.64, 66.58, 55.17, 41.97, 24.75, 24.46, 19.33.

Supplementary Fig. 22

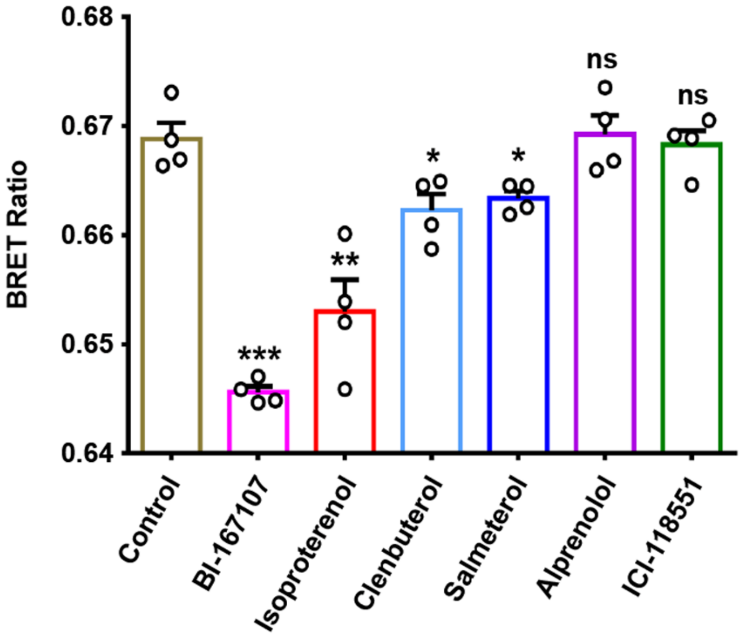

Supplementary Fig. 22. Ligands induced receptor (β2V2R) internalization was measured by BRET assay.

This assay was carried out according to previously published work[4]. Flag-β2V2R-Rluc and Lyn-YFP plasmids were co-transfected into HEK293 cells. 48 h after transfection, the cells were starved and stimulated with vehicle or different ligands at 37 °C for 20 min. The BRET ratio was obtained by calculating the ratio of the light emitted by YFP (530 nm) and the light emitted by Rluc (485 nm). Values are the mean ± SEM of four independent experiments (n=4) for the β2V2R with or without different ligands. Statistical differences between control and β2V2R with different ligands were

determined by one-way ANOVA with Tukey test. ***, p<0.001; **, p<0.01; *, p<0.05, ns: no significant difference.

Supplementary Fig. 23

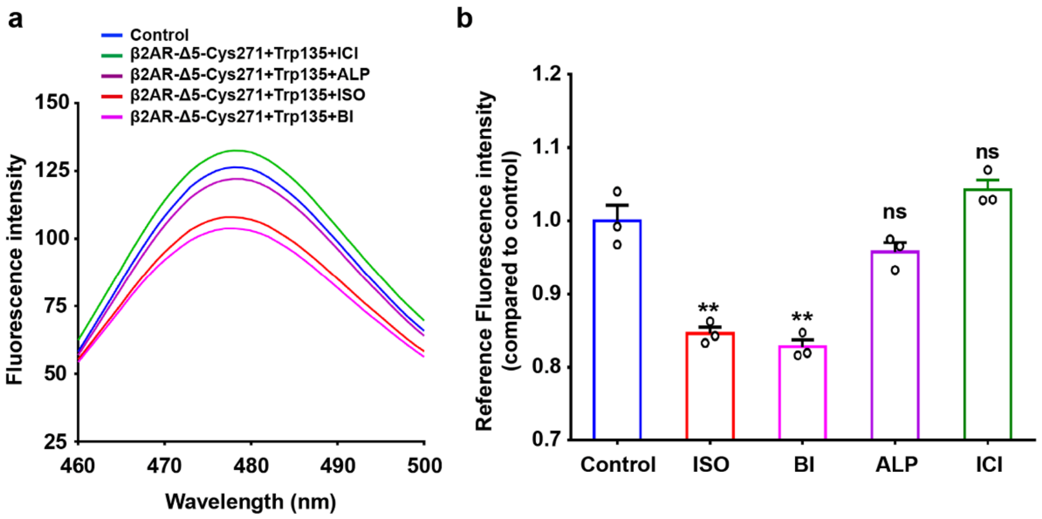

Supplementary Fig. 23. Ligands induced receptor transmembrane core conformational change revealed by fluorescence quenching experiment.

a, b. Fluorescence intensity of the bimane-attached β2V2R-Δ5-Cys271+Trp135 was detected according to previously published work[5]. **a)**. Emission scans of bimane fluorescence before and after stimulation with different ligand, reduction in fluorescence intensity in response to ligands reflected the extent of torsion between transmembrane helices III and VI. The excitation wavelength was 390 nm. The concentration of receptor in the experiments was 200 nM. We corrected fluorescence intensity for background fluorescence from buffer and ligands. **b).** Bar graph represented the relative fluorescence value of ligand incubated receptor compared to the control vehicles. The fluorescence intensity values at the wavelength of maximal emission (478nm). Values are the mean ± SEM of four independent experiments (n=3) for the β2V2R with or without different ligands. Statistical differences between control and β2V2R with different ligands were determined by one-way ANOVA with Tukey test. **, p<0.01; ns: no significant difference.

Supplementary Fig. 24

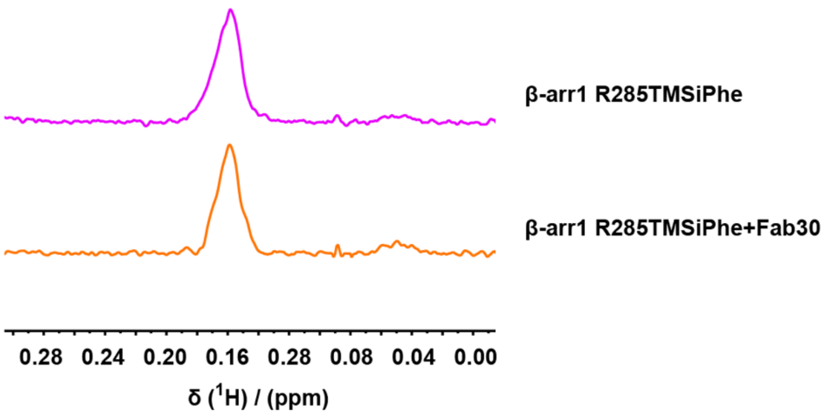

Supplementary Fig. 24. Effect of Fab30 on the NMR spectrum of the β-arr1 R285TMSiPhe. Adding equal amount of Fab30 has no significant effect on the ^{1}H NMR signal from trimethylsilyl-incorporated β-arr1 R285TMSiPhe, the chemical shift of which ranges from 0.00 to 0.30 ppm. The spectra were recorded in a buffer of 50 mM Tris-HCl, pH = 7.5, 150 mM NaCl, 10% D_2O at 25 °C, using a Bruker 950 MHz NMR spectrometer.

Supplementary Fig. 25

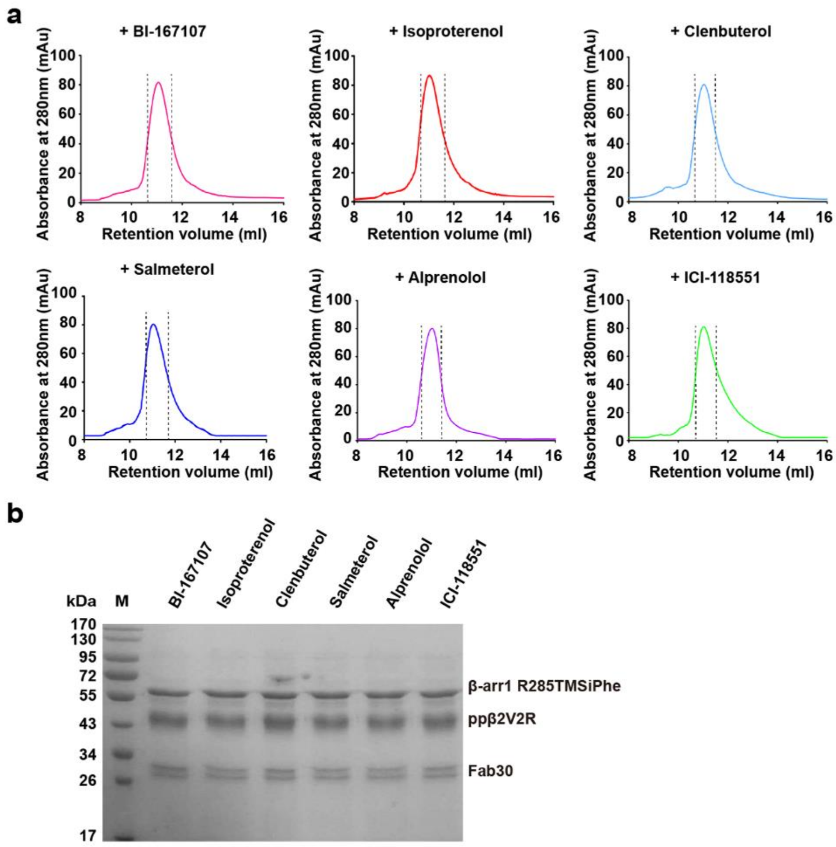

Supplementary Fig. 25. Purification of the complex of ppβ2V2R/β-arr1 R285TMSiPhe/Fab30 with different ligands.

a. ppβ2V2R (30 μM) stimulated with different ligands (60 μM) were incubated with β-arr1 R285TMSiPhe (10 μM) for 30min at 25 ℃. Then Fab30 (20 μM) was added to the mixture and the complex was incubated for 1h at 25 ℃. The ligand/ppβ2V2R/β-arr1 R285TMSiPhe-Fab30 complex was purified by Superdex 200 increase in 20 mM HEPES pH = 7.5, 150 mM NaCl, 0.01% LMNG, 0.002% CHS and corresponding ligand (60 μM). Flow rate was 0.5 ml per minute. The separation yield of complexes were about 50%, and the purities verified by size exclusion chromatography were shown.

b. Coomassie-stained gel showed the purified receptor/arrestin complex, samples were collected at half-height width.

Supplementary Fig. 26

Sample		R0 (0.090 ppm)			R1b (0.068 ppm)	R1a (0.065 ppm)	R2 (0.050 ppm)	Resdiual error	S/N
		Peak areas (x10^2)	Resdiual error	S/N	Peak areas (x10^2)	Peak areas (x10^2)	Peak areas (x10^2)		
R285TMSiPhe -Fab30 -ppβ2V2R	---	4.9	9.3	20.2	---	10.8	---	6.52	18.4 (0.065 ppm)
	Alp	4.2	7.6	20.5	---	12.9	---	29.10	18.2 (0.065 ppm)
	ISO	4.1	10.0	18.1	---	21.1	---	27.30	15.3 (0.065 ppm)
	BI	5.0	13.4	48.9	12.4	---	---	27.20	66.2 (0.068 ppm)
	Clen	5.8	25.3	128.2	---	14.8	5.4	14.10	35.4 (0.065 ppm)
	Salm	4.4	20.2	86.0	---	---	9.4	9.62	42.5 (0.050 ppm)
	ICI	5.5	27.2	28.8	---	---	11.5	6.66	15.1 (0.050 ppm)

Supplementary Fig. 26. ^{1}H-NMR characteristic signal peak position and area analysis of the complex of ppβ2V2R/β-arr1 R285TMSiPhe/Fab30 in the presence of different ligands.

The peak area for each spectrum was calculated by using the automatic baseline correction and fitting region function of the software MestReNova 9.0.1. The fitting region of the R0 and R1/R2 were 0.08~0.1 ppm and 0.03~0.08 ppm respectively, and then the area of the main peaks and corresponding residual error were obtained. Signal-to-noise ratio was measured using 'sinocal' routine within Topspin 4.0 (Bruker Biospin, Billerica MA), on the TMSiPhe signal of β-arr1-R285TMSiPhe at 0.09 ppm or main peak position (for R1/R2), using a 2 ppm noise regions (centered around 11 ppm) for SNR calculations.

Supplementary Fig. 27

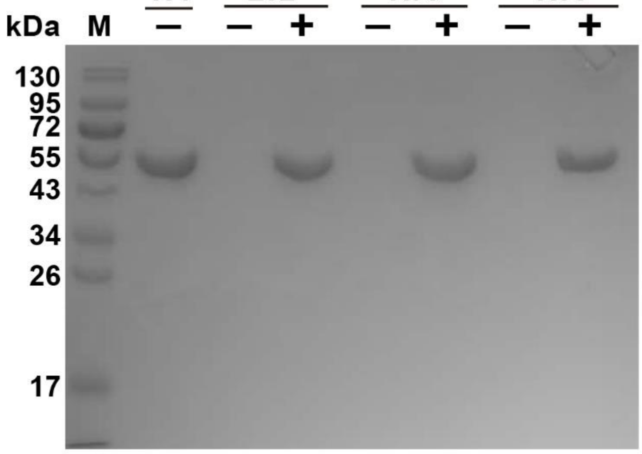

Supplementary Fig. 27. Expression and purification clathrin with TMSiPhe incorporated at

different positions.

The plasmids of clathrin mutants and pEVOL-TMSiPheRS plasmids were co-transformed into BL21(DE3) *E.coli* cell. The *E. coli* cells were cultured in 1 L LB medium. After the cell culture reached OD_{600} = 0.6-0.8 at 37 °C, the cells were induced with 300 μM isopropyl-*β*-D-thiogalactopyranoside (IPTG) and 0.2% L-arabinose for 12 h (25 °C) to allow protein expression in presence or absence of 1 mM TMSiPhe in the culture medium. The cells were lysed by French press in buffer A (50 mM Tris-HCl, pH = 8.0, 150 mM NaCl) and the lysate was batch binding with 300 μL Ni-NTA column (GE Healthcare, USA). After extensive washing with buffer A, the clathrin TMSiphe incorporated protein was eluted using 300 mM imidazole in buffer A. These proteins were subsequently purified by size exclusion column Superdex 75 and the buffer was exchanged to buffer B (20 mM HEPES, pH = 7.5, 150 mM NaCl). Coomassie-stained gel was used to analysis the purified clathrin mutants with TMSiPhe incorporation. +: Presence of 1 mM TMSiPhe in the culture medium; -: Absence of 1 mM TMSiPhe in the culture medium.

Supplementary Fig. 28

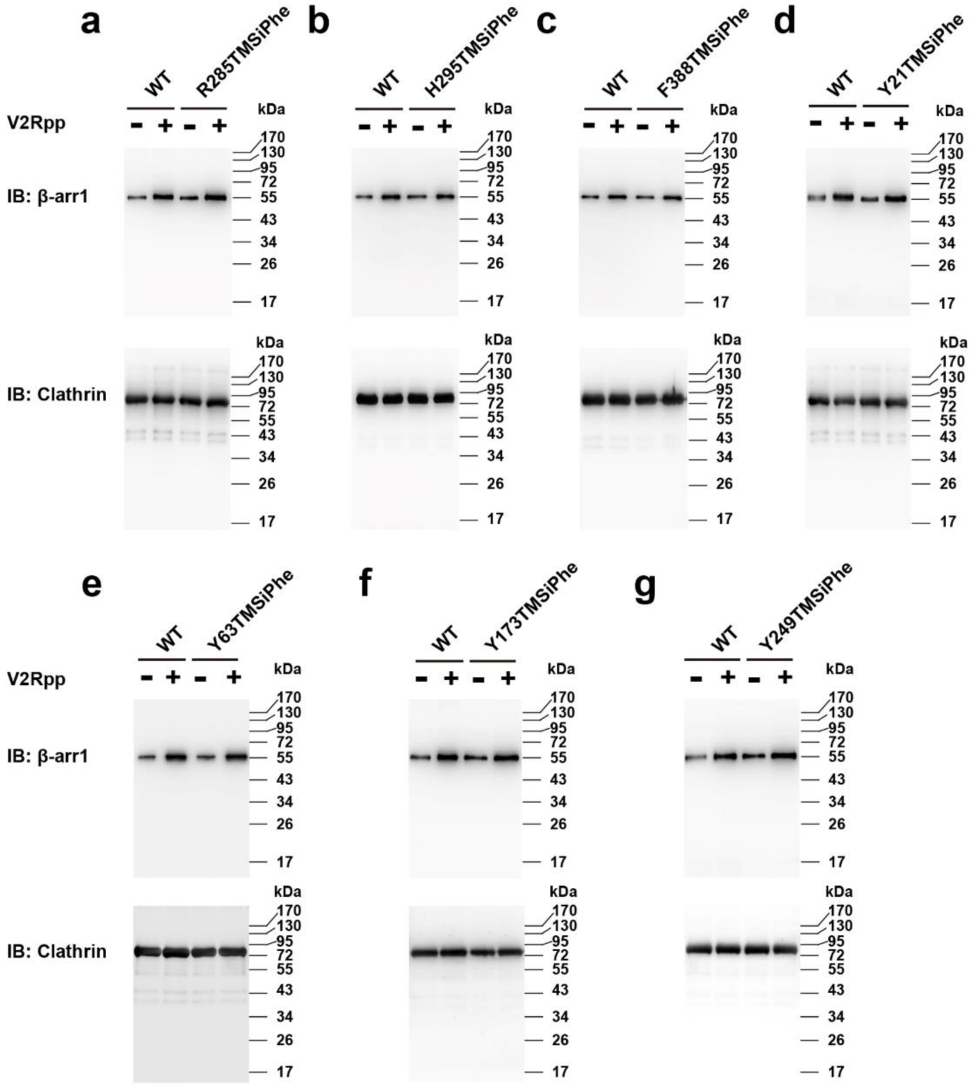

Supplementary Fig. 28. Full blot images of Supplementary figure 14.

Supplementary Table 1. Mass spectrometric analysis of β-arr1 H295TMSiPhe.

b^+	b^{2+}	Sequence	y^+	y^{2+}
114.10	57.55	L		
242.19	121.60	K	1666.88	833.95
461.29	231.15	H295TMSiPhe	1538.79	769.90
590.34	295.67	E	1319.68	660.34
705.36	353.19	D	1190.64	595.82
806.41	403.71	T	1075.61	538.31
920.45	460.73	N	974.56	487.79
1033.54	517.27	L	860.52	430.76
1104.58	552.79	A	747.44	374.22
1191.61	596.31	S	676.40	338.70
1278.64	639.82	S	589.37	295.19
1379.69	690.35	T	502.33	251.67
1492.77	746.89	L	401.29	201.15
1605.86	803.43	L	288.20	144.61
		R	175.12	88.06

The table showed the b ions and y ions of the purified β-arr1 H295TMSiPhe in LC-MS/MS spectrometry.

Supplementary Table 2. Data collection and refinement statistics.

	sfGFP Y182TMSiPhe	TMSiPheRS alone	TMSiPheRS-TMSiPhe complex
**Data Collection**			
Space group	$I4_1$	$P2_12_12_1$	$P2_12_12_1$
Cell Dimensions			
a, b, c (Å)	68.57, 68.57, 119.26	71.16, 91.20, 107.52	71.10, 83.43, 106.24
α, β, γ (deg)	90, 90, 90	90, 90, 90	90, 90, 90
Resolution (Å)	50.00-1.40 (1.42-1.40)*	49.75-1.79 (1.83-1.79)*	48.22-2.05 (2.11-2.05)*
R_{sym} or R_{merge}	0.048 (0.438)	0.049 (0.967)	0.071 (0.580)
I / σ	32.4 (2.8)	16.8 (1.6)	9.5 (1.7)
Completeness (%)	99.7 (99.9)	99.6 (99.0)	96.2 (95.1)
Redundancy	6.2 (6.2)	6.6 (5.8)	2.6 (2.5)
**Refinement**			
Resolution (Å)	29.72-1.40 (1.45-1.40)	49.75-1.79	48.22-2.05
No. reflections	53772	65701	38432
R_{work}/ R_{free}	0.148/0.174	0.206/0.246	0.207/0.260
No. atoms	2088	5372	5127
Protein	1769	4991	4989
Ligand/ion	53	---	16
Water	266	381	122
B-factors(Å^2)	26.40	46.32	35.00
Protein(Å^2)	24.75	46.11	35.01
Ligand/ion(Å^2)	24.78	---	43.71
Water(Å^2)	37.73	49.01	33.50
R.M.S. deviations			
Bond lengths (Å)	0.005	0.006	0.011
Bond angles (deg)	0.77	0.80	1.06

*** Number of xtals for each structure should be noted in footnote. *Highest-resolution shell is shown in parentheses.**

[AU: Equations defining various R values are standard and hence are no longer defined in the footnotes.]
[AU: Ramachandran statistics should be in methods section at the end of the refinement sub-section.]
[AU: Wavelength of data collection, temperature, beamline should all be in methods section.]

Supplementary Table 3. The TMSiPhe incorporation sites in β-arr1 and their proposed functions reported in the literature.

Position	Description	Key literature
Y21	Site that exposes to GPCR phosphorylated C-tail interfaces	Shukla. et al., *Nat.* 2013; Yang et al., *Nat. Comm.* 2015
Y63	Finger loop region relate to GPCR binding and arrestin activation	Kang et al., *Nat.* 2015; Latorraca et al., *Nat.* 2018
Y173	Switch regions in arrestin-mediated signaling.	Chen et al., *NC*. 2017
Y249	Site region that exhibits arrestin coupling with inositol hexakisphosphate (IP6)	Gaidarov et al., *EMBO J*. 1999
R285	Site region mutations of arrestin that reduced β2AR dependent ERK1/2 activation	Xu et al., *Biochem J*. 2008
H295	Arrestin polar core region interaction with GPCR	Hirsch et al., *Cell. 1999*; Shukla. et al., *Nat.* 2013; Zhou et al., *Cell*. 2017 ; Chen et al., *Trends Biochem Sci*. 2018
F388	β arrestin 1 C tail region interaction with AP2	Schmid et al., *PLOS.Bio*. 2006

Supplementary Table 4. Purification and yield of TMSiPhe incorporated β-arr1 at selective positions

Mutation	Purification yield	Degree of purity	Amount of purified proteins
Y21	75%	>95%	1.5mg/L
Y63	70%	>95%	1.0mg/L
Y173	75%	>95%	1.5mg/L
Y249	70%	>95%	1.0mg/L
R285	75%	>95%	1.5mg/L
H295	75%	>95%	1.5mg/L
F388	65%	>95%	0.8mg/L

Supplementary Table 5. A complete list of all primers used in our study.

Name	Sequence
β-arr1 Y21TAG-F	ACCGTCTAGCTGGGAAAGCGGGACTTT
β-arr1 Y21TAG-R	TCCCAGCTAGACGGTGAGCTTTCCATT
β-arr1 Y63TAG-F	TTCCGCTAGGGCCGGGAGGACCTGGAT
β-arr1 Y63TAG-R	CCGGCCCTAGCGGAAGGCGCAGGTCAG
β-arr1 Y173TAG-F	GTTCAGTAGGCCCCAGAGAGGCCTGGC
β-arr1 Y173TAG-R	TGGGGCCTACTGAACCTTCCGGATGAC
β-arr1 Y249TAG-F	GCCCAGTAGAAGTGCCCTGTGGCCATG
β-arr1 Y249TAG-R	GCACTTCTACTGGGCTGTGTTGAACAG
β-arr1 R285TAG-F	GAGAAGTAGGGCCTCGCCCTGGACGGG
β-arr1 R285TAG-R	GAGGCCCTACTTCTCTCGATTGTTGGC
β-arr1 H295TAG-F	CTCAAATAGGAGGACACGAACCTGGCC
β-arr1 H295TAG-R	GTCCTCCTATTTGAGCTTCCCGTCCAG
β-arr1 F388TAG-F	ATTGTGTAGGAGGACTTTGCCCGCCAG
β-arr1 F388TAG-R	GTCCTCCTACACAATGTCGTCATCGTT
sfGFP Y182TAG-F	GACCACTAGCAGCAGAACACCCCGATC
sfGFP Y182TAG-R	CTGCTGCTAGTGGTCAGCCAGCTGAAC
pFast-β2V2R-F	GATGAGTCCTGCACCACCGCCAGCTCCTCCCTGGCCAAGG ACACTTCATCGCACCACCACCACCACCACCATCATCATCATTAA
pFast-β2V2R-R	GGTGCAGGACTCATCTTGGGGACCCAGGCTGGGTGGGGTGCGT CCCCGGGCGCACAGAAGCTCCTGGAAGGCAATCCTGAAATCTGG
β2AR-Δ5-I135W-F	TTTGCCTGGACTTCACCTTTCAAGTAC
β2AR-Δ5-I135W-R	TGAAGTCCAGGCAAAGTAGCGATCCAC
β2AR-Δ5-I135W-A271C-F	CACAAATGCCTCAAGACGTTAGGCATC
β2AR-Δ5-I135W-A271C-R	CTTGAGGCATTTGTGCTCCTTCAAGCA
pcDNA3.1-β2V2R-vector-F	GCTTGCGGTACCGGATCCCACCACCACCACCAC CACCATCATCATCATTAA
pcDNA3.1-β2V2R-vector-R	GTACACCTTGCTGGTCATCGATGAAGTGTCCTTG GCCAGGGAGGAGCTGGC
gene of Rluc-F	ATGACCAGCAAGGTGTACGACCCCGAG
gene of Rluc-R	GGATCCGGTACCGCAAGCTTGATATCC
pET22b-vector-TMSiPhe-F	CCAATTAGAAAGAGATTACTCGAGCACCACCACCACCACCACTGA
pET22b-vector-TMSiPhe -R	CATTTCAAATTCGTCCATATGTATATCTCCTTCTTAAAGTTAAAC
gene of TMSiPheRS-F	ATGGACGAATTTGAAATGATAAAGAGAAAC
gene of TMSiPheRS-R	TAATCTCTTTCTAATTGGCTCTAAAATCTT
Clathrinn-L82TAG-F	ATTGCATAGAAAGCTGGAAAAACTCTACAG
Clathrinn-L82TAG-R	AGCTTTCTATGCAATCACTTTGCTGGCAGG
Clathrinn-K96TAG-F	GAAATGTAGAGTAAAATGAAGGCCCAC
Clathrinn-K96TAG-R	TTTACTCTACATTTCAATGTTAAATAT

Clathrinn-K98TAG-F	AAAAGTTAGATGAAGGCCCACACCATGACC
Clathrinn-K98TAG-R	CTTCATCTAACTTTTCATTTCAATGTTAAA

Supplementary References

1 Kenkichi, N. et al. Applications of NMR especially in analytical chemistry. Journal of the Society of Instrument and Control Engineers 1(2):89-94 (1962)

2 Hull, W. E. & Sykes, B. D. Fluorotyrosine alkaline phosphatase: internal mobility of individual tyrosines and the role of chemical shift anisotropy as a 19F nuclear spin relaxation mechanism in proteins. *J Mol Biol* **98**, 121-153 (1975).

3 Chen, W. N. *et al.* O-tert-Butyltyrosine, an NMR tag for high-molecular-weight systems and measurements of submicromolar ligand binding affinities. *J Am Chem Soc* **137**, 4581-4586 (2015).

4 Liu, C. H. *et al.* Arrestin-biased AT1R agonism induces acute catecholamine secretion through TRPC3 coupling. *Nat Commun* **8**, 14335 (2017).

5 Yao, X. *et al.* Coupling ligand structure to specific conformational switches in the beta2-adrenoceptor. *Nat Chem Biol* **2**, 417-422 (2006).

6 Bundi A, Wuthrich K. H-1-NMR Parameters of the Common Amino-Acid Residues Measured In Aqueous-Solution Of the Linear Tetrapeptides H-Gly-Gly-X-L-Ala-Oh. *Biopolymers* **18**: 285-297 (1979).

7 Wang, J. *et al.* Synthesis of β2-AR Agonist BI-167107. *Chinese. J. Org. Chem* **33**, 634-639 (2013).
