# Supplementary material for: DeSiphering receptor core-induced and ligand-dependent conformational changes in arrestin via genetic encoded trimethylsilyl 1H-NMR probe
Source: Nat Commun. 2020 Sep 25;11:4857. doi: 10.1038/s41467-020-18433-5 (PMC7519161; doi:10.1038/s41467-020-18433-5)
Supplement: Supplementary file 2 — Peer Review file [file 41467_2020_18433_MOESM2_ESM.docx]

Peer Review Information

**Journal:** Nature Communications

**Manuscript title:** DeSiphering receptor core-induced and ligand-dependent conformational changes in arrestin via genetic encoded Trimethylsilyl 1H-NMR Probe

**Corresponding author name(s):** Prof Jinpeng Sun

**Editorial notes:**

**none**

**Reviewer comments & decisions:**

**Reviewer comments, first version:**

Reviewer #1 (Remarks to the Author):

Sun and Wang et al. have developed a new trimethylsilyl containing amino acid for probing conformation changes in large membrane protein complexes. This work is a nice addition to other unnatural amino acids. The authors developed a tRNA synthetase that can incorporate the unnatural amino acid 4-trimethylsilylphenylalanine into proteins to study conformational changes. They then incorporated the TMSiPhe into β-arrestin-1 at several different sites and used 1H NMR to monitor changes in the conformation of β-arrestin-1.The work performed in the paper is well done but the manuscript feels like it was written by several others and they figures have no consistency between them.
While the work is well done, I do not think this manuscript is right for Nature Communications. I feel it would be better suited to a more niche journal focusing on structural biology. While the manuscript does provide a novel tool to probe the confirmation changes of large membrane protein complexes, it is not the first to do so and some of there are some concerns that I have about the paper.
1) My biggest concern it that the authors raise a talk about the time required to use 19F labeled proteins for the same type of work as well as the amount of protein needed and the cost of instrumentation. While this is indeed a concern for the use of 19F, in this manuscript all spectra are taken on a 950 MHz spectrometer equipped with a cryoprobe. By using a such a system, that are found in very few research institutions, it takes away from the argument. They are using an extremely expensive and highly sensitive instrument to acquire the data. To me this is a major issue based on their claims. The authors need to show that they can still acquire the same quality data on lower field instruments (600, 700, or 800 MHz) in the same time frame with the same concentration of protein, or show how long it would take to get data with the same S/N on the lower field instruments.
2) The incorporation of a large TMS group to phenylalanine appears to have a large perturbation on the structure of the protein. In figure 3b, there are large changes in the signals at 0.7-1.0 ppm. In the R285TMSiPHe mutant the large right-hand signal is almost gone. It looks like incorporation this new amino acid has had a large change on the proteins conformation. The authors should comment on this. Incorporation of 19F is much smaller and has a much smaller impact on the protein.
3) In the manuscript the authors only show the TMSiPhe in one system, albeit at different sites, where the changes to confirmation were already known. It would be useful if the authors could show that TMSiPhe works in a new system to show a previously unknown conformation change.
4) In figure 1b, the authors show a gel from the use of their new tRNA synthetase with several TMS containing amino acids. Where is the band from the incomplete protein that should be formed in lanes 2-5?
5) In figure 3e the authors shows that the KD of V2Rpp binding 6.9 uM, but the spectra in figure 3d show that at 10 uM there is still more of the inactive that active form so the KD must be greater that 10 uM.
6) Line 145 ends abruptly
7) Line 150, extra NMR at the end.
8) When talking about the β2AR ligands the authors claim that Alp showed no effect on the S3 state, while ICI-118551 reduced the population of S3. However figure S15 shows that both Alp and ICI-118551 have the same but opposite effect on the ration of S3/S1. How can you say that one has no effect but the other does when they are the same amount?
9) Line 441 subscript Na2SO4.
10) Throughout synthesis of TMSiPhe section; superscript 1 of 1H NMR. subscript 3 of CDCl3
11) starting at the Purification of TMSiPheRS section to the end. Need a space between the number and degrees symbol.
12) line 574, what does the 25 in parenthesis mean?
13) line 584, space before 10
14) line 585, no degrees symbol after -80.
15) line 610, change cytoprobe for cryoprobe. what kind of cryoprobe?
16) lines 611-613, why give NS and then say that the NS is changed base on concentration?
17) line 819, ref 53 contains non-english characters.
18) the figures and graphs need to have better axis labels and units need to be place in parenthesis not after a forward slash.


Reviewer #2 (Remarks to the Author):

The manuscript by Sun and co-workers described the development of the site-specific incorporation of 4-trimethylsilyl phenylalanine (TMSiPhe) into proteins for 1D, <sup>1</sup>H-NMR analyses, and its application to the conformational analyses of β-arrestin-1. The authors identified a mutant tRNA synthetase with specific activity toward TMSiPhe, which enabled the efficient and selective TMSiPhe incorporation to proteins of interests. Using the developed method, <sup>1</sup>H NMR resonances from TMSiPhe in 5 μM β-arrestin-1 were detected within 20 min. In addition, the authors investigated the conformational change of β-arrestin-1 upon addition of phosphorylated peptide (V2Rpp) or phosphorylated GPCR (ppβ2V2R) bound to various ligands.

The results about the establishment of TMSiPhe labeling are clearly presented and hardly controversial. Although the utilization of the trimethylsilyl group as 1D, <sup>1</sup>H-NMR probe (Loh et al., J. Biomol. NMR (2018) 71, 287-293) and its application to the G protein-coupled receptor (Hu et al., FEBS Lett (2019) 593, 1113-1121) have been reported, the method described here dramatically improved the labeling selectivity and efficiency, thus was the useful contribution. On the other hand, I have some concerns about the interpretation of the conformational analyses of β-arrestin-1 with ppβ2V2R and Fab30.

Therefore, I think a major revision of manuscript is needed before it can be accepted for publication.

Comments

1. In the Abstract and Introduction sections, the authors mainly described the achievement of the methodology, and significance about the investigation of β-arrestin-1 were not sufficiently described. On the other hand, more than half of the Results and Discussion sections are spent for the description about the conformational investigation of β-arrestin-1. The Abstract and Introduction sections should be revised.

2. In line 245-250, the conformational change of the β-arrestin-1 upon interaction with V2Rpp was clearly demonstrated by NMR titration experiments. It could be worth checking the conformational effects of the Fab30 binding toward the β-arrestin-1/V2Rpp complex, by using Fab30 titration, to compare with the following NMR spectra of the β-arrestin-1/ppβ2V2R/Fab30 mixture. Although the authors investigated the effect of Fab30 in supplementary Fig. S13, this experiment was conducted in the absence of V2Rpp and thus not suitable to investigate the effect of the Fab30 binding to β-arrestin-1.

3. In line 251-281, the NMR spectra of the β-arrestin-1/ppβ2V2R/Fab30 mixtures with various ligands were obtained. The S1 resonance corresponding to inactive free β-arrestin-1 are observed in all of the spectra containing ppβ2V2R and Fab30. Do the residual S1 resonances suggest that parts of β-arrestin-1 in the NMR sample were not bound to ppβ2V2R and Fab30, or that conformation of β-arrestin-1 bound to ppβ2V2R and Fab30 was in exchange between inactive and active conformation? To surely interpret these spectra, it could be worth checking the effect of the titration of the increasing concentration of ppβ2V2R to β-arrestin-1.

4. In line 286-300, the spectral change of β-arrestin-1/ppβ2V2R/Fab30 upon addition Gα-CT was observed. If the Gα-CT blocked the interaction between β-arrestin-1 and TM core of ppβ2V2R, β-arrestin-1 interact with the phosphorylated C-tail of β2V2R (and Fab30), as depicted in Fig. 5a. This situation would be identical with the V2Rpp-bound state. Thus, considering the results of the V2Rpp titration experiment in Fig. 4c, the S2 resonance would be detected in the NMR spectrum of β-arrestin-1/ppβ2V2R/Fab30/Gα-CT, in the bottom of Fig. 5c. Why the S2 resonance was not observed? Does the β-arrestin-1 in the NMR sample really form “hanging” interaction with ppβ2V2R?

5. In line 301-310, the authors claimed that β-arrestin-1/ppβ2V2R/Fab30 complex was dissociated by the addition of V2Rpp, and the active conformation still exists even after the dissociation. As the authors described in line 308-310, the growing evidences suggested that arrestins maintain their activity after the dissociation from receptor in cellular context. However, the reported rate constant of the reversal of the active conformation was 23 s (Nuber et al., Nature (2016) 661-664), which is much faster than the experimental time of the NMR measurement (40 min described in line 622). In addition, the reported stabilization of the arrestin activity after the dissociation from receptor requires non-GPCR partners such as phosphoinositide (Eichel et al., Nature (2018) 557, 381-386), which were not contained in the NMR sample. Therefore, the conformation of the β-arrestin-1 should be relaxed to the inactive form when β-arrestin-1 was fully dissociated from the ppβ2V2R and Fab30. There are the possibilities that the S3 resonance in the β-arrestin-1/ppβ2V2R/Fab30/V2Rpp sample was from the residual β-arestin-1 bound to ppβ2V2R and/or Fab30, or the irreversibly denatured or degradated β-arrestin-1 in the NMR sample.

6. In line 360-372, the authors claimed the high sensitivity of the <sup>1</sup>H resonances from TMSiPhe groups by comparing with the sensitivity of the <sup>19</sup>F resonances from 3,5- difluorotyrosine (F2Y) probe. The principle of the sensitivity gain should be thoroughly described here for clearity. In general, intrincic sensitivity of <sup>19</sup>F NMR signals is comparable with <sup>1</sup>H NMR signals since the gyromagnetic ratio of <sup>1</sup>H and <sup>19</sup>F are comparable. It seems that the main reason of the higher sensitivity of the TMSiPhe groups than F2Y groups are not due to the kind of nuclei (<sup>19</sup>F vs <sup>1</sup>H), but the number of magnetically equivalent spins (nine in TMSiPhe vs two in F2Y) and the slow relaxing nature of the methyl groups.

**Author rebuttal, first version:**

**Reviewer #1 (Remarks to the Author):**

Sun and Wang et al. have developed a new trimethylsilyl containing amino acid for probing conformation changes in large membrane protein complexes. This work is a nice addition to other unnatural amino acids.

**Reply:** We thank the reviewer for his positive comments.

The authors developed a tRNA synthetase that can incorporate the unnatural amino acid 4-trimethylsilylphenylalanine into proteins to study conformational changes. They then incorporated the TMSiPhe into β-arrestin-1 at several different sites and used ^1^H NMR to monitor changes in the conformation of β-arrestin-1. The work performed in the paper is well done but the manuscript feels like it was written by several others and they figures have no consistency between them. While the work is well done, I do not think this manuscript is right for Nature Communications. I feel it would be better suited to a more niche journal focusing on structural biology. While the manuscript does provide a novel tool to probe the confirmation changes of large membrane protein complexes, it is not the first to do so and some of there are some concerns that I have about the paper.

1)	My biggest concern is that the authors raise a talk about the time required to use 19F labeled proteins for the same type of work as well as the amount of protein needed and the cost of instrumentation. While this is indeed a concern for the use of 19F, in this manuscript all spectra are taken on a 950 MHz spectrometer equipped with a cryoprobe. By using a such a system, that are found in very few research institutions, it takes away from the argument. They are using an extremely expensive and highly sensitive instrument to acquire the data. To me this is a major issue based on their claims. The authors need to show that they can still acquire the same quality data on lower field instruments (600, 700, or 800 MHz) in the same time frame with the same concentration of protein, or show how long it would take to get data with the same S/N on the lower field instruments.

**Reply:** We thank the reviewer for his valuable comments and helpful suggestions. We compared the NMR signals of TMSiPhe generated by 600, 800 and 950 MHz instruments, as listed in Supplemental Figure 6. Even the 600 MHz NMR instrument could provide an unambiguous NMR signal with an experimental condition of 50 μM protein with a data collection time of less than one hour (Figure S6a-6b). With longer collection time and higher protein concentration, 800 MHz NMR could provide a signal similar to that obtained with the 950 MHz NMR instrument (Figure S6a-S6b). As 800 MHz NMR instruments are available in many institutes and 600 MHz NMR instruments can be found in most universities, the DeSipher method could be widely applied. We have included the corresponding discussion in the main text (page 6, lines 187-191, and page 12, lines 419-426).

2) The incorporation of a large TMS group to phenylalanine appears to have a large perturbation on the structure of the protein. In figure 3b, there are large changes in the signals at 0.7-1.0 ppm. In the R285TMSiPHe mutant the large right-hand signal is almost gone. It looks like incorporation this new amino acid has had a large change on the proteins conformation. The authors should comment on this. Incorporation of ^19^F is much smaller and has a much smaller impact on the protein.

**Reply:**

We thank the reviewer for his valuable comment. In previous measurements, the R285TMSiPhe protein remained at room temperature for approximately 1 day before measurement, which could be the cause of the unusual signals. We prepared fresh β-arrestin-1 R285TMSiPhe protein and performed the corresponding measurement again. The signal of the newly prepared β-arrestin-1 R285TMSiPhe in the proton NMR spectrum within the range of 0.5 ppm to 1.0 ppm was quite similar to that of TMSiPhe incorporated at other positions in β-arrestin-1 (new Figure. 3b in the revised manuscript). To validate the finding that TMSiPhe incorporation did not affect the functional integrity of the arrestin R285TMSiPhe, we conducted functional testing, as shown in Figure S12a. The results indicated that R285TMSiPhe did not affect V2Rpp-induced arrestin activation in the clathrin binding assay, thus indicating its functional integrity.

3) In the manuscript the authors only show the TMSiPhe in one system, albeit at different sites, where the changes to conformation were already known. It would be useful if the authors could show that TMSiPhe works in a new system to show a previously unknown conformation change.

**Reply:** We thank the reviewer for his valuable comment and helpful suggestions. Previously, we used an F2Y NMR probe to detect the conformational states of arrestin in response to receptor or phosphopeptide engagement. The NMR probe labeling sites in these two paper only restrict to SRC functional related positions (*Nat Commun 6, 8202, (2015), Nat Chem Biol 14, 876-886, (2018)*). Here, we labeled new functional sites in arrestin that exemplified the conformational states of the polar core and ERK interaction sites and have not been characterized previously by biophysical methods, such as NMR (Figure 4 and Figure 6). Moreover, how different ligands of the same receptor regulate selective function-related arrestin conformations has never been examined, mostly due to the large amount of receptor protein required. Therefore, application of the TMSiPhe method enabled us to examine for the first time these functionally related conformations of arrestin, for example, the ERK interaction sites, in response to different receptor ligands, which has not been probed before.

Moreover, according to the helpful suggestions provided by the reviewer, we incorporated the TMSiPhe probe into clathrin, a key signaling hub downstream of the receptor, to mediate receptor internalization. The conformational change of clathrin could be detected by the TMSiPhe probe in response to incubation with the receptor-arrestin complex, thus providing a useful avenue for future receptor biology studies (new Figure 7 and new Figure S25 in the revised manuscript).

4) In figure 1b, the authors show a gel from the use of their new tRNA synthetase with several TMS containing amino acids. Where is the band from the incomplete protein that should be formed in lanes 2-5?

**Reply:** We thank the reviewer for his valuable comments. The expression vector for TMSiPhe-containing arrestin was pET22B, which attaches a 6x His tag at the carboxyl terminus of the target protein. The protein was then purified by a Ni^+^ column, thereby discarding all the incomplete protein during purification. We have incorporated these descriptions in the methods section (page 18, lines 635-636).

5) In figure 3e the authors shows that the KD of V2Rpp binding 6.9 μM, but the spectra in figure 3d show that at 10 μM there is still more of the inactive that active form so the KD must be greater that 10 μM.

Reply:

We thank the reviewer for his valuable comments. In general, the KD could be derived from the equation KD=[arrestin_free_] [V2Rpp_free_] / [arrestin-V2Rpp]= [arrestin_free_] [V2Rpp_total_-V2Rpp_bound_] / [arrestin-V2Rpp] . Only when the concentration of arrestin is significantly lower than that of V2Rpp can the half active:inactive equilibrium for V2Rpp at the KD value be achieved. However, under our experimental conditions, we used comparable concentrations of arrestin (20 μM) and V2pp (ranging from 10 μM to 180 μM), leading to much lower levels of V2Rpp_free_ than of V2Rpp_total_. When the V2Rpp_total_ is 10 μM, the real V2Rpp_free_ is still lower than the KD value because large amounts of V2Rpp were bound to arrestin. Therefore, there remains a large amount of the inactive form of arrestin at 10 μM V2Rpp. For example, when the V2Rpp concentration is 10 μM, according to the signal peak area calculation, the arrestin-V2Rpp concentration is 6.5 μM; therefore, the arrestin_free_ concentration is 13.5 μM. Because of the 1:1 ratio in the arrestin complex, it can be deduced that the V2Rpp concentration is also 6.5 μM in the complex state and 3.5 μM in the free state. Through calculation with the equation, the KD=[arrestin] [V2Rpp] / [arrestin – V2Rpp] = 13.5 * 3.5/6.5 = 7.3 μM. This is close to the final result of 6.9 μM obtained from curve fitting.

6) Line 145 ends abruptly

**Reply:** We thank the reviewer for his valuable comments. We have revised corresponding part to “Moreover, the electron density of TMSiPhe was clearly observed in the crystal structure of a TMSiPhe-containing green fluorescence protein (GFP), an easily crystallized protein (Figure 1d and Table S2). Crystallization of GFP-Y182TMSiPhe takes approximately 1 week at 16 °C, thus confirming the stability of TMSiPhe after incorporation into a particular protein.” (line 173-176 in the revised manuscript)

7) Line 150, extra NMR at the end.

Reply: We thank the reviewer for his valuable and helpful suggestions. We have deleted the extra NMR in the revised manuscript (line 177).

8) When talking about the β2AR ligands the authors claim that Alp showed no effect on the S3 state, while ICI-118551 reduced the population of S3. However figure S15 shows that both Alp and ICI-118551 have the same but opposite effect on the ration of S3/S1. How can you say that one has no effect but the other does when they are the same amount?

**Reply:** We thank the reviewer for his valuable comments. We have added the ΔS3/S1 column to Figure S17 in the revised manuscript. We have revised the main manuscript accordingly, describing that Alp and ICI-118551 both have very little effect on the S3 state compared to other partial or full agonists (lines 305-306 in the revised manuscript).

9) Line 441 subscript Na2SO4.

Reply: We thank the reviewer for his valuable comments. We have revised this part accordingly.

10) Throughout synthesis of TMSiPhe section; superscript 1 of 1H NMR. subscript 3 of CDCl3

Reply: We thank the reviewer for his valuable comments. We have revised these parts accordingly.

11) Starting at the Purification of TMSiPheRS section to the end. Need a space between the number and degrees symbol.

Reply: We thank the reviewer for his valuable comments. We have revised these parts accordingly.

12) line 574, what does the 25 in parenthesis mean?

Reply: This is the temperature indicating for E.Coli cell culture. We have revised this part accordingly: “The cells were induced with 300 μM isopropyl-β-D-thiogalactopyranoside (IPTG) and 0.2% L-arabinose for 12 h at 25 °C to allow protein expression in presence of 0.5mM TMSiPhe in the culture medium.”

13) line 584, space before 10

Reply: We thank the reviewer for his valuable comments. We have revised this part accordingly.

14) line 585, no degrees symbol after -80.

Reply: We thank the reviewer for his valuable comments. We have revised this part accordingly.

15) line 610, change cytoprobe for cryoprobe. what kind of cryoprobe?

Reply: We thank the reviewer for his valuable comments. We have revised this part accordingly. The cryoprobe is proton-optimized Triple (TCI) Resonance cryoprobe (1H, 13C, 15N).

16) lines 611-613, why give NS and then say that the NS is changed base on concentration?

**Reply:** NS represents the cumulative times of the NMR test, and NS=32 is generally used for high-concentration proteins with low molecular weights. We preliminarily observed NS=32 by using the signal peak time. To further improve the S/N ratio and facilitate data analysis, we increased the NS value to 256 during data collection. Due to the different test times required for samples with different concentrations, appropriate NS values were adjusted accordingly.

17) line 819, ref 53 contains non-english characters.

Reply: This is a paper in Japanese about NMR. The title “ 核磁気共鳴-とくに分析化学への応用 ” has been replaced by “ Applications of NMR especially in analytical chemistry ” in the revised manuscript.

18) the figures and graphs need to have better axis labels and units need to be place in parenthesis not after a forward slash.

Reply: We thank the reviewer for his valuable comments. We have revised these parts accordingly including axis labels、curves and units in Figures 1a, 1c, 1e, 3b, 3d, 3e, 4c, 4d, 4f, 5c, 6b, 6c and Figure S1a, S2b, S4, S5b, S9, S10, S16, S17, S19, S22 respectively.

**Reviewer #2 (Remarks to the Author):**

The manuscript by Sun and co-workers described the development of the site-specific incorporation of 4-trimethylsilyl phenylalanine (TMSiPhe) into proteins for 1D, 1H-NMR analyses, and its application to the conformational analyses of β-arrestin-1. The authors identified a mutant tRNA synthetase with specific activity toward TMSiPhe, which enabled the efficient and selective TMSiPhe incorporation to proteins of interests. Using the developed method, 1H NMR resonances from TMSiPhe in 5 μM β-arrestin-1 were detected within 20 min. In addition, the authors investigated the conformational change of β-arrestin-1 upon addition of phosphorylated peptide (V2Rpp) or phosphorylated GPCR (ppβ2V2R) bound to various ligands.

The results about the establishment of TMSiPhe labeling are clearly presented and hardly controversial. Although the utilization of the trimethylsilyl group as 1D, ^1^H-NMR probe (Loh et al., J. Biomol. NMR (2018) 71, 287-293) and its application to the G protein-coupled receptor (Hu et al., FEBS Lett (2019) 593, 1113-1121) have been reported, the method described here dramatically improved the labeling selectivity and efficiency, thus was the useful contribution.

**Reply:** We thank the reviewer for his positive comments.

On the other hand, I have some concerns about the interpretation of the conformational analyses of β-arrestin-1 with ppβ2V2R and Fab30. Therefore, I think a major revision of manuscript is needed before it can be accepted for publication.

Comments

1. In the Abstract and Introduction sections, the authors mainly described the achievement of the methodology, and significance about the investigation of β-arrestin-1 were not sufficiently described. On the other hand, more than half of the Results and Discussion sections are spent for the description about the conformational investigation of β-arrestin-1.

**Reply:** We thank the reviewer for his valuable comments. We have revised the abstract and introduction sections accordingly. The background for arrestin introduction was shown in line 115-127 in the revised manuscript.

2. In line 245-250, the conformational change of the β-arrestin-1 upon interaction with V2Rpp was clearly demonstrated by NMR titration experiments. It could be worth checking the conformational effects of the Fab30 binding toward the β-arrestin-1/V2Rpp complex, by using Fab30 titration, to compare with the following NMR spectra of the β-arrestin-1/ppβ2V2R/Fab30 mixture. Although the authors investigated the effect of Fab30 in supplementary Fig. S13, this experiment was conducted in the absence of V2Rpp and thus not suitable to investigate the effect of the Fab30 binding to β-arrestin-1.

**Reply:** We thank the reviewer for his valuable comments and helpful suggestions. We checked the conformational effects of Fab30 binding on the β-arrestin-1/V2Rpp complex by using Fab30 titration at 0 μM, 4 μM, 8 μM, 16 μM, and 32 μM (Figure S13). The results indicated that Fab30 stabilized the active arrestin conformation in a concentration-dependent manner. We have included the corresponding description on page 8, lines 277-279, in the revised manuscript.

3. In line 251-281, the NMR spectra of the β-arrestin-1/ppβ2V2R/Fab30 mixtures with various ligands were obtained. The S1 resonance corresponding to inactive free β-arrestin-1 are observed in all of the spectra containing ppβ2V2R and Fab30. Do the residual S1 resonances suggest that parts of β-arrestin-1 in the NMR sample were not bound to ppβ2V2R and Fab30, or that conformation of β-arrestin-1 bound to ppβ2V2R and Fab30 was in exchange between inactive and active conformation? To surely interpret these spectra, it could be worth checking the effect of the titration of the increasing concentration of ppβ2V2R to β-arrestin-1.

Reply:

We thank the reviewer for his valuable comments and helpful suggestions. As suggested by the reviewer, we have checked the effects of titration with increasing concentrations of ppβ2V2R on the S1 and S3 resonances by using ppβ2V2R at concentrations of 0 μM, 2 μM, 4 μM, 8 μM, 16 μM, and 32 μM. The results indicated that the S3 signal gradually increased and the S1 signal gradually decreased in response to the increasing ppβ2V2R concentration (Figure S18). The maximal receptor concentration that we used was 32 μM, equal to a concentration of approximately 5 mg/ml receptor complex. The NMR signal for a higher concentration than that could not be determined because of receptor aggregation. Therefore, we could only deduce a saturation curve using the data that we acquired. From the simulated curve, S3:S1 will be close to 2:3 even at the saturated concentration of ppβ2V2R, which is likely the concentration at which the maximum amount of arrestin is bound to the receptor (Figure. S18b). Therefore, it is very likely that the conformational state of the β-arrestin-1 H295TMSiPhe site was switching between inactive and active conformations because of the protein’s own flexibility, even under the saturated receptor condition. With a lower receptor concentration, the residual S1 resonances reflect both the unbound arrestin in the inactive state and the bound arrestin conformation that switches between the active and inactive states. We have included corresponding descriptions on page 8, lines 293-294, in the main manuscript and in the figure legend for Figure S18.

4. In line 286-300, the spectral change of β-arrestin-1/ppβ2V2R/Fab30 upon addition Gα-CT was observed. If the Gα-CT blocked the interaction between β-arrestin-1 and TM core of ppβ2V2R, β-arrestin-1 interact with the phosphorylated C-tail of β2V2R (and Fab30), as depicted in Fig. 5a. This situation would be identical with the V2Rpp-bound state. Thus, considering the results of the V2Rpp titration experiment in Fig. 4c, the S2 resonance would be detected in the NMR spectrum of β-arrestin-1/ppβ2V2R/Fab30/Gα-CT, in the bottom of Fig. 5c. Why the S2 resonance was not observed? Does the β-arrestin-1 in the NMR sample really form “hanging” interaction with ppβ2V2R?

**Reply:** We thank the reviewer for his valuable comments. We did not see any signal at the S2 position once we used the Gα-CT peptide for competition. We predicted that addition of the Gα-CT peptide would disrupt the interaction between the receptor core and arrestin but transit arrestin to a hanging conformation. In a recently solved ppβ2V2R/Gs/β-arrestin-1/Fab30 supercomplex structure (*Nat. Struct. Mol. Biol.* 26, pages 1123–1131 (2019)), which closely mimics the hanging conformation under conditions similar to ours, the arrestin is entirely covered with detergent, which may prevent tracing od the NMR signal of this particular arrestin conformation in the TMSiPhe detection range. Further experiments using nanodiscs as a substitute for the detergent used in the current experimental conditions will be of great help in further detailing the hanging conformation of arrestin, which is beyond the scope of the current manuscript. We have included these discussions in the main manuscript (page 9-10, lines 330-336).

5. In line 301-310, the authors claimed that β-arrestin-1/ppβ2V2R/Fab30 complex was dissociated by the addition of V2Rpp, and the active conformation still exists even after the dissociation. As the authors described in line 308-310, the growing evidences suggested that arrestins maintain their activity after the dissociation from receptor in cellular context. However, the reported rate constant of the reversal of the active conformation was 23 s (Nuber et al., Nature (2016) 661-664), which is much faster than the experimental time of the NMR measurement (40 min described in line 622). In addition, the reported stabilization of the arrestin activity after the dissociation from receptor requires non-GPCR partners such as phosphoinositide (Eichel et al., Nature (2018) 557, 381-386), which were not contained in the NMR sample. Therefore, the conformation of the β-arrestin-1 should be relaxed to the inactive form when β-arrestin-1 was fully dissociated from the ppβ2V2R and Fab30. There are the possibilities that the S3 resonance in the β-arrestin-1/ppβ2V2R/Fab30/V2Rpp sample was from the residual β-arestin-1 bound to ppβ2V2R and/or Fab30, or the irreversibly denatured or degradated β-arrestin-1 in the NMR sample.

**Reply:** We thank the reviewer for his valuable comments. We agree with the reviewer that the methods used in this manuscript could not provide a solid conclusion for the phosphopeptide competition experiment. Removing this part has little impact on the main conclusion of the current manuscript. We have therefore deleted the corresponding part.

6. In line 360-372, the authors claimed the high sensitivity of the 1H resonances from TMSiPhe groups by comparing with the sensitivity of the 19F resonances from 3,5- difluorotyrosine (F2Y) probe. The principle of the sensitivity gain should be thoroughly described here for clarity. In general, intrinsic sensitivity of 19F NMR signals is comparable with 1H NMR signals since the gyromagnetic ratio of 1H and 19F are comparable. It seems that the main reason of the higher sensitivity of the TMSiPhe groups than F2Y groups are not due to the kind of nuclei (19F vs 1H), but the number of magnetically equivalent spins (nine in TMSiPhe vs two in F2Y) and the slow relaxing nature of the methyl groups.

**Reply:**

We thank the reviewer for his valuable comments, which we agree with. We believe that the high-intensity proton signals are mostly contributed by two factors: (1) the nine equivalent H atoms in TMSiPhe and (2) the non-chemical shift anisotropy (CSA) effect of protons. Moreover, the upfield NMR signal of TMSiPhe over other proton NMR signals was convenient for detecting specific TMSiPhe probes.

(1) The nine equivalent protons of TMSiPhe significantly increased its signal and S/N ratio. In contrast, it is difficult to incorporate nine F atoms into one unnatural amino acid.

(2) The non-chemical shift anisotropy (CSA) effect of protons (Figure S6c): Because the hydrogen nucleus has a high magnetogyric ratio, the relative NMR sensitivity of the fluorine atom is 83.3% that of hydrogen with the same magnetic field. In addition, relaxation by chemical shift anisotropy (CSA) will adversely affect the NMR signal of the fluorine atom. The equation is as follows:

$R_{1\left（ \mathrm{CSA} \right）}$=$\frac{1}{T_{1\left( CSA \right)}}$=$\frac{2}{15}\gamma^{2}{B_{0}}^{2}({\sigma_{\parallel}-\sigma_{\perp})}^{2}\tau_{C}$

$\gamma$: Magnetogyric ratio

$B_{0}$: Magnetic field

$\sigma_{\parallel}-\sigma_{\perp}$: Chemical shift anisotropy

$\tau_{C}$: Correlation time

Since hydrogen atoms only have S orbital electrons, they have the same $\sigma_{\parallel}$ and $\sigma_{\perp}$, so the sensitivity will not be weakened by the CSA effect; however, F atoms have asymmetric P orbital electrons, and their $\sigma_{\parallel}$ and $\sigma_{\perp}$are different. Therefore, the CSA effect for the F atom widens the signal peak and weakens the signal. In particular, when the magnetic field strength or protein molecular weight is increased, it corresponds to a larger $B_{0}$ or $\tau_{C}$. Collectively, for a protein with a large molecular weight, the hydrogen atom will be better qualified.

We have included all these discussions in the revised manuscript (page 5-6, lines 182-187, in the results section; page 12, lines 423-426, in the discussion section; and Figure S6c in the supplemental materials).

**Reviewer comments, second version:**

Reviewer #1 (Remarks to the Author):

I believe that the authors have answered all of my concerns and questions and that the manuscript should be accepted for publication.


Reviewer #2 (Remarks to the Author):

The authors addressed most of my concerns and the manuscript has been much improved. I think that this version of the manuscript will be basically acceptable. However, there are minor points that should be addressed before publication.

1. In Figure S18 of the revised manuscript, the authors investigate the titration experiment of ppβ2V2R and demonstrate that the Kd is approximately 13.99 ± 2.60 μM, which is weaker than that of β-arrestin-1-V2Rpp interaction described in Figure 3 of the revised manuscript. The affinity of β-arrestin-1 for ppβ2V2R would be expected to be stronger than that for V2Rpp, since β-arrestin-1 can bind to both phosphorylated tail and the 7TM core of ppβ2V2R. It could be worth discussing the reason of the contradiction for clarity.

2. In line 332-334 of the revised manuscript, the authors claimed that β-arrestin-1 is entirely covered by the detergent in the structure of the ppβ2V2R/Gs/β-arestin-1/Fab30 supercomplex (Nguyen et al., Nat Struct. Mol. Biol. 26, 1123-1131 (2019)). However, as far as I examined, although the TM region of β2V2R in the supercomplex is covered by the detergent, the density of the detergent covering β-arrestin-1 are not well observed in the density map (EMDB ID EMD 9375 and EMD-9377). I think that it could be possible to considering the effect of detergent for the conformation of β-arrestin-1 in the hanging complex, but the structure of the supercomplex cannot be the evidence of the effect of detergent.

3. In line 182-187, line423-427, and Figure S6 of the revised manuscript, the chemical shift anisotropy effect for the signal intensity is described. The equation of R1(CSA) in Figure S6 should be replaced by that of R2(CSA), since the loss of signal intensity in this case is due to the transverse relaxation, not the longitudinal relaxation.

**Author rebuttal, second version:**

**Responses to reviewers’ comments**

Reviewer #1 (Remarks to the Author):

I believe that the authors have answered all of my concerns and questions and that the manuscript should be accepted for publication.

**Reply：**We thank the reviewer for his positive comments.

Reviewer #2 (Remarks to the Author):

The authors addressed most of my concerns and the manuscript has been much improved. I think that this version of the manuscript will be basically acceptable.

**Reply：**We thank the reviewer for his positive comments.

However, there are minor points that should be addressed before publication.
1. In Figure S18 of the revised manuscript, the authors investigate the titration experiment of ppβ2V2R and demonstrate that the Kd is approximately 13.99 ± 2.60 μM, which is weaker than that of β-arrestin-1-V2Rpp interaction described in Figure 3 of the revised manuscript. The affinity of β-arrestin-1 for ppβ2V2R would be expected to be stronger than that for V2Rpp, since β-arrestin-1 can bind to both phosphorylated tail and the 7TM core of ppβ2V2R. It could be worth discussing the reason of the contradiction for clarity.

**Reply：**We thank the reviewer for his valuable comments and helpful suggestions. The synthesized V2Rpp phospho-segment contained 8 fully phosphorylated residues. In contrast, the phospho-β2V2R used in our manuscript was prepared by co-expression of the receptor and GRK, which in general could not reach full phosphorylation at every site. We suspected that the different phosphorylation level is the cause for higher Kd of the phospho-β2V2R compared to V2Rpp phospho-segment. We have included corresponding description in the revised manuscript (page 10).

2. In line 332-334 of the revised manuscript, the authors claimed that β-arrestin-1 is entirely covered by the detergent in the structure of the ppβ2V2R/Gs/β-arestin-1/Fab30 supercomplex (Nguyen et al., Nat Struct. Mol. Biol. 26, 1123-1131 (2019)). However, as far as I examined, although the TM region of β2V2R in the supercomplex is covered by the detergent, the density of the detergent covering β-arrestin-1 are not well observed in the density map (EMDB ID EMD 9375 and EMD-9377). I think that it could be possible to considering the effect of detergent for the conformation of β-arrestin-1 in the hanging complex, but the structure of the supercomplex cannot be the evidence of the effect of detergent.

**Reply：**We thank the reviewer for his valuable comments and helpful suggestions. We have revised corresponding parts accordingly in the revised manuscript (page 11).

3. In line 182-187, line 423-427, and Figure S6 of the revised manuscript, the chemical shift anisotropy effect for the signal intensity is described. The equation of R1(CSA) in Figure S6 should be replaced by that of R2(CSA), since the loss of signal intensity in this case is due to the transverse relaxation, not the longitudinal relaxation.

**Reply：**We thank the reviewer for his valuable comments and we have revised corresponding parts accordingly by replacement of the R1(CSA) with the R2(CSA) in supplementary figure 7c.

**Reviewer comments, third version:**

**Author rebuttal, third version:**
